# Identification and expression analysis of pineapple sugar transporters reveal their role in the development and environmental response

**DOI:** 10.3389/fpls.2022.964897

**Published:** 2022-10-24

**Authors:** Beenish Fakher, Bello Hassan Jakada, Joseph G. Greaves, Lulu Wang, Xiaoping Niu, Yan Cheng, Ping Zheng, Mohammad Aslam, Yuan Qin, Xiaomei Wang

**Affiliations:** ^1^ Guangxi Key Lab of Sugarcane Biology, State Key Laboratory for Conservation and Utilization of Subtropical Agro-Bioresources, College of Agriculture, Guangxi University, Nanning, China; ^2^ Horticulture Research Institute, Guangxi Academy of Agricultural Sciences, Nanning Investigation Station of South Subtropical Fruit Trees, Ministry of Agriculture, Nanning, China; ^3^ Fujian Provincial Key Laboratory of Haixia Applied Plant Systems Biology, Key Laboratory of Genetics, Breeding and Multiple Utilization of Crops, Ministry of Education, Fujian Agriculture and Forestry University, Fuzhou, China

**Keywords:** pineapple, sugar transporter, fruit development, gene expression, circadian

## Abstract

In plants, sugars are required for several essential functions, including growth, storage, signaling, defense and reproduction. Sugar transporters carry out the controlled movement of sugars from source (leaves) to sink (fruits and roots) tissues and determine the overall development of the plant. Various types of sugar transporter families have been described in plants, including sucrose transporters (SUC/SUT), monosaccharide transporter (MST) and SWEET (from **“**Sugar Will Eventually be Exported Transporters**”**). However, the information about pineapple sugar transporters is minimal. This study systematically identified and classified 45 MST and 4 SUC/SUT genes in the pineapple genome. We found that the expression patterns of sugar transporter genes have a spatiotemporal expression in reproductive and vegetative tissues indicating their pivotal role in reproductive growth and development. Besides, different families of sugar transporters have a diel expression pattern in photosynthetic and non-photosynthetic tissues displaying circadian rhythm associated participation of sugar transporters in the CAM pathway. Moreover, regulation of the stress-related sugar transporters during cold stress indicates their contribution to cold tolerance in pineapple. Heterologous expression (yeast complementation assays) of sugar transporters in a mutant yeast strain suggested that SUT1/2 have the ability to transport sucrose, and STP13, STP26, pGlcT-L2 and TMT4 are able to transport glucose, whereas SWEET11/13 transport both sucrose and fructose. The information provided here would help researchers further explore the underlying molecular mechanism involved in the sugar metabolism of pineapple.

## Introduction

One of the key characteristics of vascular plants is the biosynthesis of sugars in photosynthetically active tissues (source) and the transport of excess sugars to non-photosynthetic plant parts (sink) (Reuscher et al., 2014). Sugar transporters help move sugars (monosaccharides, polyols, or sucrose) through the phloem, the tissue responsible for long-distance sugar transport, and load and/or unload sugars to the phloem vessels or companion cells (Buttner, 2007; Schulz et al., 2011; Saddhe et al., 2021; Yoon et al., 2021; Xue et al., 2022). Most sugar transporters belong to the major facilitator superfamily (MFS), characterized by 12 transmembrane domains (TMDs). According to the substrates it transports, sugar transporters can be further categorized into the monosaccharide transporters (MSTs) and sucrose transporter (SUT/SUC) families (Reinders et al., 2012). Based on their substrate specificities and sequence features, the MSTs are further divided into seven subfamilies, including Sugar Transport Proteins (STP/HT), Early Response to Dehydration-6/Sugar Facilitator Protein (ERD-6/SFP), Polyol Monosaccharide/PolyolTransporter (PMT/PLT), plastidic Glucose Like Transporter (pGlcT), vacuolar Glucose Transporter (vGT), Tonoplast Monosaccharide Transporter (TMT) and Inositol Transporter (INT/ITR) (Sauer and Stolz, 1994; Sherson et al., 2003; Wormit et al., 2006; Buttner, 2007; Reinders et al., 2012; Reuscher et al., 2014; Nino-Gonzalez et al., 2019). In addition to sugar transporters belonging to the MFS superfamily, a new type of sugar transporters, SWEET (from “Sugars Will Eventually be Exported Transporters”), was recently identified in *Arabidopsis* (Chen et al., 2010). Contrary to MFS sugar transporters, which have 12 TMDs, SWEETs only have seven TMDs composed of two internal triple-helix bundles connected by a linker TMD (Chen et al., 2010). Although SWEET transporters were initially found in plants, their homologs have been found in many other organisms. In addition to the transport of mono- and di-saccharides, SWEETs have been implicated in the transport of phytohormones such as gibberellin (GA) (Chen et al., 2015; Kanno et al., 2016; Wang et al., 2022; Xue et al., 2022).

Plant sugar transporters have numerous functions in various physiological processes, including phloem loading, nectar secretion, seed nutrient filling and stress response (Anjali et al., 2020; Saddhe et al., 2021; Slawinski et al., 2021; Ji et al., 2022). In Arabidopsis, phloem loading of sucrose is mediated by SWEET11 and SWEET12; in the process, sucrose is initially transported out of mesophyll cells to the apoplast and then imported into companion cells by SUT1 (Chen et al., 2012). SWEET11, SWEET12 together with SWEET15, regulate sucrose transport from the seed coat to the embryo through the endosperm (Chen et al., 2015). Sugar transporters also regulate flower development and are required for normal reproductive organ development (Yu et al., 2005; Chu et al., 2006; Sun et al., 2019). Moreover, sugar transporters participate in adaptative responses; for example, SUTs and STPs are upregulated at the time of stress; otherwise, they show minimal expression during optimal growth conditions (Gong et al., 2015; Sambe et al., 2015; Cai et al., 2019; Kong et al., 2019; Cai et al., 2020). In particular, vacuolar transporters (e.g., INT, vGT, TMT, and ERD6) are essential for accumulating sugar-based osmolytes in vacuoles during adaptation to stress (e.g., cold, drought and salinity, etc.) and fruit ripening stages (Wormit et al., 2006; Buttner, 2007; Klemens et al., 2013; Gong et al., 2015). Several SWEETs get upregulated during cold stress in a variety of crops, e.g., cotton (Li et al., 2018), apple (Yang et al., 2020), banana (Miao et al., 2017) and tea (Yue et al., 2015; Wang et al., 2018). Furthermore, sugar transporters are differentially expressed during low temperatures in *Arabidopsis.* For example, a higher level of freezing tolerance is shown by the *sweet11sweet12* mutant (Le Hir et al., 2015). The role of sugar transporters in pathogen-plant interactions is also crucial (Chen et al., 2015; Breia et al., 2021; Ji et al., 2022).

Pineapple (*Ananas comosus L*.) is the only edible member of the *Bromeliaceae* family. It is enjoyed as a flavorful fruit with a distinct aroma and is one of the most exported fruit crops. Monocot plants like pineapple offer a peculiar yet complex mechanism of sugar production (photosynthesis) and allocation. The strictly regulated route of sugar transport is essential for the proper development of plant organs such as root tips, pollen and seeds and the storage of large amounts of sugars in other tissues like fruit (Paulsen et al., 2019). Thus, the seamless functioning of sugar transporters is vital for plant growth and development (Baud et al., 2008). Although the sugar transporter families have been studied in several plants, including rice, *Arabidopsis*, tomato, and grape (Johnson and Thomas, 2007; Afoufa-Bastien et al., 2010; Reuscher et al., 2014) and some sugar transporter families have been described in pineapple (Antony et al., 2008; Guo et al., 2018), a detailed inventory of the pineapple sugar transporters is required to utilize them in the crop improvement program.

This study presents a genome-wide identification and expression analysis of pineapple sugar transporters during development, circadian and cold stress conditions. A total of 45 MST and 4 SUC/SUT were identified and comprehensively studied for the physicochemical properties, chromosome location, gene structure, phylogenetic relationship, motif, and expression analysis. We found that the pineapple sugar transporter genes are differentially regulated during cold stress and spatiotemporally expressed in reproductive organs and circadian rhythm. Moreover, heterologous expression was carried out in defective yeast strain to determine the potential functions of the sugar transporter genes. The present study summarized the current status of the sugar transport system in pineapple, which would provide new avenues to explore the physiological relevance and biotechnological potential of using these sugar transporters in breeding programs.

## Materials and methods

### The sequence retrieval of pineapple sugar transporters

The sequence information about pineapple was obtained from the Phytozome (https://phytozome.jgi.doe.gov/pz/portal.html). Protein sequences from *Arabidopsis* and rice (*Oryza sativa*) were downloaded from TAIR (http://www.arabidopsis.org) and the rice data center of China (http://www.ricedata.cn/gene/index.htm), respectively. Sequence details for all other species were downloaded from NCBI (https://www.ncbi.nlm.nih.gov/). The sugar transporter domain Hidden Markov Model (HMM) profiles of Pfam Sugar_tr domain (PF00083) was acquired from Pfam (http://pfam.sanger.ac.uk/) (El-Gebali et al., 2019). The screening of the homologous protein sequence was done using BLASTP with a set threshold of e-value < 1e-5. Finally, the selected candidates were verified utilizing SMART (http://smart.embl-heidelberg.de/) (Letunic et al., 2012). ExPASy (http://ca.expasy.org/prosite/) proteomics server was used for the physicochemical properties of sugar transporter genes in pineapple.

### Phylogenetic analysis

The protein sequences were aligned and the phylogenetic tree was built with MEGA (version 11.0) using the neighbor-joining (NJ) method (Tamura et al., 2013). The NJ tree was built with the **‘**pairwise deletion**’** option and the **‘**Poisson correction**’** model, and the internal branch reliability was assessed with a bootstrap test with 1000 iterations (Kumar et al., 2016). All the sequences used in the study are listed in the **Additional File S1**.

### Chromosomal location, gene structure, motif analysis and subcellular localization

The chromosome location information for the sugar transporter genes was gathered from Phytozome (https://phytozome.jgi.doe.gov/pz/portal.html) and used to map them by Mapchart (Version2.1) (Voorrips, 2002). Besides, the gene structure analysis was performed using the online Gene Structure Display Server (http://gsds.cbi.pku.edu.cn/) (Guo et al., 2007; Hu et al., 2015). The software Multiple Em for Motif Elicitation (MEME) was employed to identify conserved motifs in pineapple sugar transporter genes (Bailey et al., 2009). WoLFPSORT algorithm was exploited to predict subcellular localization of sugar transporter proteins (http://wolfpsort.seq.cbrc.jp), and TMHMM Server v.2.0 was used to predict transmembrane helical domains (http://www.cbs.dtu.dk/services/TMHMM/).

### RNA-Seq analysis of pineapple sugar transporter genes

Transcriptome data of pineapple flower and fruit development stages (Wang et al., 2020) was used to investigate the spatiotemporal expression level of sugar transporter genes. Besides, the RNA-Seq data (bioproject PRJNA331052) generated from cold stressed and control pineapple plantlets (Chen et al., 2016) and PRJNA305042 for diel expression (generated from the youngest physiologically mature leaf) (Ming et al., 2015) were also analyzed to explore the expression of sugar transporter genes. Briefly, the sequencing reads were aligned to the pineapple genome using TopHat v2.1.1. The FPKM values were estimated using Cufflinks v2.2.1 software and Cuffdiff. Using the log2 (FPKM + 1) values, heatmaps for the sugar transporter genes were generated by pheatmap package in R.

### Plant materials

The tissue culture-raised pineapple plants of MD2 variety were used as previously described (Aslam et al., 2020; Aslam et al., 2022). Different pineapple sample tissues, including root, flower, and fruit stages: early (fruit1), middle (fruit3), and late (fruit7), were harvested and immediately frozen in liquid nitrogen and stored at −80°C until RNA isolation.

### Quantitative real-time (RT-qPCR)

Total RNA from desired tissues was isolated using the RNeasy kit (Qiagen, MD, USA). The cDNA was prepared using one µg of total RNA as instructed in the kit protocol (TransGen cDNA preparation kit). The cDNA was then used for RT-qPCR with 2X qPCR superMix (TransGen) in a 20 μL reaction on a Bio-Rad CFX96 Touch™ real-time PCR machine (Bio-Rad, Singapore). The cycling parameters were: 95°C for 30 s; 40 cycles of 95°C for 10 s and 60°C for 15 s. The fold change in the gene expression was determined using the *EF1*α gene as the internal control using Livak method (2^−ΔΔCT^) as described earlier (Livak and Schmittgen, 2001; Aslam et al., 2019; Jakada et al., 2019; Aslam et al., 2020). Three biological replicates and at least three independent technical replicates were performed in each condition. To analyze statistical significance, a two-tailed Student**’**s t-test was used *** indicates p < 0.001, ** indicates p < 0.01 and and * indicates p < 0.05. The primers used in this study are listed in the **Additional File S2**.

### Heterologous expression of sugar transporters in yeast

For the heterologous expression, the open reading frames (ORFs) of pineapple *SUT1*, *SUT2*, *STP13*, *STP26*, *pGLcT*-L2, *TMT2*, *SWEET4/11/13/18* and *ZIFL2a* were PCR amplified using cDNA prepared from the RNA of fruit tissue. The amplified fragments were inserted in the yeast expression vector pGBKT7 by infusion method and verified by sequencing. The final vectors, including the empty vector (as control), were then introduced into the yeast defective yeast strain EBY.VW4000 using LiAc/PEG mediated transformation (Gietz and Schiestl, 2007). EBY.VW4000 cannot transport monosaccharides and sucrose due to multiple mutations in sugar-sensing and transporter genes. Yeast transformants were incubated on a selective dropout (SD, -Trp) medium (www.coolaber.com) containing 2% maltose for 2–3 d at 30°C. For yeast complementation growth assays, overnight grown liquid cultures of transformants were centrifuged, resuspended to an OD_600_ of 0.2, and grown in serial dilutions on SD (-Trp) media containing either 2% maltose (positive control), 2% sucrose, 2% glucose or 2% fructose. The plates were incubated at 30°C and the growth was documented after 2-4 d.

### Statistical analysis

A two-tailed Student**’**s t-test was used to analyze statistical significance, and the results are represented as the mean values ± SE of three biological replicates.

## Results

### Identification of sugar transporter gene family in pineapple

A total of 49 sugar transporter gene sequences belonging to MST (45) and SUC/SUT (4) were identified in the pineapple genome. For identification, structure and phylogenetic tree, we mainly focused on MST and SUC/SUT sugar transporters as these details for pineapple SWEETs have already been described previously (Guo et al., 2018). Nearly 60% of sugar transporters had a length of about 500 amino acids. AcSTP5a had an exceptionally longer size of 841 aa, and AcSUT3 had the smallest size (274 aa). The molecular weight of pineapple sugar transporters ranged from 29.137 KDa (AcSUT3) to 92.244 KDa (AcSTP5a), with an average range of 57.42 KDa. The isoelectric point values ranged between 5-10; however, members of vGT and TMT exhibited the least isoelectric point value between 5-7, and it was highest for AcpGLCT1 (10.27). The aliphatic index for the sugar transporter proteins ranged from 93.69 (AcSUT3) to 127.16 (AcvGT2), and the average value of most of the sugar transporter protein (approximately 93%) was 107. The instability index of 60% of sugar transporter proteins was below 45 except AcPMT3/5/7 and AcpGLCT1 with values above 45. The GRAVY index of sugar transporters was predicted between 0.112 (AcpGLCT1) to 0.846 (AcvGT2), with an average range value of 0.506 (**Table 1**). The subcellular localization of the majority of sugar transporters was in the plasma membrane, followed by the vacuolar membrane (AcSTP1, AcSTP4, AcSTP5b, AcSTP5c, AcSTP5d, AcSTP5e, AcSTP6b, AcSTP7, AcSTP8 and AcSTP10) and chloroplast (AcpGlcT2, and AcvGT1) and contained 6 to 12 transmembrane helical domains (**Table 1**).

**Table 1:** Comprehensive classification and characteristics of sugar transporters identified in pineapple.

Gene name	Gene ID	Length (aa)	MW (Da)	pI	I.I.	A.I.	GRAVY	SUB-LOC.	TMH
AcSUT1	Aco004135.1	521	55.272	6.7	33.34	96.142	0.491	PM	7
AcSUT2	Aco004131.1	526	55.836	8.48	32.6	102.6	0.564	PM	10
AcSUT3	Aco009281.1	274	29.137	8.3	36.96	93.69	0.381	PM	6
AcSUT4	Aco000269.1	582	62.267	9.41	34.6	108.97	0.578	PM	12
AcSTP1	Aco024297.1	525	58.169	7.53	39.22	106.38	0.501	V	11
AcSTP2	Aco026106.1	519	56.977	8.38	36.22	105.99	0.546	PM	12
AcSTP3	Aco026103.1	517	56.634	9.28	36.67	106.03	0.545	PM	12
AcSTP4	Aco021163.1	502	55.483	9.49	40.77	101.75	0.254	V	10
AcSTP5a	Aco028869.1	841	92.244	6.36	44.55	103.54	0.362	PM	12
AcSTP5b	Aco007397.1	515	56.004	8.59	37.22	106.58	0.614	V	12
AcSTP5c	Aco027220.1	515	55.99	8.59	37.39	106.41	0.61	V	12
AcSTP5d	Aco014729.1	517	54.81	9.01	33.87	100.7	0.589	V	12
AcSTP5e	Aco014753.1	534	57.395	9.93	39.23	103.58	0.449	V	10
AcSTP6a	Aco014415.1	535	57.698	9.26	33.47	108.36	0.557	PM	11
AcSTP6b	Aco009133.1	521	56.925	9.31	37.32	102.76	0.521	V	11
AcSTP7	Aco007400.1	525	57.317	9.51	38.04	102.51	0.489	V	11
AcSTP8	Aco008491.1	546	59.903	8.81	42.06	100.33	0.452	V	11
AcSTP10	Aco014002.1	515	55.626	8.19	32.52	110.76	0.556	V	11
AcSTP13	Aco011682.1	473	52.412	9.59	38.27	105.67	0.435	PM	9
AcSTP23	Aco018637.1	554	59.056	9.19	39.88	104.78	0.511	PM	10
AcSTP25	Aco006734.1	523	57.113	8.94	38.74	112.03	0.512	PM	11
AcSTP26	Aco016897.1	521	56.636	9.37	41.39	106.53	0.516	PM	11
AcERD-4L	Aco014912.1	440	47.575	5.34	42.65	106.98	0.638	PM	10
AcERD-6L1	Aco005379.1	532	57.771	8.54	41.38	112.18	0.549	PM	12
AcERD-6L2	Aco006192.1	496	52.684	5.99	41.99	115.65	0.626	PM	11
AcERD-6L3	Aco014914.1	422	45.325	8.08	36.57	108.32	0.56	PM	9
AcERD-6L4	Aco018778.1	422	45.307	8.6	34.65	115.05	0.78	PM	11
AcZIFL-2a	Aco019261.1	484	53.334	8.39	38.3	104.21	0.503	PM	11
AcZIFL-2b	Aco015001.1	503	55.205	8.35	40.8	107	0.49	PM	12
AcZIFL-2c	Aco014996.1	491	53.529	7.99	44.6	105.13	0.517	PM	11
AcPMT1	Aco003836.1	526	55.924	9.17	41.41	109.26	0.538	PM	12
AcPMT2	Aco012301.1	563	60.236	9.3	37.35	102.04	0.39	PM	10
AcPMT3	Aco012302.1	493	52.954	9.46	45.25	108.5	0.436	PM	10
AcPMT4	Aco010302.1	536	57.53	5.43	37.27	115.49	0.57	PM	10
AcPMT5	Aco000080.1	556	59.6	9.16	47.6	99.19	0.266	PM	10
AcPMT6	Aco016335.1	533	57.306	6.69	40.82	114.48	0.51	PM	6
AcPMT7	Aco016336.1	457	49.076	6.62	46.08	118.23	0.624	PM	9
AcITR1	Aco008059.1	511	54.372	5.47	36.75	115.3	0.651	V	12
AcITR2	Aco005101.1	576	62.52	8.73	38.59	101.09	0.385	PM	12
AcITR3	Aco025488.1	571	61.392	8.77	38.8	104.92	0.439	PM	12
AcpGlcT1	Aco026982.1	744	78.904	10.27	55.84	94.88	0.112	PM	9
AcpGlcT2	Aco001011.1	407	43.266	6.19	39.46	108.99	0.369	Chlo	6
AcpGlcT-L1	Aco004425.1	463	49.492	6.99	42.96	106.8	0.594	PM	10
AcpGlcT-L2	Aco012247.1	502	54.997	5.71	39.4	106.18	0.615	PM	12
AcTMT1	Aco015779.1	749	80.459	5.19	43.29	109.59	0.381	PM	11
AcTMT2	Aco011916.1	754	80.88	5.14	43.08	108.1	0.394	PM	11
AcTMT3	Aco013008.1	720	78.158	5.16	36.73	108.56	0.416	PM	11
AcvGT1	Aco026737.1	504	53.61	6.23	40.83	120.4	0.59	Chlo	9
AcvGT2	Aco017005.1	504	53.444	6.62	37.31	127.16	0.846	PM	12

PM, plasma membrane; Chlo, chloroplasts; V, Vacuole.

**Figure 1:**
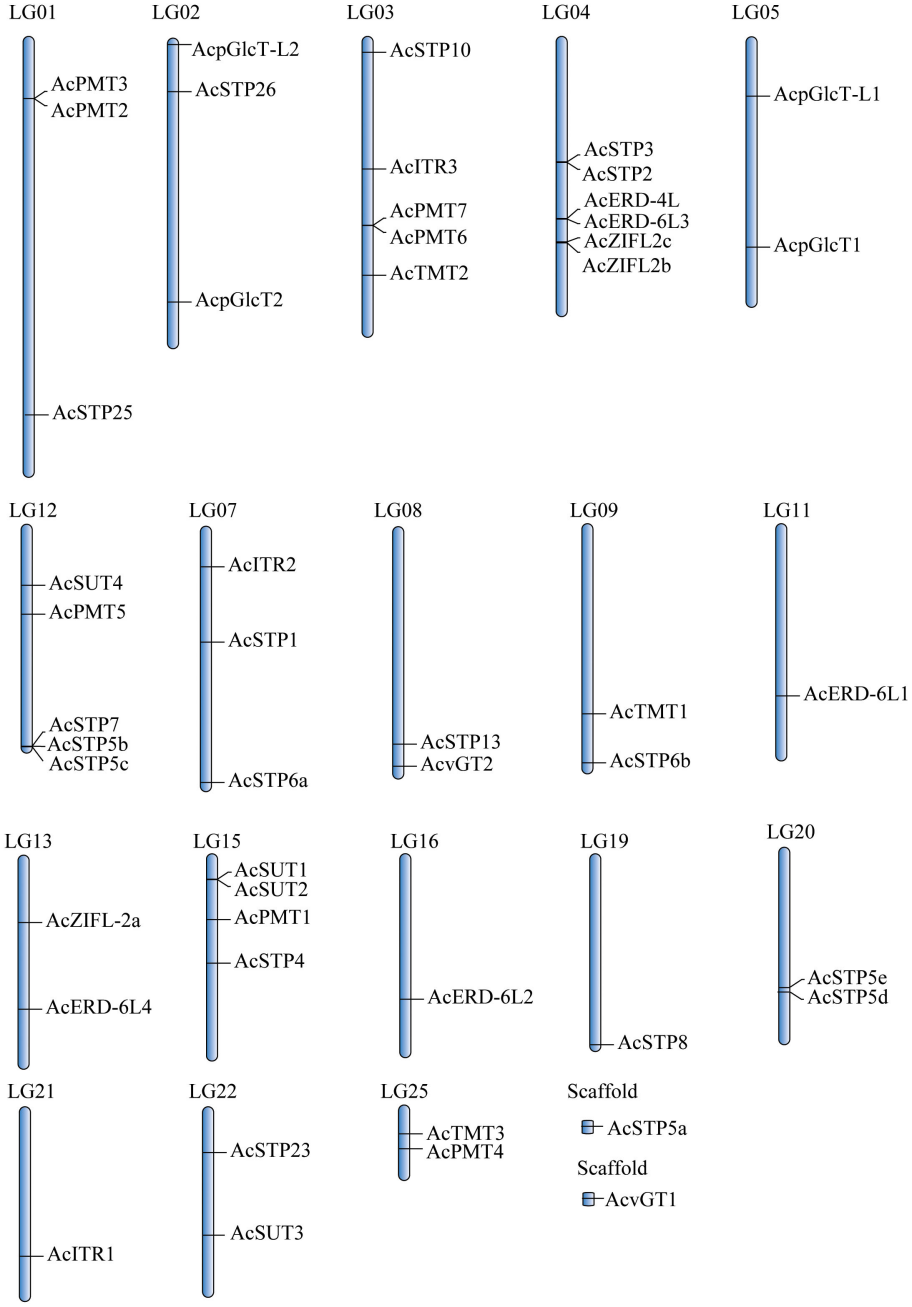
The distribution of sugar transporter genes in pineapple (*Ananas comosus*) genome.

The 49 pineapple sugar transporter genes were distributed on 18 chromosomes except for *AcvGT1* and *AcSTP5a*, which were identified as scaffolds (**Figure 1**). The 18 *AcSTPs* were located on 11 chromosomes, whereas 8 *ERDs* were clustered on 4 chromosomes. Chromosome 4 had the most sugar transporter genes (*AcSTP2/3, AcERD-4L/-6L3, AcZIFL2b/2c*), and nearly 77% of the sugar transporter genes were located in distal and proximal regions of chromosomes. None of the sugar transporter genes was located on chromosomes 6, 10, 14, 17, 18, 23 and 24. *AcERD-6L1/L2, AcSTP8*, and *AcITR1* were located individually on chromosomes 11,16,19 and 20, respectively (**Figure 1**).

### Gene structure and motif composition of pineapple sugar transporters

**Figure 2:**
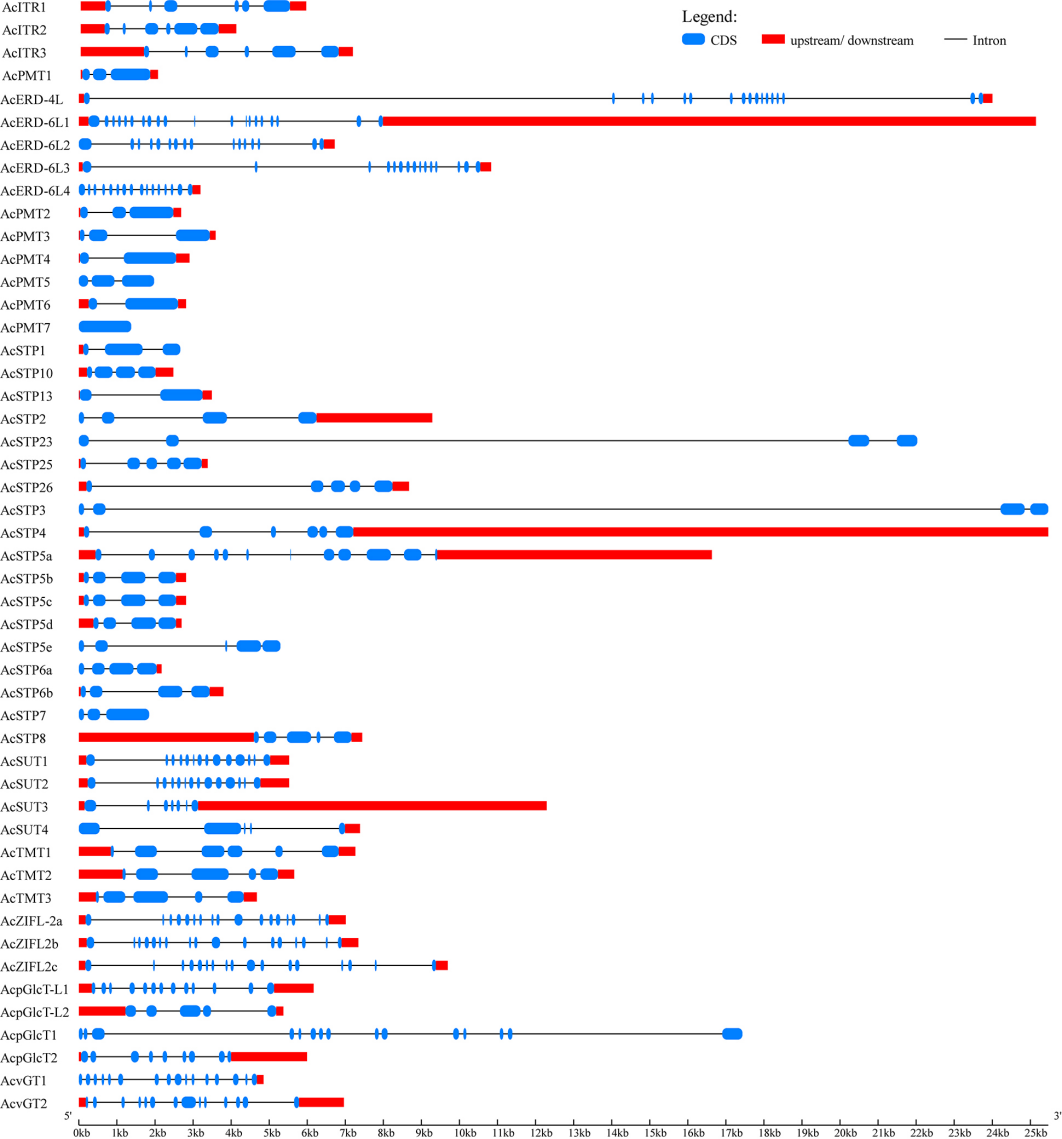
Exon–intron structure of sugar transporter genes identified in pineapple. The gene models of sugar transporters are shown in a graphic representation using the online Gene Structure Display Server (GSDS 2.0). Introns are represented as black lines; blue boxes show coding sequences, and red boxes represent upstream or downstream regions.

The intron/exon organization and conserved motifs of all the identified pineapple sugar transporters were studied to acquire insights into their evolution. The members of *AcvGT, AcERD-6, AcSUT* and *AcpGLcT* were found intron rich (n≥17) (**Figure 2**). However, intron number varied widely among sugar transporter genes. *AcPMT* members were intron poor compared to other subfamilies; for instance, *AcPMT4/6* and *AcSTP13* genes comprised only one intron, while *AcPMT7* had no intron with the exception of 3 introns in A*cSTP3* and *AcSTP23* had the largest intron length (**Figure 2**).

**Figure 3:**
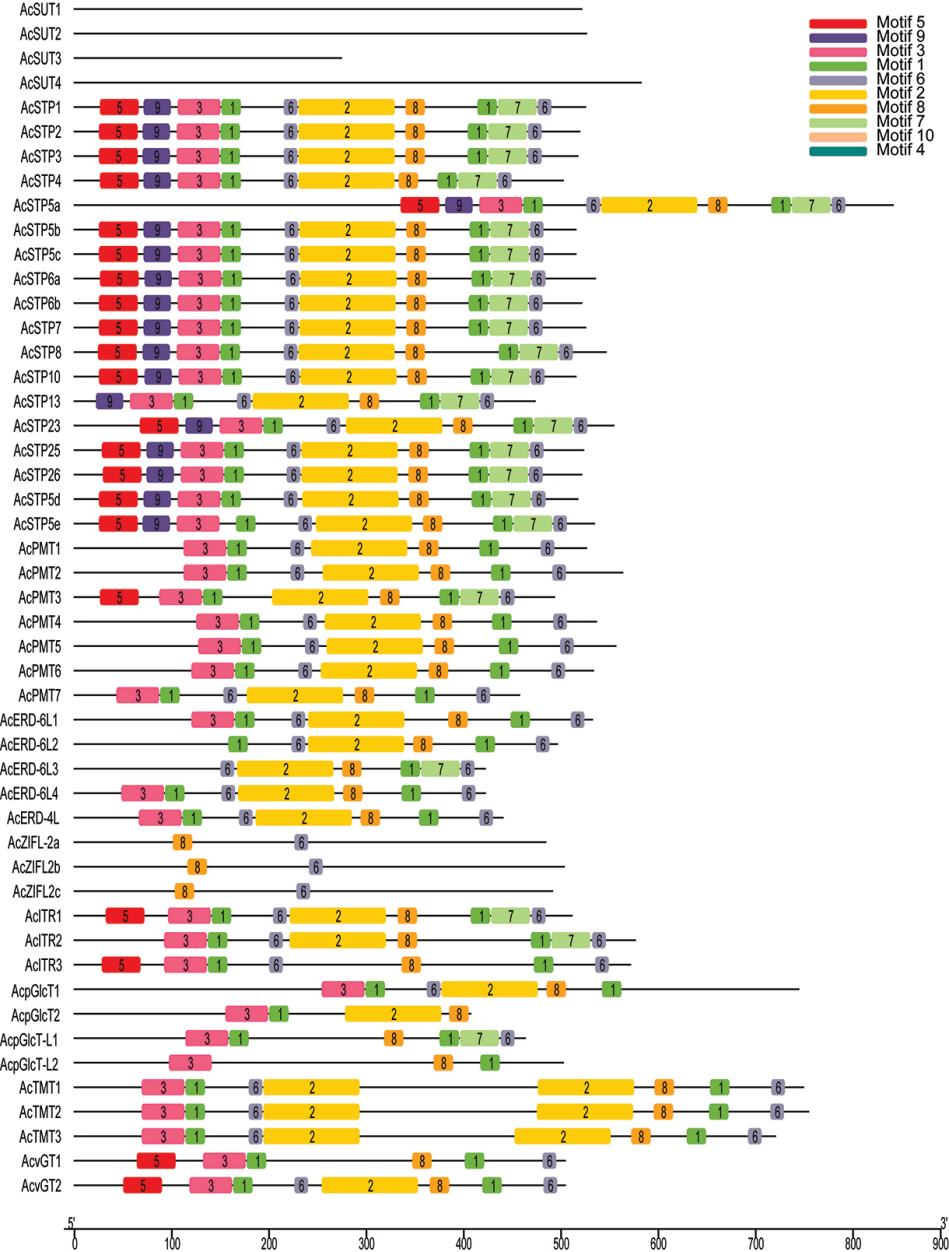
Conserved motifs and domains in sugar transporter genes of pineapple. Distribution of conserved motifs in the sugar transporter genes. The different-colored boxes represent different motifs and their position in each sugar transporter protein sequence.

Further, the structural features of sugar transporter genes were investigated by studying the conserved motifs encoded by their proteins. In total, 10 motifs were identified, and the arrangement of the motifs among each subfamily member was found highly similar, suggesting a functional similarity within the same family. The most common motifs at the N-terminal were motif 3 and motif 5, and motif 6 at C-terminal (**Figure 3**).

### Phylogenetic analysis of sugar transporter proteins

**Figure 4:**
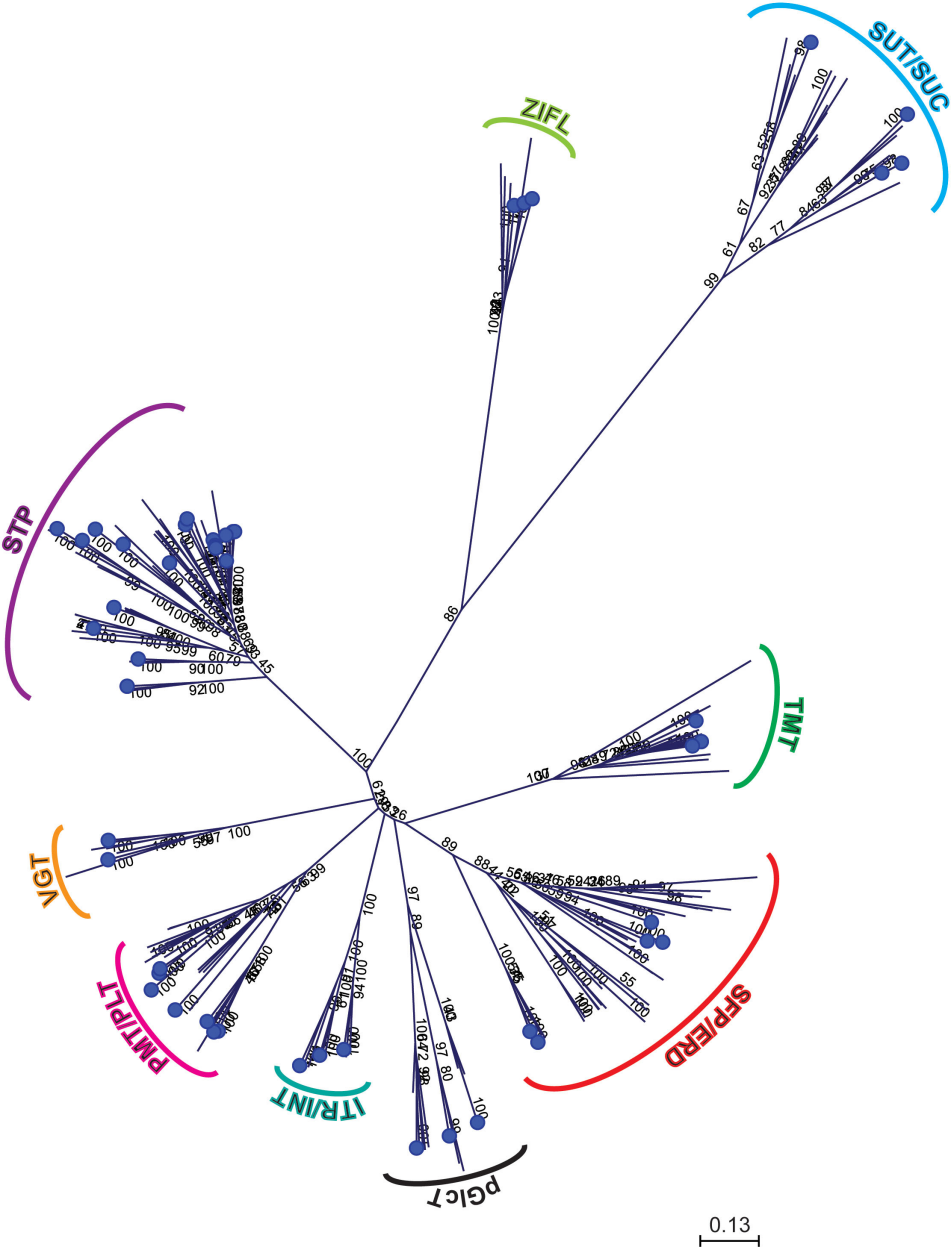
Phylogenetic relationship of sugar transporters among representative monocotyledons and dicotyledons. The protein sequences were used to build the phylogenetic tree of sugar transporters among representative monocots and eudicots. Sugar transporters proteins of pineapple are marked with blue circles.

**Figure 5:**
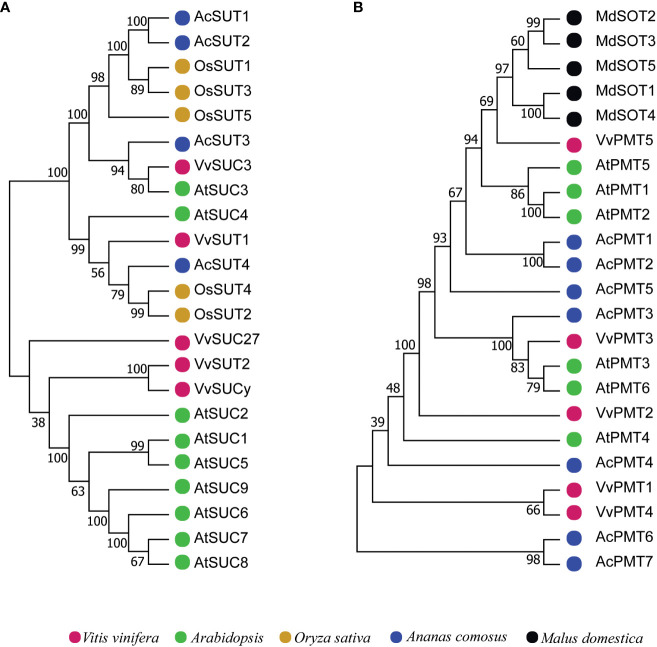
Phylogenetic tree of sugar transporters subfamilies among representative monocots and eudicots. The trees were built with the neighbor-joining algorithm using protein sequences. **(A)** The phylogenetic tree of the SUC/SUT subfamily. **(B)** The phylogenetic tree of the PMT subfamily. Sugar transporters genes of pineapple in the phylogenetic trees are marked with blue, and the sugar transporters genes of other species are marked with different colors as labeled at the bottom.

**Figure 6:**
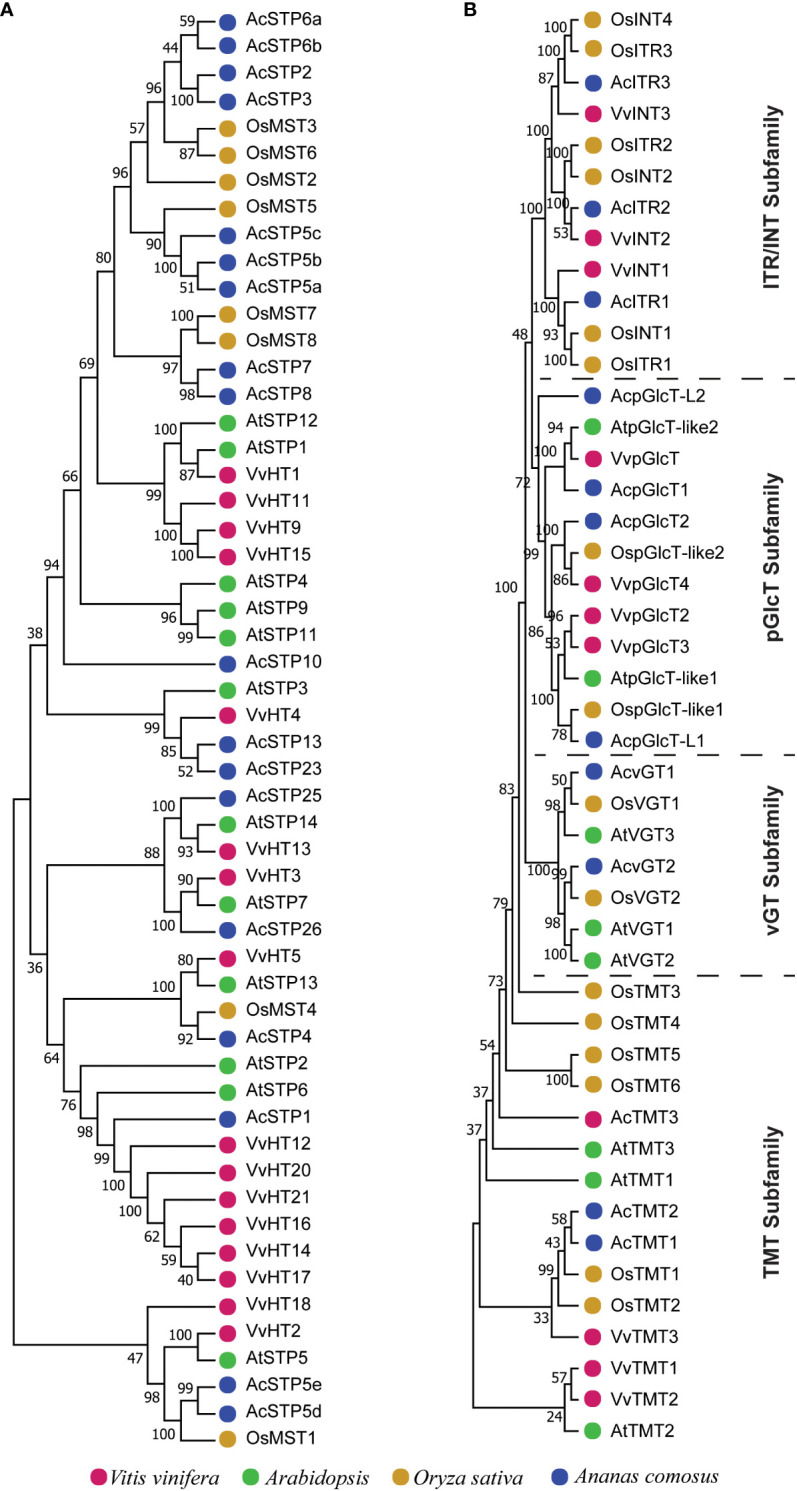
Phylogenetic tree of sugar transporters subfamilies. The trees were built with the neighbor-joining algorithm using protein sequences. **(A)** The phylogenetic tree of the STP subfamily. **(B)** The phylogenetic tree of the TMT, vGT, pGlcT and ITR/INT subfamilies. Sugar transporters genes of pineapple in the phylogenetic trees are marked with blue, and the sugar transporters genes other species are marked with different colors as labeled at the bottom.

Different phylogenetic trees were constructed using protein sequences from pineapple, *Arabidopsis*, *Vitis vinifera* and *Oryza sativa* to investigate the evolution of sugar transporter orthologs in different plant species (**Figures 4**–**6** and **Additional Figure 1**). The sugar transporters in pineapple were divided into different sub-families such as SUC/SUT, STP, SFP/ERD6-like and vGT. The paralogs of each subfamily formed a separate group (**Figure 4**). The phylogenetic analysis showed that two paralogs, AcSUT1 and AcSUT2, were close to OsSUT1, while AcSUT3 shared homology with VvSUC3 and clustered together in SUT2/SUC3 clade. The AcSUT4 shared homology with OsSUT4 and grouped separately in the SUT4/SUC4 clade, and none of the pineapple SUT was close to *Arabidopsis* SUCs (**Figure 5A**). Among the pineapple PMTs, AcPMT 1/2/5 formed a separate group, while AcPMT3 clustered together with AtPMT3/6 and VvPMT3. AcPMT4/6/7 shared homology with AtPMT4 and VvPMT1/2/4 and were grouped in a subgroup. Pineapple PMT members did not show close homology with the apple sorbitol transporters; those are considered a major transporting polyol in apple (**Figure 5B**) (Watari et al., 2004). The STPs phylogenetic analysis of pineapple with the STPs of rice, *Arabidopsis* and *Vitis* showed that AcSTP7/8 share homology with OsMST7/8, while AcSTP5a/5b/5c share homology with OsMST5. Besides, AcSTP5d/5e were placed together with OsMST1, and AcSTP4 showed similarity with OsMST4, whereas AcSTP1/13/23/25/26 were placed together with grape hexose transporters (HTs) in different subgroups (**Figure 6A**). Among the protein sequences of the ERD6/SFP subfamily, AcERD6L1/L2 formed a separate group with the grape SFPs and AtERD6. Whereas pineapple ZIFL formed another group (clade) with the rice and *Arabidopsis* ZIFLs (**Additional Figure 1**). At the same time, a phylogenetic tree consisting of smaller subfamilies (vGTs, TMTs, INTs and pGLCTs) was also constructed. Similar to rice and *Arabidopsis*, the vGT represented the smallest sugar transporter subfamily with two members (AcvGT1 and AcvGT2) in pineapple. AcTMT1 and AcTMT2 and AcITR1 and AcITR3 were closer to *Os*TMT1 and *Os*TMT2, OsITR1 and OsITR3, respectively, while AcITR2 showed homology with VvINT2. The pineapple pGLCT proteins were clustered separately with rice and grape pGLCTs in a separate group (**Figure 6B**).

### Expression profile of pineapple sugar transporter genes in reproductive tissues

**Figure 7:**
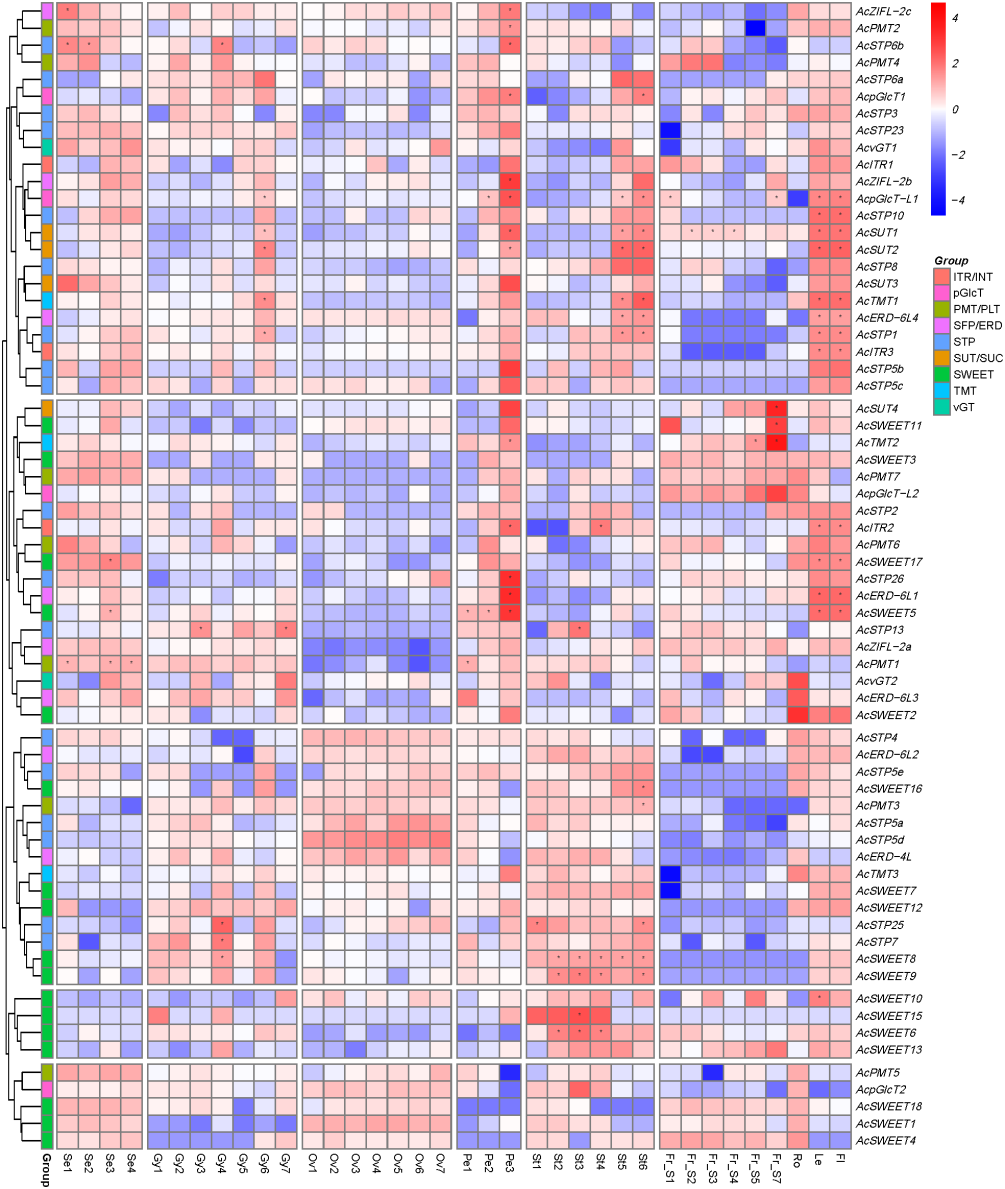
Expression profile of pineapple sugar transporter genes during different stages of reproductive development. The heatmap was created based on the log2 (TPM + 0.01) value of the genes and normalized by row. Red color represents a high transcript abundance, and blue represents low transcript abundance, and asterisks (*) represent the FKM values above 100. The right side of the figure shows the scale. Details of the samples are mentioned at the bottom of each lane: sepal Se1–Se4, gynoecium Gy1- Gy7, ovule Ov1–Ov7, petal Pe1–Pe3, stamen St1–St6, fruit ‘Fr_S1–Fr_S7’, where ‘S’ is the abbreviation for ‘stage’.

To understand the contributions of sugar transporter genes in reproductive development, RNA-seq data generated from early to mature stages of pineapple floral and fruit samples, including sepal (stages Se1-Se4), gynoecium (stages Gy1-Gy7), ovary (stages Ov1-Ov7), petal (stages Pe1-Pe3), stamen (stages St1-St6) and fruit (stages Fr_S1-Fr_S7) was analyzed (**Figure 7**). The result showed that *AcPMT* members had higher expression in most of the stages of sepals, along with *AcSWEET1/3/4/17/18*, *AcZIFL2a/2b/2c*, *AcSTP3/23/26*, *AcSUT3/4*, *AcpGLCTL2* and around 25% of sugar transporter genes had low expression in sepal developmental stages. Expression of *AcpGLCT2, AcZIFL2c, AcSTP2/3/6a/13/23, AcSUT1/2/3, AcSWEET2/5* and *AcTMT2* was high, and *AcSWEET1/4/6/18* was lower in petal developmental stages. Gynoecium exhibited moderate to higher expression of *AcSTP3/6a/13/23*, *AcvGT1/2, AcZIFL2b*, *AcERD6-L3, AcSWEET12, AcPMT1, AcTMT2* and *AcpGLCT1/-L2*, while *AcSTP26* and *AcSWEET1/4* had a lower expression in gynoecium. However, *AcSWEET4* started increasing in later stages of gynoecium development and continued to be expressed in the ovary, stamens, and fruit. Around 56% of sugar transporter genes had stage-specific expression or showed constitutively high expression in stamens. *AcERD-6L2/-6L4, AcSTP5e/7/25, AcTMT3, AcITR3*, and *AcSWEET6/7/8/9/10/12/13/15/16* expressed higher in most stages of stamen development. Specifically, *AcSUT1/2/3, AcTMT1*, *AcITR1/2/3, AcpGLCT-L1, AcSTP1/5c/6a/8/10* were expressed in later stages of stamen development. While, *AcSTP26*, *AcERD*-*6L1*/-*6L3*, *AcZIFL2c*, *AcSUT4*, *AcSWEET2*/*11*/*17*, *AcTMT2*, *AcpGLCT-L2* had low expression in the stamen. *AcSTP26*, *AcSUT4*, *AcTMT2*, *AcSWEET1/3/4/11/13/18*, *AcPMT7*, *AcpGLCTL2*, *AcZIFL2a* had moderately high expression in fruit developmental stages. However, *AcSUT4*, *AcTMT2*, *AcpGLCT*-*L1*/-*L2*, *AcZIFL2a* were expressed predominantly in the later stages of fruit or at the ripening stage (Fr_S7). Nearly 38% of sugar transporter genes showed very low expression in all the fruit stages (**Figure 7**).

### Pineapple sugar transporter genes show diel expression pattern in photosynthetic and non-photosynthetic leaf segments

**Figure 8:**
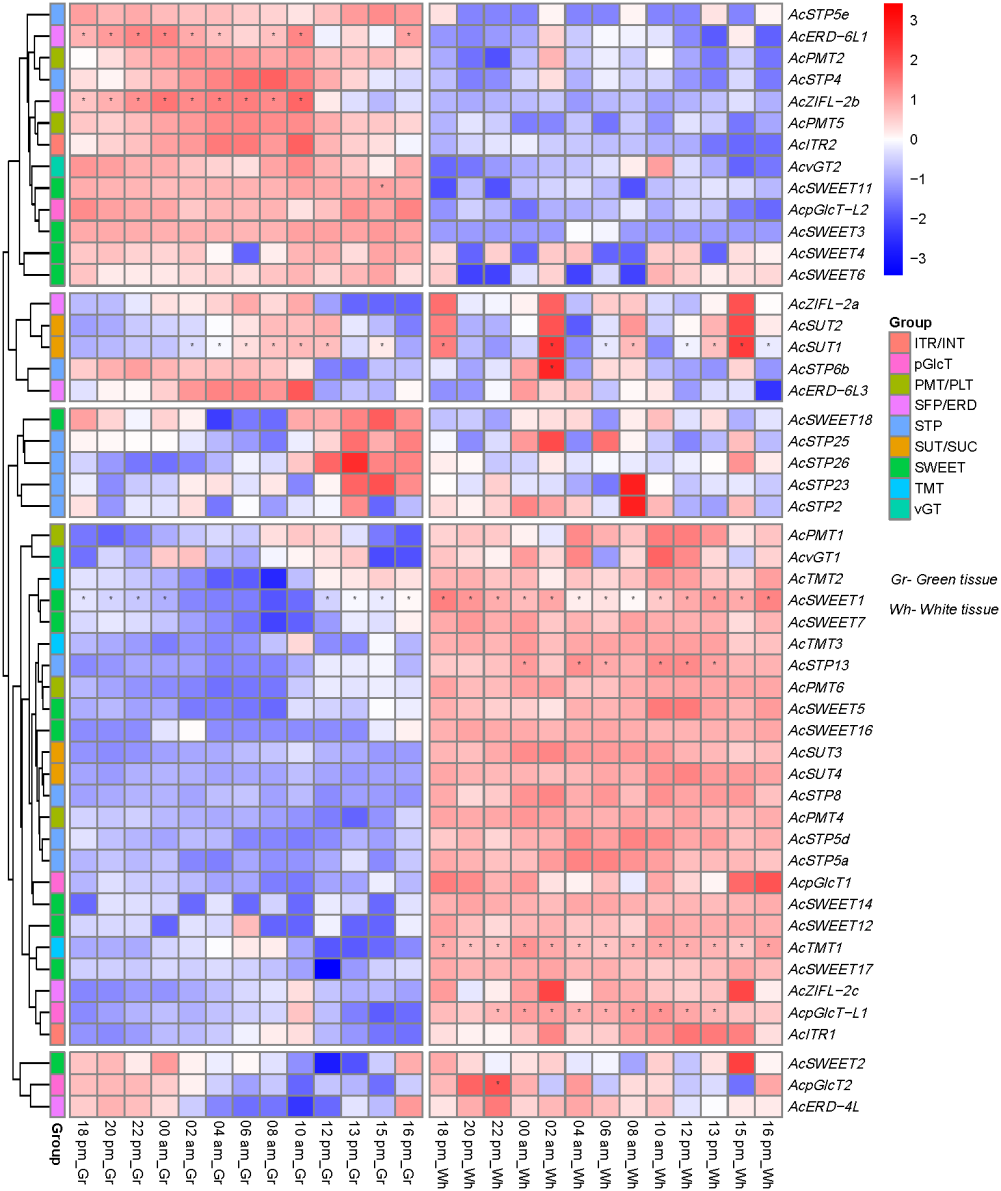
Expression pattern and pineapple sugar transporter genes across circadian rhythm in green (photosynthetic, left panel) tissue at the leaf tip and white (non-photosynthetic, right panel) tissue. The heatmap was created based on the log2 (TPM + 0.01) value of the genes and normalized by row. Red color represents a high transcript abundance, and blue represents low transcript abundance, and asterisks (*) represent the FKM values above 100.

We then investigated the expression patterns of sugar transporter genes in the source and sink leaf segments and their regulation during circadian rhythm using the RNA-seq data obtained from the photosynthetic (green tip) and non-photosynthetic (white base) leaf tissues of field-grown pineapple plants at 2 h intervals over a 24 h period (Ming et al., 2015). Based on the difference in the expression patterns of the source and sink tissues, we could sort out the gene family members contributing to carbon fixation in the pineapple. We found approximately 25% sugar transporters with no or extremely low expression in the selected leaf segments for which data is not included (**Figure 8**). The remaining sugar transporters could be divided into three categories based on the expression patterns. The first group showed high expression in the photosynthetically active leaf segment and consisted of *AcSTP4*/5e, *AcPMT2*/5*, AcERD-6L1, AcZIFL-2b, AcITR2, AcvGT2, AcpGlcT-L2* and *AcSWEET3/4/6/11*. The second group of transporters had expression in both types of leaf segments, which consisted of *AcZIFL−2a, AcERD−6L3, AcERD−4L, AcSUT1/2, AcSTP2/6b23/25/26, AcpGlcT2* and *AcSWEET2/4/6/18* (**Figure 8**). The remaining sugar transporters genes had higher expression in the non-photosynthetic leaf and were represented by *AcSUT3*/4, *AcSTPs* and *AcSWEETs*. These findings suggest that sugar transporter genes play essential roles in sugar loading and unloading in photosynthetic and non-photosynthetic leaf segments to assist carbon fixation in pineapple. Furthermore, the sugar transporter expressions during the day-night rhythm indicated a diel expression of almost all the genes. However, the expression patterns were more dependent on the circadian rhythm in the photosynthetic leaf segment. For instance, *AcSWEET2* and *AcERD*-4L expression levels increased from 16:00 pm to 00:00 am, and *AcSTP23/25/26* and *AcSWEET18* had high expression between 10:00 am -16:00 pm in the green tip. Similarly, the expression of *AcSTP4, AcZIFL−2b, AcPMT5, AcITR2, AcvGT2* increased gradually from 18:00 pm to 10:00 am (**Figure 8**).

### Pineapple sugar transporter genes are regulated under cold stress

**Figure 9:**
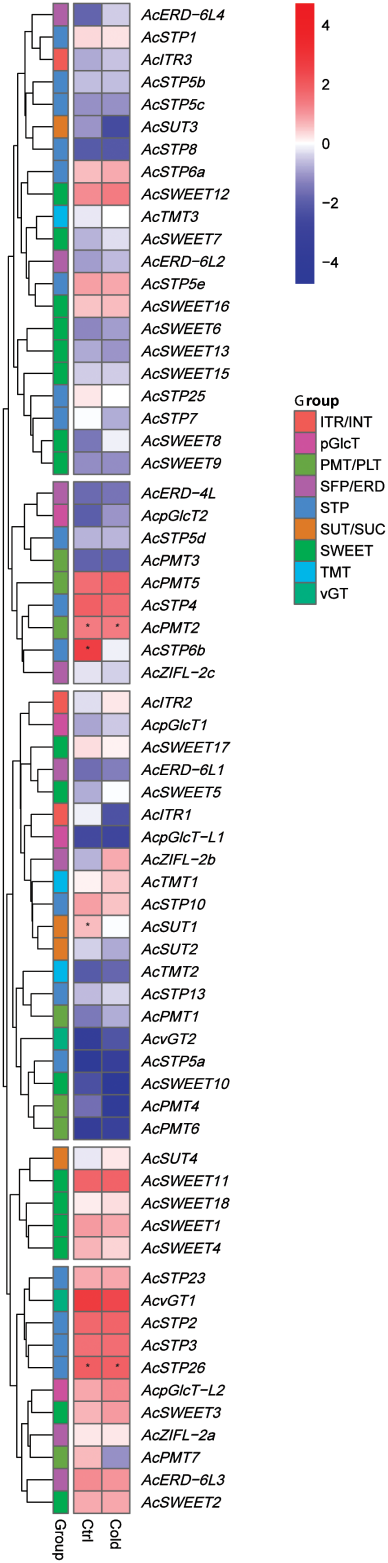
Expression profile of pineapple sugar transporter genes under cold stress (4°C). High and low expression is indicated by red and blue colors, respectively, and asterisks (*) represent the FKM values above 100.

We studied the role of pineapple sugar transporter genes during cold stress response at the molecular level by using RNA-seq data obtained from a cold-tolerant genotype of the pineapple cultivar ‘Shenwan’ (Chen et al., 2016). A heatmap was generated to compare expression levels of sugar transporter genes in control and cold stressed plants (**Figure 9**). The results indicated a differential expression of sugar transporter genes during cold stress. For instance, *AcSTP6b* and *AcSUT1* had a higher expression in control plants compared to the cold-treated plants. However, *AcTMT1*, *AcITR2*, *AcvGT2, AcZIFL−2b*, *AcERD*-6L1 and *AcSWEET5/12* expressions increased after cold treatment. Furthermore, the expression levels of *AcPMT2*, *AcSTP26* and *AcSWEET1*/*11* were high in both conditions and remained unchanged during the cold stress (**Figure 9**). These findings suggest that sugar transporter genes might contribute to the cold tolerance of pineapple.

### Expression analysis of pineapple transporter genes during fruit development by RT-qPCR

**Figure 10:**
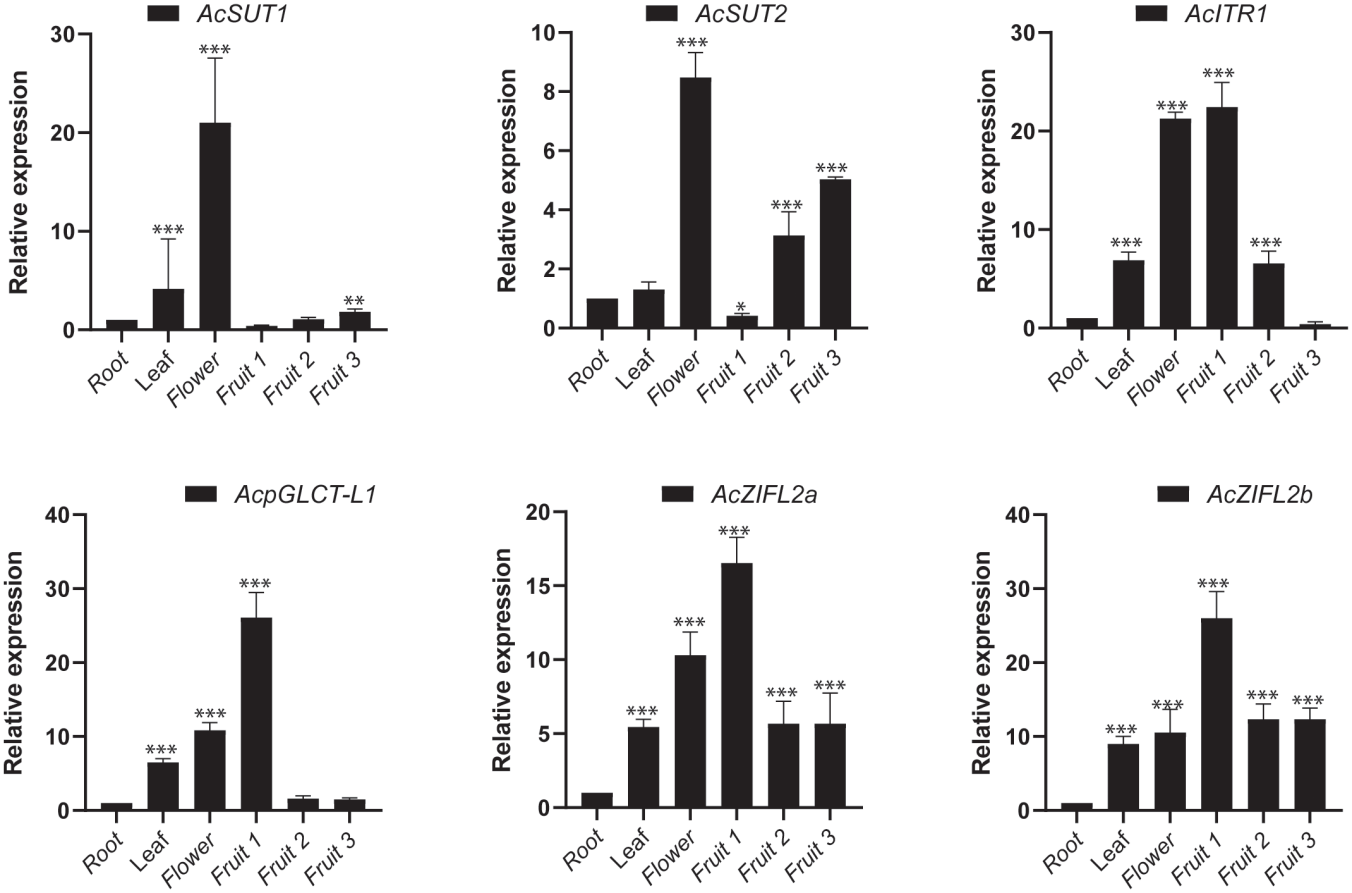
The relative expression levels of 6 sugar transporter genes of pineapple verified by quantitative real-time PCR (RT-qPCR). Gene expression is represented in fold change of expression calculated against pineapple *EF1*α by the Livak method (2^−ΔΔCT^). Vertical bars represent the mean± SE of three biological replicate assays. The asterisks represent statistically significant values (*p < 0.05, **p < 0.01 and *** p < 0.001).

We then investigated the expression patterns of six sugar transporter genes, *AcSUT1*, *AcSUT2*, *AcpGLCT1*, *AcITR1*, *AcZIFL2a* and *AcZIFL2b*, during fruit developmental stages to validate the RNA-seq data and obtain insights into the potential roles of sugar transporter genes in fruit development. The six genes were selected from different subfamilies based on their expression patterns in fruit developmental stages. We conducted RT-qPCR experiments to observe their expression patterns in the root, flower, and fruit stages: early (fruit1), middle (fruit3), and late (fruit7). The results demonstrated that the expression levels of *AcSUT1, AcSUT2*, *AcpGLCT1*, *AcITR1*, *AcZIFL2a* and *AcZIFL2b* were increased in the leaves, flower, and fruit stages: early (fruit1), middle (fruit2), and late (fruit3) compared to the root (**Figure 10**). The expression of *AcITR, AcpGLCT, AcZIFL2a* and *AcZIFL2b* was around 16 to 26 folds higher in fruit1 and further decreased in later stages. For instance, the levels of *ITR* were around 22 times higher in the initial growing stage (fruit1), which dropped with the fruit ripening stage. These sugar transporters also showed higher expression in flowers ranging from an 8 to 21 fold increase in expression compared to roots (**Figure 10**).

### Heterologous expression of selected sugar transporters in yeast

**Figure 11:**
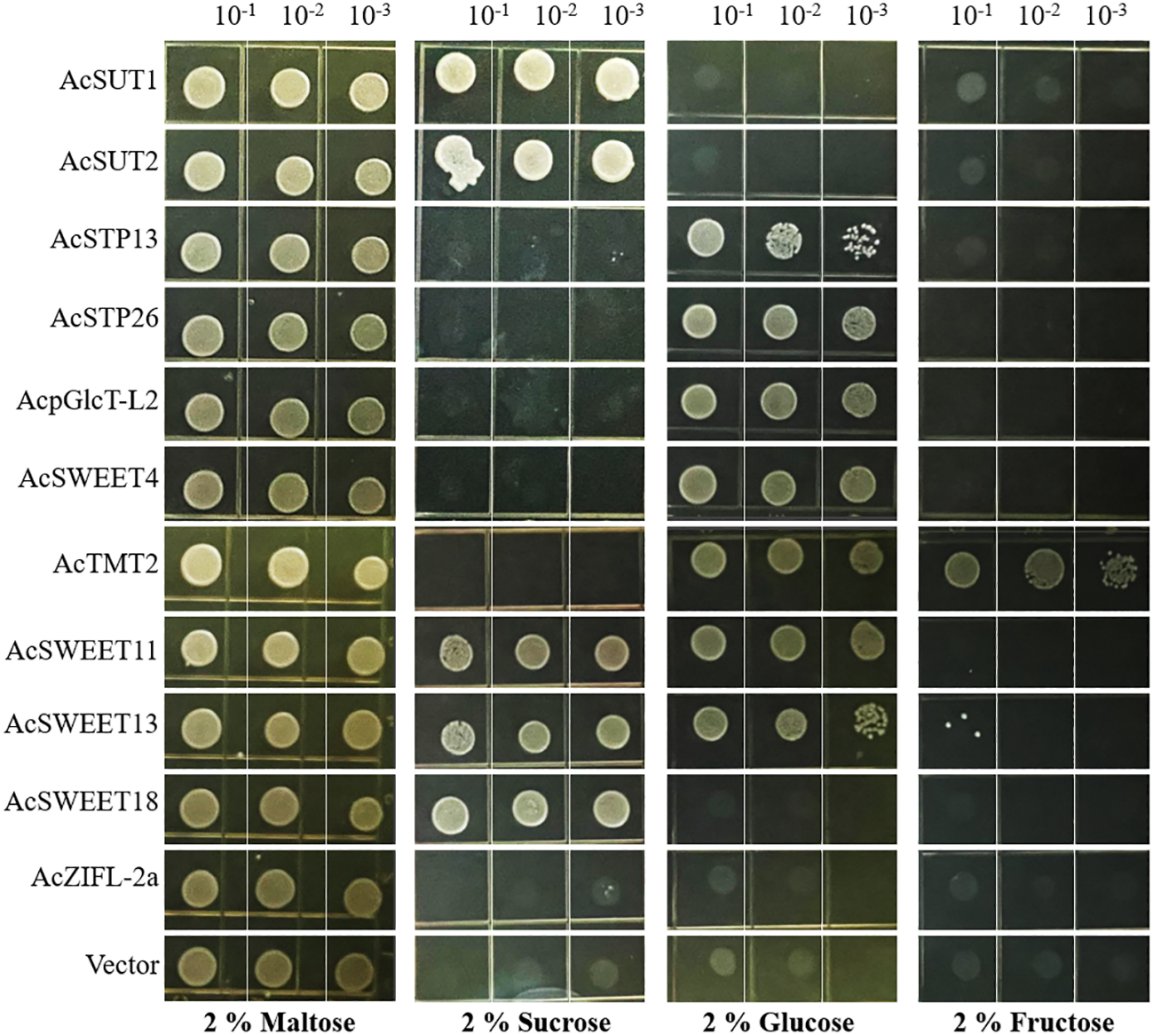
Heterologous expression of representative sugar transporters in the hexose transport-defective yeast strain EBY.VW4000. The yeast cultures harboring respective vectors were incubated until OD_600_ reached 0.2 and then grown in serial dilutions on SD (-Trp) media containing either 2% maltose (positive control), 2% sucrose, 2% glucose or 2% fructose. The plates were incubated at 30°C, and the growth was documented after 3d.

To understand the possible functions of the sugar transporters in pineapple, we expressed representative sugar transporters of different families in the hexose transport-deficient *Saccharomyces cerevisiae* mutant strain EBY.VW4000, which cannot grow on hexose or sucrose, however, it grows well on disaccharide maltose. After transformation, the yeast’s growth ability was examined on selective culture media supplemented with 2% maltose (positive control), 2% sucrose, 2% glucose, and 2% fructose (**Figure 11**). The growth result indicated that AcSUT1 AcSUT2, along with AcSWEET11/13/18, enabled yeast growth in the sucrose medium. AcSTP13, AcSTP26, AcpGlcT-L2, AcTMT2 and AcSWEET4/11/13 had detectable growth on glucose media. AcTMT2 could complement the growth defect of mutant yeast on glucose and fructose enriched media, showing maximum growth on glucose than on fructose. The yeast transformant of AcSWEET11/13 shows preferences for both glucose and sucrose. However, AcSWEET18 was able to complement growth defects on sucrose media only. In comparison, AcZIFL2a and vector control did not show detectable complementation of growth deficiency in any media (**Figure 11**).

## Discussion

In most plants, sugar transporters accomplish carbohydrate allocation that is used as a universal energy currency for various functions. Because of their significance, sugar transporters have been characterized in several plants. However, the information about pineapple sugar transporters remains scarce. In the present study, we have studied pineapple sugar transporters and their features, expression patterns and putative functions in detail.

We initially investigated the gene parameters, structure and phylogenetics of identified sugar transporters. The instability index of most sugar transporter proteins was less than 40, inferring that these are stable membrane proteins, which is also indicated by their higher aliphatic index. The isoelectric point of the sugar transporter proteins in pineapple remains within the neutral range. The isoelectric point defines the pH of their surrounding environment and helps predict the subcellular localization of unknown proteins. While the GRAVY index values measure the soluble nature of proteins, the increasing values indicate greater hydrophobicities. Pineapple sugar transporters are localized in various cellular organelles, including the tonoplast, plasma membranes and chloroplast suggesting their role as a sugar transporter is highly coordinated and wide-ranging (**Table 1**). The location of genes on the same and different chromosomes is a consequence of tandem and segmental duplication events. The sugar transporter genes may have been created and diversified from common ancestors through gene duplication events (Cannon et al., 2004). It can be assumed that the sugar transporter genes from pineapple had undergone segmental and tandem duplications leading to 7 subfamilies and their respective members. A higher degree of tandem duplication was observed among STP members. Similarly, duplicated ERD subfamily arose from segmental duplication within two chromosomes, and only two members were considered unattributed scaffolds (**Figure 1**). Generally, sugar transporters are grouped based on their presence in monocot or/and eudicot species, functional similarities, and location (Braun and Slewinski, 2009). The proteins of a single subfamily with identical motifs suggest that they might have a specific function (**Figure 3**). The phylogenetic analysis indicated that most pineapple sugar transporter subfamilies (e.g., SUT, TMT, and ITRs) share close homology with corresponding members of rice, proposing a monocotyledonous lineage (**Figures 4**–**6**; **Additional Figure 1**).

The development of the floral organs greatly depends on sugars (carbon resource) from sink tissues (e.g., mature leaves). The movement of sugars, typically sucrose, is required for normal flower development through the continuous source to sink flow (phloem unloading). In addition, sepals and juvenile petals can photosynthesize and act as supplementary energy sources (Müller et al., 2010). It is reported that after floral induction, the movement of sugars to the apical meristematic tissues decreases and is diverted towards flowers, mainly through transporters (Gisel et al., 2002). Further increase in sugar transporter gene expression occurs after fertilization (Gahrtz et al., 1996). RNA-seq data showed that members of *AcSWEET, AcZIFL, AcSTP, AcSUT* and *AcpGLCT* expressed significantly in sepals indicating they are involved in sugar -to and -fro movement across cells during photosynthesis (**Figure 7**). Additionally, the opening of a flower is essential for successful pollination and involves changes in the cellular environment. Carbohydrates get accumulated in young petals and subsequently degraded, generating osmotic potential through water influx at the time of anthesis (van Doorn and Kamdee, 2014). Higher expression of *SUT* and *STP* members suggested their role in sugar sequestration during petal development in pineapple. Besides, in mature petals, photosynthetic machinery deteriorates and accumulates secondary metabolites like polyols (sugar alcohols) that attract pollinators (Wagstaff et al., 2009). The stigma also secretes sugar-based compounds that act as attractants to pollinators and provide adhesion and nutritious supplement for the germination of developing pollens (Lin et al., 2014). Vacuolar sugar transporters like *AcvGT*, *AcTMT* and *AcPMT* were abundant during gynoecium development. The expression pattern of *AcSWEET4* in gynoecium, ovary and fruit suggests that it could be a source of sugar for developing embryos (**Figure 7**). More than half of the sugar transporter genes had higher expression in the stamens of the pineapple flower. Filaments in stamens help in the movement of sugars for unloading into sink tissues (anthers) and the soluble sugar is stored in the nutritive layer (tapetum) for pollen development (Goldberg et al., 1993). In Arabidopsis, *SWEET8* and *SWEET13* show anther specific expression and are involved in the microspore development (Guan et al., 2008; Sun et al., 2013). Besides, the sucrose transporter, *AtSUC1*, is highly expressed in mature pollen and mutation in *suc1* produces defective pollen (Sivitz et al., 2008). Similarly, *SWEETs*, *SUT* and *STPs* were predominantly expressed in the pineapple stamen. *AcSWEET6/8/9/12/13/15* and *SUT1/2* were highly expressed in stamen tissues, suggesting that the SWEETs and SUT play a major role in directing the sugars in pineapple anther tissues.

Sucrose carriers have a tissue-specific location, for example, AtSUC1/2/3 in parenchymatous xylem tissues and companion cells, pollen, root tip and guard cells, AtSUC5 in the endosperm, AtSUC8/9 in floral organs (Stadler et al., 1999; Meyer et al., 2000; Meyer et al., 2004; Sauer et al., 2004; Sivitz et al., 2007; Baud et al., 2008). In pineapple, *SUT1/4* along with *SWEET3/4/13/18* were highly expressed in fruit developmental stages, indicating that they may be involved in the storage and distribution of sugars in pineapple fruit. Sucrose present in the apoplastic space may be transported through AcSUT1 to fruit parenchyma cells (**Figure 7**). Consistently, SUT1/2 carrying yeast mutant EBY.VW4000 grew well on the sucrose medium only, indicating their substrate specificity (**Figure 11**). A fraction of sucrose in apoplasm is hydrolyzed to glucose and fructose by the cell wall invertases, which is subsequently transported into phloem and fruit parenchyma cells by hexose transporters (STP) members. In *Arabidopsis*, STP10 (Rottmann et al., 2016) and STP13 (Norholm et al., 2006) are high affinity transporters involved in the uptake of hexose sugars. We found that the pineapple STP13/26 enabled the yeast mutant EBY.VW4000 to grow well with glucose. Therefore, it can be speculated that pineapple AcSTP13 and AcSTP26 might be high affinity transporters and are possibly involved in the energy supply to the dividing and growing cells in the fruit. Subsequent fall in their activity during the ripening process could be due to high levels of monosaccharide accumulation in the cells. However, hexose transporters like *AcpGlcT-L2* and *AcTMT2* exhibited a rapid increase in expression during fruit ripening. Heterologous expression in defective yeast indicated that AcpGlcT-L2 transports glucose, whereas AcTMT2 could transport glucose and fructose, indicating that they may play a role in unloading in the sink.

Several transporters have broad substrate specificity, allowing them to modulate their transport activity by expressing at specific developmental stages or environmental changes. In grapes, VvSWEET7 can transport glucose and sucrose, which is involved in sugar partitioning during different fruit developmental stages (Breia et al., 2020). Similar transport activity is shown by AtSWEET13 (Han et al., 2017), which is expressed during anther development (Isoda et al., 2022). Consistently, pineapple SWEET4/11/13/18 enabled yeast mutant EBY.VW4000 to survive on a medium with either sucrose (AcSWEET18) or glucose (AcSWEET4), or both (AcSWEET11/13), indicating a broad specificity for pineapple SWEETs (**Figure 11**). The dual substrate-specific *SWEET* genes (*AcSWEET11* and *AcSWEET13*) in pineapple were abundant during the initial and ripening stages of fruit. These transporters may contribute to the movement of glucose in the unripe fruit, which later in development could switch their activity by permitting sucrose accumulation in the fruit vacuoles. Similar glucose and sucrose transport steps can be carried out separately by AcSWEET4 and AcSWEET18, respectively. Previously, *AnmSWEET5* and *AnmSWEET11*, two of the pineapple SWEETs proteins, exhibited a negative correlation with sucrose levels during the development of the pineapple fruit, demonstrating their roles in the regulation of sucrose levels during fruit development (Guo et al., 2018). Consistent with the previous results, *AcSWEET5/6/11* showed a negative correlation with fruit developmental stages; however, *AcSWEET11* expression returned to a maximum level at the Fr_S7 stage. Taken together, these findings suggest that sugar transporters play crucial roles in the reproductive development of pineapple.

It is proposed that sucrose is transported into the vacuole during the daytime, followed by breakdown into simpler sugars (e.g., glucose and fructose) and sugar transporters mediated uptake into sink cells for adequate storage (McRae et al., 2002). The isolated pineapple vacuoles mainly contain fructose and glucose, but whole-leaf extracts contain significant sucrose levels (Kenyon et al., 1985; Christopher and Holtum, 1998). CAM plants such as pineapple must store enough sugars in the vacuole during the day to meet PEPC substrate requirements of the night. However, they must also continue to export sugars to keep sink tissues growing (Borland and Dodd, 2002). The green photosynthetically active leaf segments execute CAM and play a vital function in regulating carbon metabolism across the circadian rhythm. Previously, it was hypothesized that AtERD6 homologs have a role in sugar transport (Buttner, 2007). The two ERD members (AcERD−6L1 and AcZIFL-2b) could be helping in the transport of the sugar at night to provide substrates for CO_2_ uptake at night. Similarly, the vacuolar expressed the AcSTP4 and AcSTP5e could be assisting in sugar loading in a circadian-dependent manner. In *Arabidopsis*, SWEET11/12 and SUC2 carry out the apoplasmic phloem loading, and *sweet11/12* double mutant displayed significant defects in the sugar loading and accumulated sugars in the leaves (Chen et al., 2012). In the present study, *AcSWEET3/4/6/11* had a high expression in the photosynthetically active leaf segment, indicating that they could be involved in the glucose movement during the deacidification step in CAM photosynthesis. Moreover, the sucrose transporters *SUT1*/2 showed a circadian-dependent expression in photosynthetic tissue and could be involved in the loading of sucrose into the phloem. During the night, glucose and fructose could be transported to the apoplasm through AcSTP23/25/26 to supply substrates in the glycolysis pathway. In contrast, *AcSUT3*/4 were expressed in non-photosynthetic tissue, indicating they contribute to phloem unloading along with *AcSWEET1/5/7/12/14/16/17* (**Figure 8**).

The pineapple plant shows some tolerance towards drought due to its modified C3 type photosynthetic pathway called Crassulacean Acid Metabolism (CAM) (Ming et al., 2015). However, it is a cold-sensitive plant and almost all pineapple varieties show injury after cold exposure. The cold stress severely affects pineapple production leading to low-quality fruit and revenue loss (Chen et al., 2016). The pineapple fruit development is significantly hampered below 10°C, a significant cause for the consequential crop loss. Understanding how plants have evolved to survive cold stress is vital for engineering cold tolerance in pineapple (Lobo and Siddiq, 2017). It is well documented that sugars act as compatible osmolytes and prevent cell damage during cold stresses. Therefore, it is crucial to comprehend the role of sugar transporters during cold stress as several sugar transporter genes differentially express and regulate the osmolarity of the cell during cold stress (Fowler and Thomashow, 2002). The decline in fruit quality by an unbalanced acid: sugar ratio negatively impacts its economic value. It can be presumed that manipulated sugar transport may alleviate the damage to the crop during cold stress conditions making it possible to regulate the expression of these transporters to achieve higher sugar levels (Wingenter et al., 2010).

## Conclusion

Due to extreme temperature conditions in tropical climates, CAM plants such as pineapple developed a highly efficient spatiotemporal sugar partitioning, assimilation and sequestration system. As a result, the pineapple plant utilizes a significantly higher number of soluble sugar molecules with varying degrees of loading in the day and night cycles. With the exploitation of the phylogenetic link between pineapple, rice and *Arabidopsis*, it is possible to identify the role of sugar transporters in sugar allocation during fruit developmental stages of the agronomically important pineapple crop. It should also be noted that in pineapple, several sugar transporter subfamilies modulate their expression in photosynthetic tissue and also show a circadian rhythm-dependent expression pattern. Additionally, comparative expression analysis in cold tolerant and susceptible pineapple varieties indicated differential regulation of sugar transporters during cold stress, suggesting that they might mitigate the cold-sensitive phenotype. Heterologous expression in defective yeast indicated that the pineapple plant utilizes different sugar transporters to transport a range of substrates. The information provided in this study will further enhance our understanding of how sugars metabolize in the pineapple and help future research aiming to characterize and establish the physiological roles of these sugar transporter genes in pineapple.

## Data availability statement

Publicly available datasets were analyzed in this study. This data can be found here: https://www.ncbi.nlm.nih.gov/bioproject/?term=PRJNA331052 https://www.ncbi.nlm.nih.gov/bioproject/?term=PRJNA305042 https://www.ebi.ac.uk/ena/browser/view/PRJEB38680?show=reads.

## Author contributions

BF: Investigation, Data curation, Validation, Writing – original draft. BJ: Investigation JG: Data curation. LW: Data curation. XN: Data curation, Validation. YC: Validation. PZ: Methodology. MA: Conceptualization, Investigation, Methodology, Data curation, Supervision, Validation, Writing – original draft, Writing - review & editing. YQ: Conceptualization, Funding acquisition, Project administration, Supervision. XW: Funding acquisition. All authors have read and approved the final version of the manuscript. All authors contributed to the article and approved the submitted version.

## Funding

This work was supported by the Guangxi Distinguished Experts Fellowship to YQ, Science and technology innovation project of Pingtan Science and Technology Research Institute (PT2021007, PT2021003), the Science and Technology Major Project of Guangxi (Gui Ke AA22068096), Project of Guangxi Featured Fruit Innovation Team on Pineapple Breeding and Cultivation Post under National Modern Agricultural Industry Technology System (nycytxgxcxtd-17-05). Guangxi Academy of Agricultural Sciences basic Research Project (Gui Nong Ke 2021YT046). The funding bodies played no role in the design of the study and collection, analysis, and interpretation of data and in writing the manuscript.

## Acknowledgments

We thank all members of the Qin lab for their assistance in the experiments. We especially thank Dr. Binghua Wu (Fujian Agriculture and Forestry University, China) for kindly providing the yeast mutant strain EBY.VW4000.

## Conflict of interest

The authors declare that the research was conducted in the absence of any commercial or financial relationships that could be construed as a potential conflict of interest.

## Publisher’s note

All claims expressed in this article are solely those of the authors and do not necessarily represent those of their affiliated organizations, or those of the publisher, the editors and the reviewers. Any product that may be evaluated in this article, or claim that may be made by its manufacturer, is not guaranteed or endorsed by the publisher.

## Supplementary material

Additional Figure 1 Phylogenetic analysis of the early response dehydration 6**/**sugar facilitator protein family (ERD-6).

Additional File S1 List of the sequences used in the study.

Additional File S2 List of primers used for expression analysis.
