# Supplementary figures and images for: Identification and expression analysis of pineapple sugar transporters reveal their role in the development and environmental response
Source: Front Plant Sci. 2022 Oct 24;13:964897. doi: 10.3389/fpls.2022.964897 (PMC9638087; doi:10.3389/fpls.2022.964897)

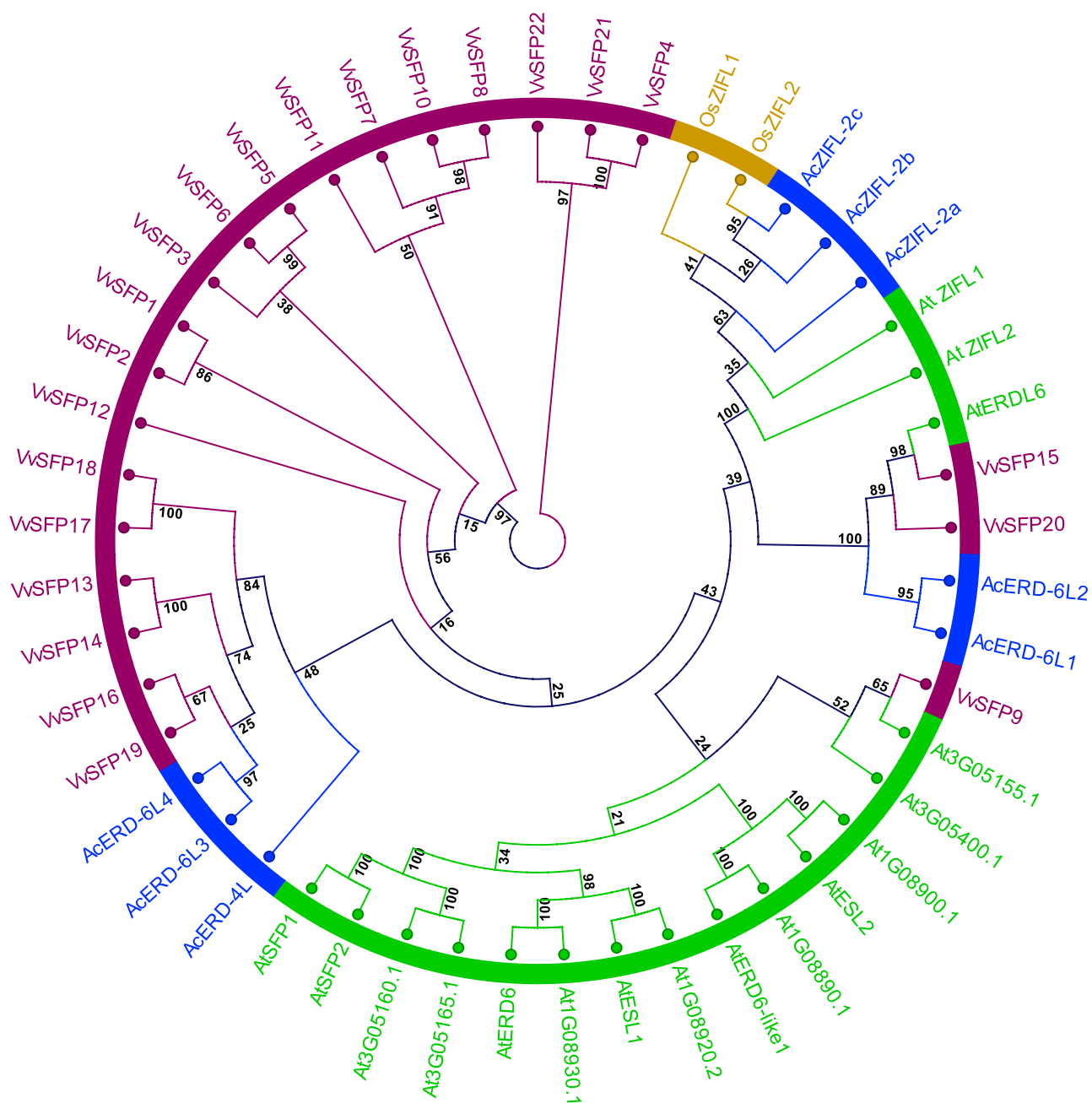

● *Vitis vinifera*
● *Arabidopsis*
● *Oryza sativa*
● *Ananas comosus*

Supplement: Additional Figure 1 — Phylogenetic analysis of the early response dehydration 6/sugar facilitator protein family (ERD-6). [file Image_1.pdf]
