# Supplementary material for: Identification and expression analysis of pineapple sugar transporters reveal their role in the development and environmental response
Source: Front Plant Sci. 2022 Oct 24;13:964897. doi: 10.3389/fpls.2022.964897 (PMC9638087; doi:10.3389/fpls.2022.964897)
Supplement: Additional File S1 — List of the sequences used in the study. [file Table_1.docx]

>AcSUT1 Aco004135.1

MEHKGGGGGGEVELAGTSGPGAVQRGNAGAEAPAPAPATKPLSLPRLILACMVAGGVQYGWALQLSLLTPYVQTLGLSHALSSLCALRPDCSCSSALCWFMERQMPFAARTAEPFILVIVIGFSSDIGYALGDTKEHCSVYRGPRWHAAIVYVLGFWLLDFSNNAVQGPARALMADLSVGHFGPNAANAIFCSWMAFGNILGYSSGSSGKWHEWFPFLQTAACCEACANLKGAFFIAVILLIFSLSVTMIFAREAPLPPRNSGKADEEDGDEERMNFFTMFKAFKNLPPGMVSVLFVTGLTWLSWFPFILYDTDWMGREVYHGDPKGSPAEVAAYDRGVREGAFGLLLNSIVLGVSSFLIEPLCRKFTAKFVWVISNFIVFAAMAATAIISVWSLNDYRGSIQQAITADKDVRTAALVLFAALGFPLAVLFSVPFAVTAQIAANGGGGQGLCTGVLNISIVVPQVIVALGAGPWDALFGKGNLPAFALASAIALVSGFVGFFMLPKLSKSNFKTAGIVGGH

>AcSUT2 Aco004131.1

MDHKGGGGAGGELELAGTRGPGAVERGNAAPATKPLSLAKLILACMVAGGVQYGWALQLSLLTPYVQTLGLSHALSSIMWLCGPIAGFVVQPCVGLWSDRCRSPLGRRRPFILVGCVLICCAVIVIGYSSDIGYALGDTKEHCSVYRGPRWHAAIVYVLGFWLLDFSNNAVQGPARALMADLSVGHFGPNAANAIFCSWMAFGNILGYSSGSSGKWHEWFPFLQTAACCEACANLKGAFFIAVILLIFSMSVTMIFAREVPLPPRNSGKADEEDGDGERMNFFAVFKAFKNLPPGMVSVLLVTGLTWLSWFPFILYDTDWMGREVYHGDPKGSPAEVAAYDRGVREGAFGLLLNSIILGLSSFLIEPLCRKFTAKLVWVISNFILFAAMAATAIISVWSLNDYRGSIQHAITADKDVRIAALVLFAALGFPLAVLFSVPFAVTAQIAANGGGGQGLCTGVLNISIVVPQVIVALGAGPWDALFGKGNIPAFALASAFSLVSAFVGFFMLPKLSKSNFKTAGVVGGH

>AcSUT3 Aco009281.1

MDGAAASAAATASIRVPYRHLNEAELELVRLDGEPNGEAEVAPSRAVHVPSSSPSSSSSRSAPAMAKPRSSVKTLVLSCTVAAGVQFGWALQLSLLTPYIQTLGIQHAFSSFIWLCGPITGFVVQPCVGIWSDKCTSKYGRRRPFIFVGCLMISVAVTLIGFSADIGYLLGDTSEHCSTYKGTRYRAAVFFIIGFWMLDLANNTVQGPARALLADLSGPDQCSSANAIFCSWMAVGNILGFSSGASGHWHRYLALSLSLSLAHVMCVGVYNLCC

>AcSUT4 Aco000269.1

MTVPGPQGSRGAAAAAAGAGPRRGAAGAGAGTGVRVKVRVPLRRVARAAAVACGVQFGWALQLSLLTPYVQELGVAHAWASLVWLCGPLSGLLVQPLVGHLSDRLGRRRSPFIVGGAATIAFAVITVGFSADIGLALGDPPAATAAPRPRAVAVYVVGFWLLDVGNNATQGPTRALLADLTGKDQRRNRIANAYFSLFMALGNVLGFATGSYSGWFTIFPFTVTSACTINCANLKSAFLLHVILLAITTYISISSIQGVTLPEEEEQQNHSEEAFLWELMGAFRYLTWPVWIVLIVTALTWIGWFPFFLFDTDWMGREIYRGKPDEGQNYHSGVRMGAFGLMLNSVVLGFSSVMLERLCKKWGSGLVWGVANIIMSLCFLAMLVISLVAKSIDYQPSGLPPDGIVVAALIVFAILGAPLAVSKMLFLHQPHCVQESCFLHLVQHASCALPSTNAVHLIPTSIFTIQSVAAFSSYPSYFYIQIVSIVWDGQAYGRRCVAQVVHWITYSIPYAMVTTRLAMGILNLAIVIPQVIVSLGSGPWDQLFGGGNLPAFAVATAAAFLSGLVAIIGIPRSHITRARGHR

>AcSTP1 Aco024297.1

MPAIVVSSGNGPPKEFEGKITVYVVICGIIAATGGLMFGYDIGISGGVTSMDDFLEEFFPAVYVKKHGAKEDNYCKYDNQDLQLFTSSLYLAALVSSFFASKSCTKFGRKLTMQAASVFFLVGVILNAAARDLAMLIIGRILLGVGIGFGNQAVPLFLSEIAPVQIRGALNILFQLDVTIGILIANLVNYFTTNMHPWGWRLSLGLAGVPAVILFFGSLLITETPTSLIERKKLDEGLAMLKKIRGTDNVDAEYDEIVYACEMAQKVKSPFKNLMKRSSRPQLVIAIFMQVFQQFTGINAIMFYAPVLFQTMGFKNDGSLLSAVITGTVNVLSTVVSIVLVDKVGRRMLLLEACVQMFITQVRTATLTSPPSITTIGVILQLYLKTNNNLSPQLAIVVVVMVCLYVMSFAWSWGPLGWLIPSETFPLETRTAGFFFAVSSNMLFTFIIAQSFLSMMCHMRAGIFFFFAGWILIMALFAMFLLPETKNVPIDEMTDRVWKQHWYWKSYMDGEESDSKNKIEDGVDK

>AcSTP2 Aco026106.1

MAGGAIVSAAGGKEYPGTLTLYVFLTCVVGATGGLIFGYDLGISGGVTGMDPFLEKFFPSVYRKKQDESTNQYCKFDSQLLTAFTSSLYLAALIASFFASSVTRMFGRKWSMLGGGFIFLIGAALNGAAQDIAMLIIGRILLGVGVGFANQSVPLYLSEMAPARLRGMLNIGFQLMITIGIFCANLINYGSNKIKAGWGWRLSLALAAVPAAIITIGSLFLPDTPNSLIERGHPDEARAMLRRIRGTDDITEEYNDLVVASEESRRVKHPWVNILQRKYRPQLTMAICIPFFQQLTGINVITFYAPVLFQTLGFSGNAALMSAVITGLVNFIATFLSTFTVDRLGRRMLFLEGGFQMTVCQIIVGTLIAIKFGTSGEGHISKGYGIIVVLFICIYDAGFAWSWGPLGWLVPSEIFPLEIRSAGQSINVSVNMLFTFVIAQAFLTMLCHMKFGLFYFFAGWVVIMTIFIALFLPETKNVPIEEMVIVWKEHWFWGRFINDEDIHVGVEMISGKSKDANVA

>AcSTP3 Aco026103.1

MAGGAIVRAAGAKEYPGTLTLFVFLTCVVAATGGLIFGYDIGISGGVTSMDPFLKKFFPSVYRKKQDESTNQYCTFDSQLLTGFTSSLYLAALIASFFASSVTRLFGRKWSMLGGGFIFLIGAALNGAAQDIAMLIVGRILLGIGVGFANQSVPLYLSEMAPARLRGMLNIGFQLMINIGILCANLTNYGSNKIKAGWGWRLSLALAAVPAAIITVGSLFLPDTPNSLIERGHPDEAKAMLRRIRGTDDVAKEYDDLVAASEESRHVKHPWANILQRKYRPQLTMAICIPFFQQVTGINVIMFYAPVLFKALGFGGDASLMSAVITGLVLLISTFLSTFPVDRLGRRILFLQGGFQMTICQIIVGTLIAMKYGTSGEGRIPKGYGVIVVLFICIYVAGFAWSWGPLGWLVPSEIFPLEIRSAGQSITVSVNMLFTFAIAQAFLPMLCHMKFGIFYFFAGWVVIMTIFVALFLPETKSVPIEEMVLVWKEHWFWSRFVADKDVRIGVEMTNARSKDTN

>AcSTP4 Aco021163.1

MPAGGFSVAAPAGTEFEAKITPIVIISCIMAATGGLMFGYDVGISGGVTSMDDFLKKFFPTVYRQKHEDKESNYCKYNNQDLQLFTSSLYLAGLTATFFASYTTRNLGRRLTMLIAGIFFLVGVVFNAAAQDLAMLIVGRILLGCGVGFANQAVPLFLSEVAPTRIRGGLNILFQLNVTIGILFANLVNYGTSKINGWGWRLSLGLAGIPAVLLTVGSLLVTDTPNSLIERGRLEEGKAVLRRIRGTPNIEPEFEEILAASRAAQRVKHPFRNLLQRRNRPQLRVKHPFRNLLQRRNRPQLVNTLGFKSDAALYSAVITGAVNVLSTLVSIYSVDRIGRRMLLLEAGVQIRGYAVLVVVMVCTFVSSFAWSWGPLGWLIPSETFPLETRSAGQSVTVCANLLFTFVIAQAFLSMLCSLKYGIFAFFSAWVVVMSLFVLFLLPETKNVPIEEMTERVWKQHWFWKRFMDDDADYGVEDGDGDYVKKPSSYNNNASNGSNGVRP

>AcSTP5a Aco028869.1

MVAKHVIEQEKGVKRENIRKAIAFLAETGDLLKIEDILPFFPDFVLIDDFKEEICKSLKDYNNQIEKLKQEMNDATRGADNIRSDISALAQRYAVIDREEECGVCKRKILTAGALQQIGRGYTSMASMAPFYIFPCGHAFHAQCLIAHVTRCASQSQAEYILNLQKQLSLLVSKAQKGSGVSGHDEPVTSTTSIDKLRSQLDDAVASECPFCGDLMIREISLPFIAPEEAEQMTSWEIRPQNKNRTGDILKLEVGVGIVPLECVARTPCASLSKIITSSSFSSLLGFPLTCCCAPLLILQKLEEIRYSEPMAGGVVVSASGGKEYPGKMTLFVFLTCFVASSGGLIFGYDLGISGGVTSMDSFLLKFFPSVYHKEKEDVSTNQYCKFDSELLTLFTSSLYLAALVASFCAATVTRVFGRKWSMFGGGITFLVGAVINGAAESVFMLILGRILLGIGVGFANQSVPLYLSEMAPPKLRGMLNIGFQLMITIGILAANLINYATAKIAGGWGWRVSLGLAAVPAVVIALGALLLPDTPNSLIERGHAEQARSMLSKIRGTDDVQEEYDDLVAASEESKTIKHPWKNILEKKYRPALTMAILIPFFQQLTGINVIMFYAPVLFKTIGFEDDAALMSAVITGLVNMFATFVSIATVDRVGRRALFLQGGTQMLICQIVVGTLIGFQFGTSGVGANLSRGYASCVVLFICVYVAGFAWSWGPLGWLVPSEIFPLEIRSAGQSINVSVNMFFTFIIGQAFLTLLCRLKFGLFYFFGGWVLVMTIFIGLFLPETKNVPIEEMVLAWKKHWFWGKFISDDDIHVGKKTSTFVVSHHYTVVEVETIITMI

>AcSTP5b Aco007397.1

MAGGVVVSASGGKEYPGKMTLFVFLTCFVASSGGLIFGYDLGISGGVTSMDSFLLKFFPSVYHKEKEDVSTNQYCKFDSELLTLFTSSLYLAALVASFFAATVTRVFGRKWSMFGGGITFLVGAVVNGAAESVFMLILGRILLGIGVGFANQSVPLYLSEMAPPKLRGMLNIGFQLMITIGILAANLINYATAKIAGGWGWRVSLGLAAVPAVVIALGALLLPDTPNSLIERGHAEQARSMLSKIRGTDDVQEEYDDLVAASEESKTIKHPWKNILEKKYRPALTMAILIPFFQQLTGINVIMFYAPVLFKTIGFEDDAALMSAVITGLVNMFATFVSIATVDRVGRRALFLQGGTQMLICQIVVGTLIGFQFGTSGVGANLSRGYASCVVLFICVYVAGFAWSWGPLGWLVPSEIFPLEIRSAGQSINVSVNMFFTFIIGQAFLTLLCRLKFGLFYFFGGWVLVMTIFIGLFLPETKNVPIEEMVLAWKKHWFWGKFISDDDIHVGKKTSTFGA

>AcSTP5c Aco027220.1

MAGGVVVSASGGKEYPGKMTLFVFLTCFVASSGGLIFGYDLGISGGVTSMDSFLLKFFPSVYHKEKEDVSTNQYCKFDSELLTLFTSSLYLAALVASFFAATVTRVFGRKWSMFGGGITFLAGAVINGAAESVFMLILGRILLGIGVGFANQSVPLYLSEMAPPKLRGMLNIGFQLMITIGILAANLINYATAKIAGGWGWRVSLGLAAVPAVVIALGALLLPDTPNSLIERGHAEQARSMLSKIRGTDDVQEEYDDLVAASEESKTIKHPWKNILEKKYRPALTMAILIPFFQQLTGINVIMFYAPVLFKTIGFEDDAALMSAVITGLVNMFATFVSIATVDRVGRRALFLQGGTQMLICQIVVGTLIGFQFGTSGVGANLSRGYASCVVLFICVYVAGFAWSWGPLGWLVPSEIFPLEIRSAGQSINVSVNMFFTFIIGQAFLTLLCRLKFGLFYFFGGWVLVMTIFIGLFLPETKNVPIEEMVLAWKKHWFWGKFISDDDIHVGKKTSTFGA

>AcSTP6a Aco014415.1

MAGAVIVSNGGGGRDYPGNLTLYVLFTCFVAATGGLIFGYDLGISGGVTSMDPFLMKFFPSVYRQEKADESANQYCKFDSQLLTAFTSSLYLAALIASFFASSVTRVFGRKWSMLGGGVTFLAGAAVNGAARNVLMLILGRILLGVGVGFANQSVPLYLSEMAPARLRGMLNIGFQLMITIGILCANLINYGTAKIKGGWGWRISLALAAIPGAIIAVGSLFLPDTPNSLIERGHADRAKRMLRRIRGTDDILDEFNDLIAASEESKLVKHPWANIIKRKYRPQLTMAVLIPAFQQLTGINVITFYAPVLFKTIGFGGDASLMSSVITGLVNAFATLVSIATVDKVGRRKLFLQGGAQMLICQVIVGTLIAKKFGLSGEAEEGLSRGYAAVVVLFICLYVAGFAWSWGPLGWLVPSEIFPLEIRSAGQSINVSVNMLFTFGVAQAFLAMLCHLKYFLFFFFAAWVVLMTAFIALFLPETKNVPIEEMLLVWRAHWFWGHFLAHDDDVHVAAANSNLELGGANNAKAKQGGTAVAS

>AcSTP6b Aco009133.1

MAGGAFVNPAGGKDYPGKLTLFVFLTCVVAATGGLIFGYDIGISGGVTSMDSFLQKFFPSVYDKEQRDKSTNQYCKFDSQLLTTFTSSLYLAALIASFFASVVTRVFGRKWSMFGGGITFLIGAALNGAARNVLMLILGRILLGIGVGFANQSVPVYLSEMAPARLRGMLNIGFQLMITIGILCANLINYGTAKIKGGWGWRVSLALAAVPAGIITVGSLFLPDTPNSLIERGHDDEARRMLRRIRGTDDVDREYNDLVAASEESKAVKHPWANIVKRKYRPQLTMAILIPFFQQLTGINVIMFYAPVLFKTIGFGGDASLMSAVITGLVNVFATFVSIFTVDKLGRRKLFLQGGSQMLACQVIVGTLIAIKFGTSGEAHLSKTYAAFVVFFICLYVAGFAWSWGPLGWLVPSEIFPLEIRSAGQSINVSVNMLFTFVIAQAFLAMLCHMKFGLFYFFGGWVVVMTTFVALFLPETKNVPIEEMILVWRSHWFWGNFIADTDIHVGSTLEMANGDKSKSAA

>AcSTP7 Aco007400.1

MAGGGFTGPPGSKDYPGKMTGYVFLACLVASSGGLIFGYDIGISGGVTSMDSFLTKFFPEVYRKEQRATGSNQYCEFNSQLLTTFTSSLYLAALVACFFASTITRVFGRKLSMLGGGLIFMVGAIINGAARNVAMLIVGRILLGLGIGFTNQSVPLYLSEMAPPRHRGMLNIGFQLMITIGIFIANLINYGTSKIKGGWGWRVGLGLAVVPALIMTVGSLFLPDTPNSLIDRGHKDEARTMLRRVRGTDDIAAEFEDLVQASEASQAVKHPWSALMRRRYRPQFIMSFMIGGLQQLTGINVVMFYAPVLFKTLGFGAEASLMSAVITGLINVFSTLVSVFTVDRIGRRVLFLQGSVQMTASQVVVGALIGAKFGTDGIGTISRGYAILVVVFVCLFVSAFAWSWGPLGWLVPSEIYPLEIRSAAQSITVSVNMLFTFLVAQIFLPMLCAFKFGLFFFFAAWNIINFLYIAVLLPETKGIPIEEMSRIWKQHFIWSRYVTDDSDAKSNNDAARNVDITTRSRTDSV

>AcSTP8 Aco008491.1

MAGGFSVPDGGKEYPGKMTLFVFLACLVASSGGLIFGYDIGISGGVTSMDPFLLKFFPDVYHEEKLQKSTSQYCKFNSQLLTTFTSSLYLAALVASFFASTVTRLLGRKPSMFCGGLVFLAGSVINGAAKDVAMLIIGRIFLGIGIGFASQSVPLYLSEMAPARLRGMLNIGFQQMITVGILIANIINYGTAKIEGGWGWRVSLGLGAVPAIIMTAGSLLLPDTPNSLIERGHTDEAKSMLCRIRGTEDVQAEFVDLVAASEASKAVKHPWSTLRQRSYRPQLVMSLLIPAFQQLTGINVINFYAPVLFKTIGFGSEASLMSAVIIGFFKVASCLISIFVVDKLGRRPLLIEGGLQMIFFQWEQNVKRIDQYRGRSSYTFVPMSASSIDAYEKVVVGILIGIKFGSDGVGTISKPYAIGVVSCICFFVSAFDWSWGPLGWLVPSEIYPLEVRSAGQSITVSVNMLFTFIVAQTFLPMLCHFKFGLFFFFAACETIMTVYVTLFLPETKNIPIEEMDRIWKKHWYWSRFVTDVPGGDHEMTKPKSSV

>AcSTP10 Aco014002.1

MAGGGFVEHGEVKDYSGKVTAFVVITCLVAATGGLIFGYDIGISGGVTSMDVFLEEFFPSVYHDEKSAVGGKSEWCQFDSQLLTAFTSSLYIAGLLATYVASAVTRKSGRRISMLIGGANFLIGAALNGAAVNLAMLIIGRILLGFGVGFANQAVPLYLSEMAPPKLRGALNICFQLATTIGILVANLVNYGTNKIHGRYGWRISLALAAVPAGIMTLGALGLPDTPNSLIERGHHEKAKATLQKIRGTDDVDAELQDMIEASEESARVQNPWRNIFQRHYRPQLVMAFVIPASQQLTGINVIMFYAPVLFKTLGFGANASLMSAVITGAVNVFATLVSIVTVDRVGRKGLFLEGGVQMILSQVVTIGAILGVGFGSSGEGQLSKNLANGVLILICLYVSAFAWSWGPLGWLVPSEIFPLEIRSAGQSIVVSVNFFWTFLVAQFFLMALCHLKYGLFFLFAAFVIVMTLFVVLFLPETKNVPIEEMNLVWRNHWFWKNYVLDEHLPSSGVGARAR

>AcSTP13 Aco011682.1

MEAFLKRFFPDVYGKMRGDTEISNYCKFDSQLLTVFTSSLYVAGLVSTLVASSVSSRFGRRTSMLVGGAVFVAGSAIGGAALNVYMLILGRVLLGVGLGFTNQSIPLYLSEMAPPQYRGAFSNAFELCISLGILIANLVNYGVQRIEGGWGWRISLSLAAVPASFLAFGALFLPETPSNVLQCSADHNQAKVLLQRLRGTSNVQKELDDLISASAVSRTIRHPFRNITKRKYRPQLVMAILIPFFNQITGINVINFYAPVMFRTIGLKESTSLMSTVVTRIFATTSNVIAIVAVDRLGRRVLFIVGGIQMIISQFTIGVILAKQLKDHGGMEKDYAYLVLTFMCIFVTGFGWSWGPLTYLVPTEISPLEIRSAGQSIVVAVMFMVTFVIGQTFLAMLCHLKYGTFLLFGGWVCVMTVFVYFLLPETKKIPMEQMEQVWRRHWFWKRVVGEEGVEVRTVEAANLSNPNTNPNPN

>AcSTP23 Aco018637.1

LVVPCPQTTSAERSSPSNPPFCRLSCLLSSRSLTMAGGVHKNHETTPTYEGRITTKYEGRITAFVVLSCITAAMGGVIFGYDIGVSGGVTSMEPFLKKFFPAVYKRMQEEAGEVSNYCKFDSQLLTAFTSSLYVAGLVATLLASRITGGAGRRTSMLLGGAAFVAGTAVGGAAIDVYMLILSRLLLGVGLGFANQAVPLYLSEMAPPQYRGAFNNSFQFSIGLGALSASLINYGTEKIKGGWGWRVSLALAAVPAALLTLGALFLPETPNSLIQQGKADRRNVANLLQRIRGCEDVGAELDDLIAASDAAAKIPPGRRQFEAIARREYRPQLVMAVLIPFFQQVTGINVIAFYAPILFRTVGLAEGASLLSAVVTGVVGAGSTFIAMMAVDRLGRRALLMIGGIQMFAAQVIVGGVIAAKLGDEGGMSEGYAYLVLVMIGVYVAGFGWSWGPLGWLVPSEIFPLEVRTAGQSITVAVSFLFTCVVAQTFLAMLCRLKAGIFFFFGGWLLVMTAFVYLLLPETKNLPIEQIGLVWREHWFWKRIVAGGDPVGGAK

>AcSTP25 Aco006734.1

MAGGFGGGGEGGRRADLYEGRITSYFVLACIVGSLGGSLFGYDLGVSSGVTSMDEFLKEFFPSVYRRKQAHLHETDYCKYDNQVLTLFTSSLYFAGLVSTFAASLVTRSYGRRISIMVGSVSFFFGGVINAAAQNIAMLIIGRILLGVGIGFGNQAVPLYLSEIAPYKIRGAVNQLFQLTTCLGILVADVVNYFTEKIHPWGWRLSLGLAVVPATLIFVGGFFLPETPNSLVEQGRLDEARKILEKVRGTPKVDAEFEDLKEASEAARAVKNPFRNLLKPRNRPQLIIGALGIPAFQQLTGQNSILFYAPVIFQSLGFGSGASLYASIITGSMLVVGALVSMSVVDRLGRRFLFIEAGIQMIGSMIIVTVTLALKFGHGETLSKGVAALLVVMICLFVVAFGWSWGPLGWLVPSELFPLEMRSAGQSVVVCVNLFFTAAIAQCFLAALCHLRYGVFILFAGLILIMSIFIILLLPETKQVPIEEISQLFEKHWYWKHIVSKDVELVQRHQKQQQQEAKTAAAV

>AcSTP26 Aco016897.1

MAGGGAAPIGIKKERAAQYKGRVTPFVVMACIVAAVGGSIFGYDIGISGGVTSMDPFLEKFFPVVYRRKNSGSQNNYCKYDNQGLAAFTSSLYLAGLVASIGASPVTRKYGRRVSIICGGISFLIGATLNAAAANLAMLLLGRIMLGVGIGFGNQAVPLYLSEMAPAHLRGGLNMMFQLATTLGIFTANMINYGTGKIKPWGWRLSLGLAAAPAFLMTVGGFLLPETPNSLIEQGRVEEGRRILERIRGTADVEAEFQDMIEASELANSIKHPFRNILEARNRPQLVMAIFMPTFQILTGINSILFYAPVLFQSMGFGGNASLYSSVMTGAVLALSTLISIATVDRWGRRVLLISGGIQMIICQVIVAVILGLKFGNDKHLSKEFSIIVVVVVCLFVAAFGWSWGPLGWTVPSEIFPLETRSAGQSITVAVNLFFTFVIAQAFLSLLCNLKFGIFLFFAGWITIMTVFVYVFLPETKGIPIEEMVLLWRRHWLWKRIMPPAPAVEDGWGAEMQVPDTSEYK

>AcSTP5d Aco014729.1

MAGGGFAAEFPASDYGGSLTTSVIVTCIMAASGGLIFGYDIGISGGVTTMESFLRSFFPSVLKKMAEAKQDDEYCMYDSQALTAFTSSLYVAGLAASLAAGRLTKAVGRQTIMLLGGALFFTGATVNASAANVEMLILGRILLGFGVGFTNQAAPVYLAEVSPARWRGAFTTGFQFFIGVGVVAANLTNYGAARLRSWGWRLSLGLAAAPACVFILGALLIPDTPSSLLHRGMPIDTARAALRRLRGSSADVDAELNDIARAAEDARRNEEGAFARMVLGRRYRPQLAMAVAIPLFQQLTGVTVVAFFAPVLFRTVGFGSDGALVGAVILGCVNLASILVSAFTVDRLGRRKLFIVGGAQMIICQVAVAWIMGAKIGADGEAAMPKGYAAAVLVLMCAFSAGFGLSWGPLSWIIPGEIFPVEVRSAGQAISVAVNLGCTFVQTQLFLAMLCRFKFATFAYFAAWVVVMTAFVAAFLPETKGVPLESMASVWEHHWYWARFIADDNRADNSKVNINIR

>AcSTP5e Aco014753.1

MDGGGFVVNGPASDYGGGLTFSMIVTCVVAASGGLIFGYDVGVSGGVTTMESFLRLFFPEVLRKMSEAKRDEYCLYDSQKLTAFTSSLYIAGLAASLVAGSLTKAVGRQAILLLGGALFFAGAAVNAAAVNVAMLIIGRILLGFGVGFTNQAWMRFAQKPAWYGSGQAAPVYLAEVSPPRWRGAFTTGFQFFLLIGVVVASVVNYATARLRRWGWRLSVGLAAAPAALLLLGALLIPDTPSSLLHRGMPDAARAALRRLRGPAADVDAELKDVARAVEDARRNEEGAFARILLQRRYRPHLVMAFAVPLFQQLTGVIVLAFFSPILFQTVGFGSNAALMGAVILAAVDLVAILVSTFTVDRYGRKVLFILGGVQMIISLTNSEQRTAGRGVVDNGVENRGERRRDHGEALRHGGGSTAVLLLGGVRVVVGPTGWIIPSEIFPVEIRSAGQGISVAVGLGCTFVQTQTFLAMLCRFKFATFAYYAGWVTLMTVFVAAFLPETRGVPLESMRSVWARHWYWGRFIKDDRNVVEDRT

>AcPMT1 Aco003836.1

MASPNPEAKVIAGSTPPALPPQPPTTMRSSLLEEVIVPEPKNRRNVRFAFACATLASMTSILSGYNIGVMSGAVLFIKEDLKISDTKVEILVGILNLYSLVGSFAAGRTCDWIGRRYTIVFASAIFFAGALLMALAPTYALLMLGRFVAGVGVGYALTIAPVYTAELSPASSRGFLTSFPEVFINSGILLGYVSSYAFAGLPLHLGWRVMLGVGAIPSVLLAMGVLAMPESPRWLVMKGRLAEAASVLRRTADTAEEAELLLLDIKKAAGIPADRTAEGVWQELLVRPTAPVRRILVSAVGMHFFQQASGIDSVVLYSPRVFEKAGIADKTELLGTTVAVGLVKTLFILVATFLLDRVGRRPLLLSSAAGMVASLAGLGIGLTVISHRTDGAAAWAVALSIASTLAYVAFFSIGLGPVTGVYTSEIFPMRLRALGCAVGVAVNRSTCGVVTMTFISLYNAIGIGGSFFLYGGIAAAAWVFFFTYLPETRGRTLEETGELFNEHPRQGEEEQLLQKQGPPMGNYRKV

>AcPMT2 Aco012301.1

MANPNPEAKVITGSTPPQPQPPPPPQPTNSLPEEAIVPGVKKRQNVRFAFACATLASMTSILLGYDIGVMSGAVLFIQKDLKISDTQVEVLTGILNLYSLVGSFAAGRTSDWIGRRYTIVFASAIFFAGALLMGFAPNYALLMLGRFVAGIGVGYALMIAPVYTAEVSPASSRGFLTSFPEVFINTGILIGYISNYAFSHLPLNLGWRFMLGVGAIPSVFLAVGVLAMPESPRWLVMKGRLAEAASVLHRTSDTAEEAELRLLDIKKAAGVPAGCTDDFFAPAKRQRGGESVWKELFLRPTPPVRRILVTALGIHFFQQASGIDSVVLYSPRVFQKAGITDKNKLLGTTVAVGFVKTLFILVATFLLDRVGRRPLLLSSTGGMVASLAGLGIGLTVIGHSAGGTVPWAVGLSIASILAYVAFFSIGLGPITWVYTSEIFPLRLRALGCAVGVALNRCTSGVITMTFISLYKAITIGGSFFLYAGIATAAWVFFFTYLPETRGRMLEDTGKLFGIMDTDKDVDVCDEQPPHKSVGEEKAIQLTNNNSSSNTTTETGTNRQLPKV

>AcPMT3 Aco012302.1

MEGNNTSQRKKMEQHLEEKRNKYAIACCIIASIISILMGYDTGVMSGAMLFIKKDLKIGDGQVEVLAGIINICALGGSLTAGRVSDWLGRRRTIAVGAAVFFVGSALMGAAPGFAALVAGRCVAGVGVGYALMIAPVYSAEVSAPATRGFLTSLPEMCISVGILLGYVANYLLAKLPLAYRWLVMQGQIKEAREVLLRVSNAPEEAELRLREIKSAAGISEASDDDGTVQLTKQPAGKGVWKELLLRPTPAVRRILIAAIGIHFFEHATGIEAVVLYSPRIFKKAGIRTQNKLLIATMGVGLTKTCFLLIATFLIDKVGRRPLLLTSLGGMALSLAGLGFGLTMVECSGERLGWDIALSIAAVFVFLAFFSIGLGPITWVYSSEIFPLRLRAQGASLGVAVNRLMNGIVSMTFISLYKAITIGGAFFLFAGIAVLAWLFFFLACPETRGRPLEEMEEVFSSSRRRRRRSTDGSAGSKQFEIAKMEGDQESASS

>AcPMT4 Aco010302.1

METVDPRTMGNGVSRIAGLGGKGKYRRMDAPIAEEEEEIDEGGGGAGAMAKGMRTSESRRYVFACAVFASLNSVLLGYDVGVMSGCILFIQRDLHITEVQQEVLVGILSFISLLGSLAGGRTADALGRKWTIGLAAIVFQIGAAVMTFAPSFLVLMIGRLLAGIGIGFGVMIAPVYIAEISPAVARGSFTSFPEIFINLGILLGYVSNYAFSGLSEHINWRVMLAVGIVPSVFIAFALFAIPESPRWLVMQKRVEEAREVLLKISDNEEEVKLRLAEIEEAAGLTNAEKYDGKSVWKEILRPSPVLGRMLITGLGIQCFQQITGIDASVYYSPTIFRDAGIKSDSQLLAATVAVGFTKTVFILVAIVLIDRVGRKPLLYISTVGMTICLFGLSLSLTLLEHELVSRGVGIGIAVLAVCGNVAFFSVGIGPICWVVTSEIYPLKVRAQAAGLGAVGNRVSSGLVAMSFLSISHAISVAGTFFIFSVLSALSVIFVYMFVPETKGKSLEEIEMLFGNEREWQGGELELGDVEHLVQKE

>AcPMT5 Aco000080.1

LHIAISIHSLPSHYRSPLLPKTMEEQNPTTQLSTTTTNTTSTPPKSTAPSMASLDIKPRRNKYALACAMLASMTTILIGYDVAVMSGAQLFIKEDLRVTDTQIEILAGVINLYSLLGSLAAGRTSDWIGRRYTMVLAAAIFFAGALTMGLAPDYAVLMVGRFVAGVGVGYALMIAPVYTAELSPAASRGALTSFPEVFINSGVLLGYVANFAFAKLPLRLGWRAMFLAGAVPPVFLGAGVLAMPESPRWLVMQGRLDDARRVLAKTTGGPDEAELRLQEIKESLGEHAKRRRGEGVWRELLVRPTPSVRRILLAALGLQFFQQASGIDSVVLYSPRVFQKAGIRSDSNSLGATVAVGFTKTAFILVATFFLDRVGRRPLLLASAGGMVASLLALASALRIIDDRAHDHSDGHGGAAAAVAIAAVLSFVGSFSIGLGPIAWVYSSEIFPLRLRAQGASMGTGMNRVMSGVITMTFISLYNAITISGSFFLYAGVAAMGWVFFYVFLPETRGRSLEDMEVLFGRKRVEEEKEKGKEGGEEGVVENKGDAEGESQENNC

>AcPMT6 Aco016335.1

MGLVDLKSSEKGIDGKMGFGGKKGTKYRRMDSEEEGEEGIHHLGERRREKGNSRKYVYVCALFASLNSILLGYDVGVMSGAIIYIQKDLHITEFEEEVLVGCLSIVSLLGSLSGGRTSDAIGRKWTMGLGAVVFQAGAAIMTFAPSFQILLIGRLLAGIGIGFGAMISAVYIAEISPAISRGTLTSLPEICINLGILLGYVSNYAFSGLSEHINWRIMLGVGILPSVFIGFALFVIPESPRWLVMQKRVKEARSVLLAISENEAEVEERLVEIEEAAGVLHAAKGSEEKAVWRELLSPSPAVARMLAAGCGIQIFQQITGIDATVYYSPTIFSNAGIKSDKEILAATVAVGFTKTMFILVAIFLIDRVGRKPLLYISTVGMTICLFMLGTALTLQKHGLGLVSPKIGIDLAILAVCGNVAFFSVGMGPICWVLSSEIFPLRLRAQASALGQVGGRVSSGLVSMSFLSVARVISVAGVFFIFSAVSVVSVVFVYVCVPETKGKTLEQIELMFQGGREWQKGELQLGDVEHLIQE

>AcPMT7 Aco016336.1

MSGAILFIQKDLRITELQEELLIGCLSIVSLLGSLLGGRTSDTVGRKWTIGLASIVFQCGAAVMAFAPSFLVLMIGRLLAGVGIGFGVMIAPVYIAEISPSVSRGSLTSFPEIFINLGILLGYISNYAFSGLSEHINWRIMLGVGILPSVFIGFALFVIPESPRWLVTKRRVKEARSVLRRISESELEAEERLVEIEQAAAAASASAKKHEEKAIWRELLSPSPSLLRMLVAGCGIQCFQQITGIDATVYYSPTIFRDAGIESENEILAATVAVGFTKTAFILVAIFLIDKAGRKPLLYVGTVGMTVCLFVLGIALTHQHGSGLLSPDVGIRLAIWAVCGNVAFFSIGIGPICWVLTSEIFPSRLRAQASALGAVGNRLSSGLVAMSFLSVSRQISVAGTFFIFSAVSALSIAFVHKFVPETKGKSLEQIEMLFQSAAEMRGGEVQLGDVEHLVQEE

>AcERD-6L1 Aco005379.1

MSFREESGSEDGGRSELKKPFLHTGSWYRMGMGSRQSSLMEKMGSSASVIRDSSISAFLCTLIVALGPIQFGFTAGFSSPTQESIISDLSLSLSEFSLFGSLSNVGAMVGAIASGQIAEYIGRKGSLMIASIPNIIGWLAISFAKDYSFLYMGRLLEGFGVGVISYTVPVYIAEIAPQNMRGALGSVNQLSVTIGIMFAYLLGMFVPWRLLAVIGILPCTVLIPGLFFIPESPRWLAKMGMMEDFESSLQVLRGFDTDITAEANEIKRAVASSRRRTTIRFADLKQKRYSVPLMIGIGLLVLQQLSGINGILFYAGSIFKAADEVSYQLVVKDLSAVAWGHAENLPARYGKCVNRDLRGLTNSDLATCGLGAIQVVATGITTWLLDKAGRRILLMVSTAGMTLSLLVVAVVFFLKGTQSEDSDLYFILSILSLVALVAYVISFSLGLGAIPWIIMSEILPVNIKSLAGSVATLANWLTSWLITMTATLMLNWSTGGTFTAYMIVSVFTLVFVILWVPETKGRTLEEIQWSFR

>AcERD-6L2 Aco006192.1

MITSGGGGGGDESGGEEAERSSSDLRRPFLHTGSWYKMSGAAVAAAGSGMGSAQSSLMGSSAMVLRDSSVSVVLCTLIVALGPIQFGFTGGYSSPTQDGIIADLHLSLSEFSIFGSLSNVGAMVGAIASGQMAEYIGRKGSLMIASIPNIIGWLAISFAQVPVYIAEIAPQNMRGALGAVNQLSITIGVLLAYLLGMFVPWRLLAVIGEVPNSFGTLPCIILVPGLFFIPESPRWLAKMGLMEDFETSLQVLRGFDTDISLEINDIKRAVASANKRTTLHLSHLNQKRYRTPLLLGIGLLVLQQLSGINGIVFYAGNIFKSAGLTDSNLATCGLGAIQVLATAVTTWFLDKAGRRILLIISTAGMTISLFFVSAAFFLEDIFSEDSHIHHVLSILSLVGLVANIVAFSFGLGAIPWIIMSEILPVNIKSLAGSVATLANWLTSFIVTMTANLLLNWSNGGTFTIYAIVSAFTLVFVILWVPETKGKTLEDIQWSFR

>AcERD-6L3 Aco014914.1

MGSMGDVERGIKVEARKEREVIREPLIQQNSKEVGSSDGETNSNSSCKSAHQDSIWMVLLSTAVAVCGSFEFGSCVGYSAPTQSGIIRDIGLSLSEAMRIAALICIVGWLAIYFAKLLICTGASVSFIVGTLVTWRTLVSIGVVPCIVLLVGLFFIPESPRWLAKVGQQKEFQVALRKLRGKNADITEEATEIQEYIESLQNLPQANLLQLFQSQHIRAVIVGVGLMVFQQLGGVNGVGFYASQIFVSAGFSSGNLGTILMGCIQVPVTMLGAILMDRSGRRPLLMISATGTFAGTFLTGLSFYLKGQGVCAEWVPTLALSGILVYMGAFSIGMGAVPWVIMSEIFSINIKAIGGSLVTLVNWFGSFAISYAFNFLMTWSSAGTFFLFSAVSAVTVLFVARVVPETKGKTLEEIQASLSSHR

>AcERD-6L4 Aco018778.1

METCNMHEVHGILSMLFLSNILQFSVFGSIVTIGAMIGAIASGRISDYIGRKGAMRLSAVVCIVGWLAIYFAQGAISLDVGRFSTGYGIGVFSYVVPVFIAEIAPKTLRGGLTTINQLLICTGLSAAYIIGTVVTWRTLALAGVIPCLVLLVGLAFIPESPRWLAKVGRQKEFEVALRCLRGKDADITQEAAEIQEFIETLQSLPRARIQDLFERTYIRPVIVGVGLMAFQQFGGINGIIFYASETFVSAGFASGSLGTILMGCVQVPITAFGALLMDRSGRRPLLMVSSSGLFLGSSITAVSFYLKSHGIYMDSVPIVALCGILVYIGSFSIGMGAIPWVMMSEIFPINIKGIGGSLVTLVNWFGSWAVSFAFNFLMSWSSTGTFVLFSMICATGVLFVAKVVPETKGRTLEEIQASLNAH

>AcERD-4L Aco014912.1

MEVGGEVGEETARPLMEAKGGRRPPSQPSILVVVASTAVAVAGSFVFGMSVGYSSPSQSGIMYDLDLSLAEAMAISDLFCIAGWLSIVFSKNFWWLDVGRFSVGCGIGVFSYVVPVYISEITPKNLRGGFATVNQLMICCGASLMYVVGTVLTWRVLAIIGLAPCLLQLLGLILIPESPRWLAKVGQHEEFEAALRRLRGKDADICEEAEEIKEFAENLRRLPQGSLFELFQKKYLHSVTVGVGLMVLQQFGGVNAIGFYASEIFVSAGFSSGNSGMLAMVAVQIPMTTLGVFLMDKAGRRPLLMVSAAGTCLGCFLVGMSFLSKDQEWVKELNMLLALAGILVYTGSFSLGMGGIPWVIMSEIFPINVKGVAGSLVTLVSWLGSWVVSYAFNFLMMWSSAGTFFMFACICGLTVLFVERLVPETKGRALEEIQASLNFF

>AcZIFL-2a Aco019261.1

MGDSTSRVPLLNKVYYAKCPGCAMDKRKESHTGIPYKEFLYVWIVTLCTALPISSLFPFLYFMIRDLNIAEREEDIGFYAGFVGAAFMLGRAITSVFWGIVADRHGRKPIIVIGIFSVIVFNTLFGLSVNYWMAIITRFLLGALNGLLGPVKAYASELCRPEHQALGMSLVSTAWGIGLIVGPAIGGYFAQPAEKYPEIFSPNSVWGRFPYFLPCLCISFFAVCVFISCLWLPETLHMHHNGKGAENETIEALEAQPFVSDSKQDATESEAPKENLFKNWPLMSAIIAYCIFSLHDMAYTEIFSLWAVSGKSYGGLSFSSQDVGQVLAISGFSLLVYQLFVYPRIEKILGPIYSCRLSAVISIPLLAAYPFLTNLSGIKLTIITNCASLMKNILSVTIITGLFILQNNAVPQNQRGAANGISMTAMSLFKAVAPAGGGAIFSWAQKRQHASFLPGDQMVFFILNLFEFIGFVLTFKPFLIVPGK

>AcZIFL2b Aco015001.1

MATTMTTTTTTMMTTTMTTPLLEEKMQRRYYNNCPGCRQDRKNAAHPGIPYRDFFYIWLVTLCSTLPIQSVFPFLYFMIRDLHIAKEEEDIGFYAGFVGSTYMFGRALTSVLWGVVADKYGRKPVIVISILSVIIVNSLFGLSSSYWMAITTRALLGLLCGILGPIKAYASEVSRKEHQALGLSLVGTSRCIGLIVGPAIGGFLAQPAEKYPNIFSQDSLFGRFPYFLPCLCISLFAVCALIACFWLPETLHMHHGDKLQDTSVETLEESLIDSDLKANGEIADEGGSVSKQSLLKNWPLMSAIIVYCVFSLQEMAYIEIFSLWAVSDRKYGGLNFSTEDVGEVLAITGVSLLVYQILLYPFVVKWLSPVRSIRLAAVLTIPLFVAFPFMSNLSGIVLKLVVNCASFVKNVFSVTVITGLNLVQNNAVCQEQRGAANGISVTAMSLFKAAAPAGGGALFSWAQKRTDASFLPGDHIVFFALNVIVVLGLLMTYKPFLAKPGAK

>AcZIFL2c Aco014996.1

MGNGANEPLLAAKKKVYYEKCPGCRQEQKNDSHAGVPYLEFFYIWIVCLCSTLPIQSLFPYLYFMIRDLNVAEREEDIGYYAGFVGAAYMFGRTLTAVLWGVVADKYGRKPVIVISILSVIIFNTLFGLSTTYWMAIATRALLGFLCGLLGPIKAYASEVCRKEHQALGLSLVTSSRAIALVVGPAIGGFLAQPATKYPNIFSENSIFGRFPYFLPSFAISVIAIAAFVACFWLPETLHMHHDVEDGSVESLEASLSGSDSDVNAKATEESEVTSKRSLFTNWPLMASITVYCIFSLHDTAYAEIFSLWAVSAKKYGGLSFSSQDVGMVLSISGFGVLVFQLLFYPPVVKRLGYITPFRAAAVLSIALLATYPFMTNLSGFMLQLAVNSASLLKNVFAATIMTACNILQNIAVPQEQRGAANGISVTAMSIFKAIAPAGGGAIFSWAQKHQDLPLLPGDQMIFFILILVEAVGLILSFKPFLRVPSSMTRS

>AcITR1 Aco008059.1

MTIDFSMPGSSGILDSPAKRDMSFFSNGYVLGLTVTAGIGGLLFGYDTGVISGALLYIRDDFKAVNDNYVLQETIVSMALVGAMIGAAGGGWVNDAYGRKKATLLADVVFTVGSLIMCAAPDPYVLILGRLLVGLGVGIASVTAPVYIAEAAPSEIRGGLVATNVLMITGGQFLSYLVNLAFTEVSGTWRWMLGVAAIPAIIQFILMLFLPESPRWLYRKNEKARAIEVLSKIYDPDRLEEEIDLLAVSSLDDRSKKSVSYLDVFRSKEIRLAFFAGAGLQAFQQFTGINTVMYYSPTIVQMAGFSSNQLALLLSLIVAAMNAVGTVVGILLIDRCGRRRLALTSLSGVTLSLLILSAAFFLQSSDLTSALCGSAALHTSTACGNRLGWFAVAGLALYIAAFSPGMGPVPWAVNSEIYPEAYRGVCGGMSATVNWVSNLIVAQIFLSVVAVLGTAATFLIIAGVAVLAFVFVLLFVPETKGLTFEQVERMWKERAWGSPLGSRESLLDGAA

>AcITR2 Aco005101.1

MEGGVHEADASAFRECFSLSWRNPYVLRLAFSAGIGGLLFGYDTGVISGALLYIRDDFSSVDKKTWLQESIVSTAVAGAIIGAAIGGWANDRFGRRASILVADFLFFVGAVIMASAPNPALLIVGRVFVGLGVGMASMTSPLYISEASPARVRGALVSTNGFLITGGQFLSYLINLAFTNAPGTWRWMLGIAGVPALVQFVLMLLLPESPRWLYRKRREEEAEAILRKIYPPDEVEGEIQALRESVEAEIEENKSSEKISFVKLFKTKTVRRGLVAGVGLQVFQQFVGINTVMYYSPTIVQLAGFASNQTALALSLVTSGLNAMGSIVSIYFIDRTGRKKLLIISLCGVVMSLAVLSAVFHETTSHSPSVSRSETAHFAGNTCPGYSAAATTPWDCMRCLKASSPDCGFCASGTNKLFPGACLISNSTVKDLCHGEGRLWYTRGCPSRYGWLALIGLALYIIFFSPGMGTVPWIVNSEIYPLRYRGVCGGIAATANWISNLIVAQSFLSLTQAIGTSWTFMIFGVLSLVALFFVLIFVPETKGLPIEEVEKMLEQWELSFRFCAKRPEKIKNNSGV

>AcITR3 Aco025488.1

MEGGVHKADQTEFRDCLHLTWKQPYILRLAFSAGIGGLLFGYDTGVISGALLYIQDDFKQVEKSTVLQETIVSMAVAGAIIGAGLGGWMNDRFGRKPSILIADLLFFIGAIVMAVAPIPGIIILGRIFVGLGVGMASMTSPLYISEASPARIRGALVSTNGLLITGGQFLSYLINLAFTKAPGTWRWMLGVAGIPAFVQFILMLMLPESPRWLYRKDRKEDAVNILRKIYPPSEVDTEIEALRQSVEAEIAEEGSVGGDGLFNKLKQAFSSTVVRRGLMAGVLCQVAQQFVGINTVMYYSPTIVQLAGFASNSTALALSLITSGLNAVGSIVSIYFVDRAGRRRLMLLSLVGIVTCLALLGGVFFGAASHSPAVSNVETRLFGNYTCSDYTPASGIRWKCTDCLKAASDCGFCAHGGNKLLPGACLASADAVKDACHAGHREWYTKGCPSNFGWLALIALGAYIISYSPGMGTVPWIVNSEIYPLRFRGLCGGLAAVANWVSNLIVAQTFLSLTEALGAAPTFLLFCGLSALAFVLIFFFVPETKGLQFEEVEKMLEKKGYRAWKKNSVKP

>AcpGlcT1 Aco026982.1

MTDRDRPGLGLSASSYSSPSKIKNQFNKNLFQADPTKTLQLQLINVTNIFYQQGDERSRRKKKQAVRISRWGGQGRQMVQSAAIAAKGGASASASGWGDLPGRSSLRRRWRPKIVPMPEGGGGGFCCGRSSPIGSASIGLGVEMARLRSGVESIFLRSRERPRYARVRASGDLEDVPSDKLPTKSSGTVLPYVGVACLGAILFGYHLGVVNGALEYLSKDLAITENTVLQGWVVSTTLAGATVGSFTGGALADKFGRTRTFQLDAIPLAIGAFLSATAQDVRTMIIGRLLAGIGIGISSAIVPLYISEISPTEIRGALGSVNQLFICIGILAALVAGLPLAGNPLWWRTMFGIAVVPSVLMALGMSFCPESPRWLFQQGRLPEAETAIKRLYGKERVAEVIHDLKAGSQGSTESDANWFDLFSKRYWKVVSVGAALFLFQQLAGINAVVYYSTSVFRSAGIASDVAASALVGAANVFGTAVASSLMDKQGRKSLLMTSFTGMAASMLLLSLSFTWKALAPYSGPLAVAGTVLYVLSFSLGAGPVPALLLPEIFASRIRAKAVALSLGMHWPLPVCRARYLHHPAPALDAPPSPRLVAAARCAAASLAPASLAVLPSLVPSDLALLPPPILTFEISRSHRRNGAGQGFGFVFLIESSRASELQYPSDIPAHRRPRSIGRSTSGHGSGEEKDGRSAKRRDGVAVAVLEVGGARRRGGESCVRLEIRFARCEGGQRQAGGIALSERR

>AcpGlcT2 Aco001011.1

MRSSSVLLLPHLNPITTSIRPRNLALHKLSPSLKGFRVAAVENKPSPSPLGSKSEPPKTLGADGEVDYVEGGVGEGGGGGGGVDLGWLPAFPHALTASMANFLFGYHIGVMNGPIENIARELGFEGNSFLEGLVVSIFIVGAFIGSISSASLIDKFGCKRTLQIDTIPLILGALISAQAHSLDEMLWGRFLAGLGIGVNTVLVPLYISEVAPTKYRGSLGTLCQIGTCLGIIASLALGIPSESDPHWWRTMLYTACVPGLIIMLGMQFAVESPRWLCKVGRLDDARKVVGRLWGMSEVEKSIEEIKSVVENDGAESSWLDLLEEPHKRVAFIGGSLFLLQQFAGINGVLYFSSLTFQDVGISSSALASLFVGITNLGGALVASYLMDKQGRKKLLIGSYLGMVCSML

>AcpGlcT-L1 Aco004425.1

MMHSKFNSSAYKRMSPKELTLVMDRDDESPVKTMNCAGQDDGNPSWRRSLPHVCVATITSFLFGYHVGVVNEPLESISLDLGFAGNTMEEGLVVSMCLGGAFFGCLFSGWIADGIGRRRAFQLSALPMIFGACLSASATSLEGMLAGRFLVGMGMGLGPPVASLYVTEVSPSSVRGTYGSFIQIATCLGLIGALLIGIPVKEYTAWWRVCFWVATVPAVLLAILMEFCAESPLWLYKRGRITDAEVQFERLLGGPHVKSAIAELSRSEKADEGESLSGINAVFYFSSTVFKSAGVPSNLANIFVGLANLSGSIIAMLLMDKLGRKVLLFGSFLGMATAMGLQSLAGGAHFLGSGALYLSVGGMLLFVLAFSLGAGPVPGLLLPEIFSNKIRAKAMALCMSVHWVVNFFVGLLFLQLLEQLGAQVLYSIFAAFCLIAAVFVKKNVLETKGKTLQEIEVALLSSE

>AcpGlcT-L2 Aco012247.1

MMGDGTEIYTVDDALVSMGFGKFQGLVLAYSGMGWVSEAMEVMLLSFVGPSVQAEWNLSSHEESLITSVVFVGMLIGAYSWGVVSDTYGRRQLFTYIYFFRTGFLFTALVTSLAGFLSAFSPNYLCLIVLRFLVGVGLGGGHVLASWFLEFVPAPSRGAWMVVFSFFWTVGTILEASLAWVIMPILSWRCLLGISSLPSFLLLLFFSVTPESPRYLCTNGRLHEAMFVLEKMASMNNKVLPSGVLTPSCTTSQVNYSLHTSEAMHLIALGKHEISNDEETSSNIGGISSLKRLLSPKLRRPTLLLWIVFFGNAFAYYGIVLLTSELSDVNRRCALIEVHSSQQQDANLYKDVFITSFAEIPGLVLSAAIVDRVGRKVSMWAMLFTSCAFLAPLIFQQKEAMTTSLLFGARTCIMGSFTVLYIYAPEVYPTSVRSTGVGVASSMGRIGGVVCPIVAVGLVENCRQMEAILLFEVVMLLAGLGAFLFPFETKCCELSDSLNSCN

>AcTMT1 Aco015779.1

MSGAVLVAIAASIGNLLQGWDNATIAGAVLYIKREFKLQNEPTIEGLIVAMSLIGATIITTFSGAVSDWVGRRPMLILSSILYFLGGLIMLWAPNVYMLLLARLIDGFGIGLAVTLVPVYISETAPPEIRGLLNTLPQFSGSGGMFLSYCMVFGMSLMPKPDWRVMLGVLSIPSLLYFALTIFYLPESPRWLVSKGRMVEAKQVLQRLRGREDVAGEMALLVEGLGVGGDTSIEEYIIGPANEPTDEHAAPSDKEQITLYGPEEGLSWVARPVKGSSALGSALALASRHGSMVNQNVPLMDPVVTLFGSVHEKLPETGSTLFPNFGSMFSVAENQPKTEHWDEESIQREGEENYASDNGEGDYDDNNLQSPLLSRQTTCMEGGKDVVVPKSANGSVLSMRRNSSLLQGSSSAVNSMGIGGGWQLAYKWSEKETLDGKKEGGFKRIYLHQEGVQGSRRGSLLSIPGVDAPEEGSEFIHAAALVSQSALYSKELLDQGQRPVGPAMVHPSEAAARGPVWRDLLEPGVKHALLVGVGIQILQQFAGINGVLYYTPQILEQAGVDVLLANIGIRPDSASILISALTTLLMLPAIGVAMRLMDLSGRRFLLLTTIPVLVASLIILVISNVVAMGTVLHAVLSTVSVIVYFCCFVMGFGPIPNILCAEIFPTRVRGICIAICALTFWFGDIIVTYTLPVMLNSIGLAGVFGIYSIVCVAAFIFVFLKVPETKGMPLEVITEFFAVGAKQVQAAKE

>AcTMT2 Aco011916.1

MAGAALVAIAASIGNLLQGWDNATIAGAVLYIKKEFKLESEPTIEGLIVAMSLIGATIITTFSGAVSDCVGRRPMLIVSSVLYFVSGIVMLWSPNVYVLLLARLIDGFGIGLAVTLIPVYISETAPADIRGLLNTLPQFSGSGGMFLSYCMVFGMSLMPKPDWRIMLGVLSIPSLLYFALTVFYLPESPRWLVSKGRMLEAKRVLQRIRGREDVSGEMALLVEGLGVGGDTSIEEYIIGPANELIDDQGETVDKDQVTLYGPEEGLSWVARPTKGQSALGSALTIVSRHGSVQNQNIPLMDPLVTLFGSVHEKLPEMGSMRSTLFPNFGSMFSVAEQQPKTEQWDEESLHREGEDYASEDAGGDSDDNNLQSPLLSRQTTSLEGKEITQAQTGHDSILSMGRNSSLMRRSTTGEAVSSMGIGGGWQLAWKWSEKEGVDGKEGGFRRIYLHQEGGAGSRRGSLLSIPGGETPEESEFVQAAALVSQPALFSKELMDQHPVGPAMLHPSETAVKGPRWHDLFEPGVRHALIVGVGIQILQQFAGINGVLYYTPQILKQAGVEVLLANIGISSDSASILISAITTLLMLPSIGIAMRLMDICGRRFLLLATIPILIASLFVLVVANVAQLSTVLHAVLSTISVIIYFCCFVMGFGPIPNILCAEIFPTSVRGVCIAICALTFWAGDIIVTYTLPVMLTAIGLAGVFGIYAVVCVFAFIFVFLKVPETKGMPLEVITEFFAVGAKRAQAAKSSSAVLG

>AcTMT3 Aco013008.1

MLGAVLVAIAASIGNMLQGWDNATIAVAVIYIKKEFKLESAPTIEGLIVAMSLIGATIITTFSGPVSDWVGRRYMLISSSILYFLSGLLMLWSPNVYVLLLGRLIDGFGIGLAVTLVPVYISETAPPEIRGQLNTFPQFSGSGGMFFSYIMVFFMTLAPIPNWRVILGVLFIPSVLYIVLTIFFLPESPRWLVSKGRMNEAKKVLQMLRGREDVSGELALLVEGLGISKDTTIEEYIIGPATDLHADQDPAAEKDKIKLYEPEEGLYWVAQPVGQSTLGSAVGLASRHGSIIDQIKDPIVTLFESVHEKLPDMGSTGSMTFPNFGSMFSIAEPLPKTEHSDEEHEHEEYSSESEGRNSDVDLEAPLISHRNGSTLSVRGHSSLLQANDGEVVNSTGIGGGWQLAWKWSERENEKGEIEGEFKRIYLHQEGPAPSQRGSVVSLTGTVVPENADYIHAAALVSQSALFTRELMNQHLAGPAMVHPSKTDSTGPRWRDLCEPAVKRALLAGVGIQILQQFSGINGVLYYTPQILEQAGVGVLLANMGLSSDSASILISGLTTLLMLPCICVAMRLMDVSGRKSLLVYTIPVLIASLFFLVLSTTINFGTLVNASISTVSVTLYICCFVMGFGPIPNIFCSEIFPTRVRGLCIAICSLTFWFGDIVVTYTLPVMLKSVGLAAVFGFFGVVCCVALAFVIIFVPETKGFPLEVIIEFFNVGAKMH

>AcvGT1 Aco026737.1

MESSSFQISQAAPLLPSSSSSSSAPRLRVRSQAQGEYSSHSPPPDTAGDAADRQESFNWPSVLLPFLFPALGGLLFGYDIGATSGASISLQSADLSGTNWFNLSAVQLGLVVSGSLYGALAGSIIAYPIADFLGRRMELIAASALYITGGLITGYAPNLAVLIIGRLIYGIGIGLAMHGAPLYIAETSPSQIRGTLISLKELFIVLGILLGYLVGSAQINVIGGWRYMYSFSAPIAVIMGLGMGRPAGDKISEREIDDTLVSLKSAYAEQEQEGSFWEVFEGASLKAFIIGGGLVLFQQVLTGQPSVLYYAASILQSAGFSAASDAARVAVVIGLFKLVMTGIAVLKVDSLGRRPLLIGGVGGIVVSLFLLAAYYKILAGFPLVAVGALLLYVGSYQVSFGPISWLMVSEIFPLRTRGRGISLAVLRQFRIKCFSNLCFLTIEVVLEKMQNLNLQDRYSTELLGPDNIFLLFGGIALLSLLFVLFKVPETKGLSLEEIESKILK

>AcvGT2 Aco017005.1

MADPEQPKAASFFARPLGRDGVVNEIDTVREPLIGGSGPSESYSVCAAILPFLFPALGGLLYGYDIGATSGATISLKSSTLSGTTWYNLSSVEIGLVVSGSLYGALIGSALAFTVADSLGRRRELILASLLYFIGALLTALAPDFAVMVIGRFVFGTGIGLAMHAAPMYIAETAPSQIRGTLISLKEFFIVIGMLLGYISGSIYVELVAGWRYMYATSAPLCLIMGLGMWWLPPSPRWLLLCAIQGKGSILDTKEFAVRCLCRLRGQALDDSISVQVDAILAELSYAGQEKAATLSEIFQGKCLKALIIGCGLVFFQQVTGQPSVLYYAATILQSAGFSAASDATRVSVLLGVLKLLLTGIAVLVVDRLGRRPLLIGGVSGIVISLFLLSSYYTLLNDFAFVAVIALLLYVGCYQLSFGPIGWLMISEVFPLRLRGRGLSIAVLVNFASNALVTFGFSPLQALVGTGVLFAGFGVIALASLLFIIFVVPETKGLTLEEIEAKIL

>AcSWEET1 Aco011302.1

MDVVHVLHFLFGIFGNVTALFLFLSPILTFRRIQKNKSTEDFSGVPYNMTLLNCLLSAWYGMPFVSPNNLLVSTINGAGTAIELVYVIIFLTYASTSKVRMKMLGLLMIVSSIFAAVVLISLLALHGQSRKLFCGSAATIFSICMYASPLSIMRLVIKTKSVEFMPFFLSLFVFLCGTSWFIYGLLGRDPFVTVPNGCGSFLGALQLILYMIYRDRKGRDTTKDDSIEMEESKSAKTTNTKPMEKINMQVHHQIDHQV

>AcSWEET2 Aco016508.1

MMRSLDFFFSFSYLLLLLHILFMNSVTMQQELQVTSLPLPTYKRIIRNKSTEQFSGLPYVYSLLNCLICMWYGLPCVSYGVILVATVNSIGAAFQLVYVSLFIIYADTTRKNLVIKTKSVEFMPFYLSLATFLMSVSFFAYGMLLHDFFIYIPNGIGTVLELLRVSQVFLISLAVDEFFRNWTGSEAIYKPSDN

>AcSWEET3 Aco010708.1

MGSLSLLFCSKYEEKNKRLFIDKLPGCCFAGNAASLLLYAAPILTFKRVIKKRSTEEFSCVPYIIAFLNCLLYTWYGLPVVSRGFENFPVITINAIGIVLETSFIFTYIWFALPERKASPNCFPCFRNFLIFSGHFAQKTAILMMVPVTVLFTMTVFVSSFLLHDHRHRKVFVGTIGLVASISMYSSPLIAVKEVIKTRSVEFMPFYLSFFSFLASSLWLAYGLLGRDLFLAAPNFLGSPVGLLQLILYFIYRKNKAAPEELHEIDLNKNDLKTVPKQQEVNGRKLDA

>AcSWEET4 Aco006346.1

MSNRWNSTSLAAVAEKDERKESTSRISKTVITTKSVEFMPFLLSLFLFLNGGVWTVYAILDRDIFLGPATFQIWKWLGNGLEIRMVQRDTSSEKVNRDSHHHPRMFWELTGRPGHHRFDQSYLFSAQPLRVLDRCQCCDEEYVLSKEKKGALEGPIAAPMHDSFFTFRNKETLPL

>AcSWEET5 Aco005793.1

MAWSRKYYIVGVVPITSKPKITPIRRRPCREYMCTVVKGRKQQPPIKPTFYRIWKKGAVEQFSAAPYLATLLNCMMWVVYGLPLVHPHSTLVLTINGLGLLIELCYVLLFLLFSDGRKRLRVFLILAAEVAFVVAVVLLVLTLAHTHDRRTLVVGIICVLFGTMMYAAPLTIMKLVIQTKSVEFLPLYLAVAFFLNSACWTTYALIRFDLFITIPNGLGLLFAVAQLVLHCVYYKSTKQQMEARRKMTELSLAEVVVPEESCRTSTTATTTPKWPPLRQPQDTKSPDA

>AcSWEET6 Aco004463.1

MVSADTIRTAVGVVGNAIALGLFLSPAPTFYTIWKKGSVEQFSPVPYLCTLLNCMMWVVYGIPMVHPHSMLVITINGSGLAIELAYVLLFLVYSRGKKRLQVFVVLVAETAFVALVALLVLTLAHTHEQRSMIVGVLCVIFGTMMYAAPLSVMKMVIQTKSVEYMPLSLSLASFFNGICWTAYALIRFDPYITIPNGLGVMFAVAQLVLYVMYYKSTQQQIEARRRKGEVGMEEVVVIGDGHRVGSSQSNGQKAQT

>AcSWEET7 Aco006158.1

MLAKKMVSANAVRYVVGIIGNVISFGLFLAPVPTFAKIWKRRAVEEFAPIPYLVALLNCMLWVFYGIPVVHPHSILVTINGVGFILESLYLLTFSAFSPNRLRLKVLRILSVELLFMGGVILGAHTHEKRSLIVGVLCIIFGTCMYASPLFIMKLVSHTKSVKFSLVDFLNGICWTIYAFFRSLSLSLYTTRRTRCLLLLFVAAVSHSLELKKLLRSTDSSIETSFANTRALWNPLIGHDGRFIACSGKNLFAFDRNGSVAWIVPLYYVCRKDISPVRDERGNVYLIAGERVLRITPSKIGTAEPPLEIFYSHNSTRLVSEEIIGLAISASYASLFITIRNRGLFAFLLRGELLWSAGPMLHRFGYRQGCKKNTSDCYFNSGPVVDRCEGTLYISNTKGQLYSLYIRSPQFRWIQDLSSFDTLMTITAGNNGRLYVIFPRKAMLMALDVSTGSILWQHPVGPLSGEKSFPIVDSNGWISIGSLDGNLYSISPNGDTKKFLEATSTDSVIHASPVVDCSGFSVYVSQTVMEAKSSRTVDNYTYISAMKPLSVLFTLLAPATGTIYWTGQYPGEMSSLLSRSDLRYFVLDERIFLTVLSAARKGNNLPCYTTRQRIAWTCSQAKPKIATTDIGDDKAILLFLFFQLVIVIILAVIVRFCCIFWRKKKLQGAGLQRFLEKRRSLHIRRKTLNKIISELEQKAAEDATAPNEALEQLGEIVKAKEGVERKLSTSYSLGRDRIGAKQRPTILPLYNGKAKSHSFHSTRTESVTIFNTLSNTSTSEEGESSSSSSSSSSYKSEGGNSDSSERDENLRRDAETESNVKGKVAEAVPSRRSTEIEEELAIDPMYVEKKIESSAHLEHEEQLKHGFGGDRRMWLKRRRTMSSTN

>AcSWEET8 Aco006156.1

MVSADAVRNVVGIIGNVISFGLFLSPVPTFAKICKRRAVEEFSPIPYLATLLNCMLWVFYGIPVVHPHSILVVTINGVGFVLESFYILIFLAFSPNRLRLKVVGILAAELLFMAGVITGVMVGAQTHEKRSLIVGVLCVIFGTCMYASPLSIMKLVIQTKSVKYMPFFLSLVSFLNGVCWTIYAFIRFDIFVTIPNGLGALLGLAQLILYLCYFRSTPRDENKPDAQPPAVTSAVAPSASNIPLATITVDK

>AcSWEET9 Aco006155.1

MMALSANIPNSGGESPHPCNLISVALFLSPLPTIAKIRRRRAVEEFSPKPYLVTLFNCMLWMFYSLPPVHPSTLVAVFDGVGVVLELFYICIFIYFADNRMRWMLGGIVAAELSSMAGIAAGVILGVREAETRSFIVGVGCVICGVAMNAAPFSTMKLVIQTRSVEYMPFFVCLAGFLNSVCWTIYGFLTSNIFLIIPNGLGILVGLAQLILYFWYRKSTPRRDNEPSISDPFLPPSQQTDRTSEPAATSTGEPSTSSPLSQPPQQRDRSSEPAATSADQPSTSRPLSPPPQQTDRSSEPAATTADQPSTSSPLSPPSQLIDISSEQAATSADQPSTRNALSTPSQQTDHDISSDRAVMSAIQPSTSIALLPPSPLQIYTHPIAYIQIECSLLFSLLFSLLLSSLLILLLSLTPTWFGLQSPN

>AcSWEET10 Aco016418.1

MTDPDTIRSIIGIIGNVISFGLFLSPVPTFIKIWKQKAVEQFSPIPYLATLLNCMLWVFYGLPIVHPNSILVVTINSTGLILEGIYLTIFFLYSPCKLRIKVLMYLAAEIAFVAAVVAVVLTTAHTHDLRSLIVGILCVIFGTCMYASPLAVMKLVIQTRSVKYMPFTLSLAGFLNGVCWTTYALIRFDIFITIPNGLGALFGLIQLVLYAIYFRATPQDQPAGEVELPSKTLEV

>AcSWEET11 Aco001900.1

MLVIRIENPLATGVGLIGNVLSFLVILAPLPTFYRVYKKKSTEGFHSTPYVVALFSAMLWFYYALLTTDVLLFTINALGSVIESIYLVMYIAYAHKKARAFTLKLILLLDVGFYGCVVLITVLFLRGRLRVKIMGWICASFATAVFIAPLSIIRQVIRTKSVEFMPFTLSLFLTLSAVAWFCYGLMLKDSYIALPNVVGVLFGLAQMLLYFIYMNGKKETPVSEITDQDKNPATLANPNADIEMNKESIGVTTPEVESSMSVAGKNEA

>AcSWEET12 Aco019048.1

MAVLSLHHPCTFTFGILGNIISLMVYLAPLPTFYRVYKKKSTEGFQSIPYIIALFSSMLWIYYAILKPDASLLITINSIGCAIEAIYIITYLVFAPKNVKIFTAKILLLLIVGAYGTILLLTLLLSKDSSRVHLVGWICMGFSVTVFVAPLSIMRLVIRTKSVEFMPFSLSFFLTLSAVVWFFYGLLTKDIYVALPNVLGFLFGIMQMVLYVAYKDVKPITQEYKLPEKGLGVHPVDSKPSKIENKDHHIVIEGDPKMLDGTEGDNRKETQEGNKMSHVEV

>AcSWEET13 Aco004628.1

MAALDHPWAFAFGLLGNLISFVVYLAPIPTFCRVYRAKSTQGFQSVPYVVALFSAMLWIYYALVNTHATLLITINSFGCMIESIYIIMYLVYAPRKAKIQTFGLIFLLNVVVFSLIILFTLLLATGSNRVKLLGWICVVFSVSVFVAPLSIIRLVIQTRSVEYMPFYLSFFLTISAIIWFLYGFLTKDLYVQLPNVLGFTFGFVQMALYWYYKRLKKAVVSPALPDDVVTIVTLSPIPVLEVHRIESDSSEVPNEIADGKTEEAKEANNEESPV

>AcSWEET14 Aco016039.1

MAPLPLNHPLVLIFGILGNFISCVVYLSPVPHFTAFIGRNRPKGSIGALRCRIIQCHAVDILPRSLNRTKAPPISINSFGCLIETVYIVMYLVYAPKECQNFYSKNATSFERRSLCLILLLTLLLSKARSSSHSRLGLCGLLRERFRRTTKLVIRTKSVEFMPFSLSFTLTLSAVMPNILGFLFGIMQMVLYIAYKNKRPMIEEYKLPERSCRDREPKRDDREGEVHPIDLKPQHACGEGPEAGGGRKEYEQVADYSSSGYGGGSGEKDIEALEKGEMGHVEV

>AcSWEET15 Aco003627.1

MGVLSLHHPWAFSFGILGNIISFMVYLAPVPTFYRIYERKSTEGFQSVPYVVALFSAMLWIYYAFLKANAYLLITINSIGCLIETVYIVMYLIYAPKSAKIFTAKILLLMNVGIFGSILLLTLLLCKGANRVIILGWVCVGFSISVFVAPLSIIRLVIRTKSVEFMPLSLSFFLTLAAVVWFAYGLLIKDKFVALPNVLGFLFGMIQMVLYITYKDVKPVAKPITQEHKLPEHIVAIVKVDATRGRGSSKPPHADKEEKGNKIRG

>AcSWEET16 Aco017831.1

MAPLPLNHPLVLIFGILGNFISCVVYLSPVPTFYRIYWKKSTEGFQSVPYVVALFSAMLWIFYALVKSNEGLLISINSFGCLIETVYIVMYLVYAPKSAKIFTVKILLVLNVGVFALILLLTLLLSKGSDRVHILGWVCVGFSVSVFAAPLSIIRLVIRTKSVEFMPFSLSFSLTLSAVVWFGYGLLTKDKYVAIPNILGFLFGIMQMVLYIAYKNKRPMIEEYKLPEQVVEIVKLSVTAERGSEVHPIDLKPQHACGEGPEAGGGRKEYEQAVDDSSSGDGGSGEKDIEALEKGEMGHVEV

>AcSWEET17 Aco002476.1

MADLSFIVGIVGNVISILVFASPIDTFRRIVKKKSIENFKGLPYVITLLSTSLWTFYGLLKPGGLLIVTVNGAGAALEAIYVTIFLIYAPNDMRMRMVKLVGLLNVGFLGVVVLVTLLALHGGVRIVVVGVLCAGLTIGMYAAPLAAMSMVVRTKSVEYMPFFLSFFLFLNGGVWSIYSVLVRDYFIGVPNAIGFVLGSTQLILYAIYRKKTPPPPKKASEPDEEEGSAHLMTQAEMAQLKPLSKGASLPKPARQHSLTNNIMKSLSTTAYELQSIWYQNEYIKSPEENHLSK

>AcSWEET18 Aco006347.1

MDSLLFFIGVVGNVISVLLFASPIKTFWRIVRRQSTEGFKATPYIVTLLSSSLWVYYGITKPDAYLVATVNGVGIILEAIYVALFLIFASPPLRAETAIWVLILDVGIFGLVVAVTKLAVNGSLRILVIGSIGAFLNVLMYGSPLTLMSF

>AtSUC1

MGAYETEKPTKDAAALETQSPEDFDQPSPLRKIISVASIAAGVQFGWALQLSLLTPYVQLLGIPHKWSSLIWLCGPVSGMIVQPIVGFHSDRCRSKFGRRRPFIATGAALVAVAVFLIGYAADFGYKMGDKLEEKVKVRAIGIFALGFWILDVANNTLQGPCRAFLADLAAGDAKRTRVANAFFSFFMAVGNVLGYAAGSYTNLHKMFPFTMTKACDIYCANLKTCFFLSITLLLIVTVTSLWYVNDKQWSPPPRNADDDEKTSSVPLFGEIFGAFKVMKRPMWMLLIVTALNWIAWFPFLLFDTDWMGREVFGGDSDGNERSKKLYSLGVQSGAMGLMFNSIVLGFMSLGVEWIGRKLGGAKRLWGIVNFILAAGLAMTVLVTKFAEDHRKTAGDLAGPSASVKAGALSLFAVLGIPLAITFSTPFALASIFSSCSGAGQGLSLGVLNLAIVIPQMIVSLGGGPFDALFGGGNLPAFIVAAIAAAISGVLALTVLPSPPPDAPKATTMGGFH

>AtSUC2

MVSHPMEKAANGASALETQTGELDQPERLRKIISVSSIAAGVQFGWALQLSLLTPYVQLLGIPHKWASLIWLCGPISGMLVQPIVGYHSDRCTSRFGRRRPFIVAGAGLVTVAVFLIGYAADIGHSMGDQLDKPPKTRAIAIFALGFWILDVANNTLQGPCRAFLADLSAGNAKKTRTANAFFSFFMAVGNVLGYAAGSYRNLYKVVPFTMTESCDLYCANLKTCFFLSITLLLIVTFVSLCYVKEKPWTPEPTADGKASNVPFFGEIFGAFKELKRPMWMLLIVTALNWIAWFPFLLFDTDWMGREVYGGNSDATATAASKKLYNDGVRAGALGLMLNAIVLGFMSLGVEWIGRKLGGAKRLWGIVNFILAICLAMTVVVTKQAENHRRDHGGAKTGPPGNVTAGALTLFAILGIPQAITFSIPFALASIFSTNSGAGQGLSLGVLNLAIVVPQMVISVGGGPFDELFGGGNIPAFVLGAIAAAVSGVLALTVLPSPPPDAPAFKATMGFH

>AtSUC3

MSDSVSISVPYRNLRKEIELETVTKHRQNESGSSSFSESASPSNHSDSADGESVSKNCSLVTLVLSCTVAAGVQFGWALQLSLLTPYIQTLGISHAFSSFIWLCGPITGLVVQPFVGIWSDKCTSKYGRRRPFILVGSFMISIAVIIIGFSADIGYLLGDSKEHCSTFKGTRTRAAVVFIIGFWLLDLANNTVQGPARALLADLSGPDQRNTANAVFCLWMAIGNILGFSAGASGKWQEWFPFLTSRACCAACGNLKAAFLLAVVFLTICTLVTIYFAKEIPFTSNKPTRIQDSAPLLDDLQSKGLEHSKLNNGTANGIKYERVERDTDEQFGNSENEHQDETYVDGPGSVLVNLLTSLRHLPPAMHSVLIVMALTWLSWFPFFLFDTDWMGREVYHGDPTGDSLHMELYDQGVREGALGLLLNSVVLGISSFLIEPMCQRMGARVVWALSNFTVFACMAGTAVISLMSLSDDKNGIEYIMRGNETTRTAAVIVFALLGFPLAITYSVPFSVTAEVTADSGGGQGLAIGVLNLAIVIPQMIVSLGAGPWDQLFGGGNLPAFVLASVAAFAAGVIALQRLPTLSSSFKSTGFHIG

>AtSUC4

MATSDQDRRHRVTRNRPPIARPSTSSSRPVVSPPRSKVSKRVLLRVASVACGIQFGWALQLSLLTPYVQELGIPHAWASVIWLCGPLSGLFVQPLVGHSSDRCTSKYGRRRPFIVAGAVAISISVMVIGHAADIGWAFGDREGKIKPRAIVAFVLGFWILDVANNMTQGPCRALLADLTENDNRRTRVANGYFSLFMAVGNVLGYATGSYNGWYKIFTFTKTVACNVECANLKSAFYIDVVFIAITTILSVSAAHEVPLASLASEAHGQTSGTDEAFLSEIFGTFRYFPGNVWIILLVTALTWIGWFPFILFDTDWMGREIYGGEPNIGTSYSAGVSMGALGLMLNSVFLGITSVLMEKLCRKWGAGFVWGISNILMAICFLGMIITSFVASHLGYIGHEQPPASIVFAAVLIFTILGIPLAITYSVPYALISIRIESLGLGQGLSLGVLNLAIVIPQVIVSVGSGPWDQLFGGGNSPALAVGAATGFIGGIVAILALPRTRIQKPIPLP

>AtSUC5

MGALEAERAANNATALETQSSPEDLGQPSPLRKIISVASIAAGVQFGWALQLSLLTPYIQLLGIPHKWSSYMWLCGPISGMIVQPIVGYHSDRCESRFGRRRPFIAAGVALVAVSVFLIGFAADMGHSFGDKLENKVRTRAIIIFLTGFWFLDVANNTLQGPCRAFLADLAAGDAKKTRVANACFSFFMAVGNVLGYAAGSYTNLHKMFPFTMTKACDIYCANLKTCFFLSITLLLIVTFSSLWYVKDKQWSPPQGDKEEKTSSLFFFGEIFGAVRHMKRPMVMLLIVTVINWIAWFPFILYDTDWMGREVYGGNSDGDERSKKLYDQGVQAGALGLMFNSILLGFVSLGVESIGRKMGGAKRLWGCVNFILAIGLAMTVLVTKSAEHHREIAGPLAGPSSGIKAGVFSLFTVLGIPLAITYSIPFALASIFSTNSGAGQGLSLGVLNIAICIPQMIVSFSSGPLDAQFGGGNLPSFVVGAIAAAVSGVLALTVLPSPPPDAPAMSGAMGFH

>AtSUC6

MSDLQANKDAAAVNRQSSSSSADLNGPSPMRKMISVASIAAGIQFGWALQLSLLTPYVQLLGVPHKWSSFIWLCGPVSGLLVQPSVGYFSDRCKSRFGRRRPFIAMGALLVAVAVVLIGYAADFGHSMGDKVDEPVKMRAVVIFALGFWILDVANNTLQGPCRAFLGDLAAGDAKKTRTANAFFSFFMAVGNVLGYAAGSYTNLYKIFPFTMTKACDIYCANLKSCFFLSITLLLVVTIIALWYVEDKQWSPKADSDNEKTPFFGEIFGAFKVMKRPMWMLLIVTALNWIAWFPFLLYDTDWMGREVYGGDSKGDDKMKKLYNQGIHVGGLGLMLNSIVLGFMSLGIEGISRKMGGAKRLWGAVNIILAVCLAMTVLVTKKAEEHRRIAGPMALPTDGIRAGALTLFALLGIPLAITFSIPFALASIISSSSGAGQGLSLGVLNMTIVIPQMVVSFGVGPIDALFGGGNLPGFVVGAIAAAISSVVAFSVLP

>AtSUC7

MSDLQANKDETTVDRQSSSSVDLDGPSPLRKMISVASIAAGIQFGWALQLSLLTPYVQLLGVPHKWPSFIWLCGPVSGLLVQPSVGYFSDRCTSRFGRRRPFIATGALLVAVSVVLIGYAADFGHSMGDKIDKPVKMRAVVIFALGFWILDVANNTLQGPCRAFLGDLAAGDAQKTRTANAFFSFFMAVGNVLGYAAGSYTNLYKIFPFTMTKACDIYCANLKSCFFLSITLLLVVTIIALWYVEDKQWSPKADSDNEKTPFFGEIFGAFKVMKRPMWMLLIVTALNWIAWFPFLLYDTDWMGREVYGGDSKGDDKMKKLYNQGIHVGALGLMLNSIVLGVMSLGIEGISRKMGGAKRLWGAVNIILAVCLAMTVLVTKKAEEHRRIAGPMALPTDGIRAGALTLFALLGIPLAITFSIPFALASIISSSSGAGQRLSLGVLNMAIVIPQMIVSFGVGPIDALFGDGNLPGFVVGAIAAAVSSIVAFTVLP

>AtSUC8

MSDLQAKNDVVAVDRQSSSSLADLDGPSPLRKMISVASIAAGIQFGWALQLSLLTPYVQLLGVPHKWSSFIWLCGPVSGLLVQPSVGYFSDRCTSRFGRRRPFIATGALLVAVAVVLIGYAADFGHSMGDKIDKPVKMRAVVIFALGFWILDVANNTLQGPCRAFLGDLAAGDAKKTRTANAFFSFFMAVGNVLGYAAGSYTNLYKIFPFTMTKACDIYCANLKSCFFLSITLLLVVTIIALWYVEDKQWSPKADSDNEKTPFFGEIFGAFKVMKRPMWMLLIVTALNWIAWFPFLLYDTDWMGREVYGGDSKGDDKMKKLYNQGIHVGALGLMLNSIVLGIVSLGIEGISKKIGGAKRLWGAVNIILAVCLAMTVLVTKKAEEHRRIAGPMALPTDGIRAGALTLFALLGIPLAITFSIPFALASIISSSSGAGQGLSLGVLNMAIVIPQMIVSFGVGPIDALFGGGNLPRFVVGAIAAAISSVVAFTVLP

>AtSUC9

MSDIQAKEDAAPVDRQSSSSVVVPDEPSPLRKMISVASIAAGIQFGWALQLSLLTPYVQLLGVPHKWSSFIWLCGPISGLLVQPTVGYFSDRCKSRFGRRRPFIATGALLVALAVILIGFAADFGHTMGDKLDEAVKIRAVGFFVVGFWILDVANNTLQGPCRAFLGDLAAGDAKKTRTANAIFSFFMAVGNVLGYAAGSYTNLHKIFPFTVTKACDIYCANLKSCFIISITLLIVLTIIALWYVEDKQWSPNADSDNEKTPFFGEIFGAFKVMKRPMWMLLAVTALNWIAWFPFLLYDTDWMGREVYGGDSAGDDKMKKLYNHGIQVGSLGLMLNSIVLGVMSLVIGVISKKIGAKRLWGAVNIILAVCLAMTVLVTKKAEEHRKIAGRMALPTNAIRDGALSLFAILGIPLAITFSIPFALASIISSSSGAGQGLSLGVLNMAIVIPQMIVSFGVGPIDALFGGGNLPGFVVGAIAALISSVVALTVLP

>OsSUT1

MARGSGAGGGGGGGGGGLELSVGVGGGGGARGGGGGEAAAAVETAAPISLGRLILSGMVAGGVQYGWALQLSLLTPYVQTLGLSHALTSFMWLCGPIAGMVVQPCVGLYSDRCTSKWGRRRPYILTGCVLICLAVVVIGFSADIGYAMGDTKEDCSVYHGSRWHAAIVYVLGFWLLDFSNNTVQGPARALMADLSGRHGPGTANSIFCSWMAMGNILGYSSGSTNNWHKWFPFLKTRACCEACANLKGAFLVAVIFLSLCLVITLIFAKEVPFKGNAALPTKSNEPAEPEGTGPLAVLKGFRNLPTGMPSVLIVTGLTWLSWFPFILYDTDWMGREIYHGDPKGTDPQIEAFNQGVRAGAFGLLLNSIVLGFSSFLIEPMCRKVGPRVVWVTSNFLVCIAMAATALISFWSLKDFHGTVQKAITADKSIKAVCLVLFAFLGVPLAVLYSVPFAVTAQLAATRGGGQGLCTGVLNISIVIPQVVIALGAGPWDELFGKGNIPAFGLASGFALIGGVAGIFLLPKISKRQFRSVSMGGGH

>OsSUT2

MPRRPSGGGGGAGPAAAAVRKVPLRKLLRAASVACGVQFGWALQLSLLTPYVQELGIPHAFASLVWLCGPLSGLLVQPLVGHLSDRIAPAAAPSSPPAPLPSLPQFSPSDSPPTSAESSAIPSPRAPPASAPSSSTSSASGSSTSATTLHRDPAGPSSPTSPRMTQGGLG

>OsSUT3

MAVDMELDGGGDGKGKAPPQISLSGLFLACMVAGGVQYGWALQLSLLTPYVQTLGIPHALTSVMWLCGPIAGLIVQPCVGLYSDKCTSSLGRRRPFILTGCIIICISVIVIGFSSDIGYALGDTTEDCKVYRGPRYHAAAAFILGFWLLDFSNNTVQGPARALMADLSGRHGPSAANAIFCSWMALGNILGYSSGSTNDWHKWFPFLMTRACCEACANLKAAFLVAVVFLGLSTAVTMVFAREVALDPVAAAKRNEGEASGLLAVFKGMKNLPVGMPSVLIVTGLTWLSWFPFILFDTDWMGREIYHGRPDGSPAEVTAFQEGVRQGAFGLLLNSIVLGISSFLIEPMCRRLGARAVWVMSSAVVCVAMAAVSVLSAWSLGDFGGSVQDAARAPAEEGGVRASALALFVFLGLPFAVLCSVPFAVTAQLAASRGGGQGLCTGVLNISIVVPQMAIALGAGPWDELFGEGNIPAFAMASVFAAAAAAAGVVLLPKVSVRSVSMAGGH

>OsSUT4

MPRRPSGGGGGAGPAAAAVRKVPLRKLLRAASVACGVQFGWALQLSLLTPYVQELGIPHAFASLVWLCGPLSGLLVQPLVGHLSDRIAPAASPLGRRRPFIAAGAASIAAAVLTVGFSADLGRIFGDSITPGSTRLGAIIVYLVGFWLLDVGNNATQGPCRAFLADLTENDPRRTRIANAYFSLFMALGNILGYATGAYSGWYKIFPFTVTPSCSISCANLKSAFLLDIIILVVTTCITVASVQEPQSFGSDEADHPSTEQEAFLWELFGSFRYFTLPVWMVLIVTALTWIGWFPFILFDTDWMGREIYRGSPDDPSITQSYHDGVRMGSFGLMLNSVLLGFTSIVLEKLCRKWGAGLVWGVSNILMALCFVAMLVITYVAKNMDYPPSGVPPTGIVIASLVVFTILGAPLAITYSIPYAMAASRVENLGLGQGLAMGILNLAIVIPQVIVSLGSGPWDQLFGGGNAPAFAVAAAASFIGGLVAILGLPRARIASRRRGHR

>OsSUT5

MEEGRRGDREGKSAAGWTALSTTKTTLEEKRRLQANGSVGGDAGTSGFRRIVRLFFACMVAGGIQYGWALQLSLLSPYSQTLGISHSYVSLTWICGPIAGFVVQPIVGYYSDRCTMKMGRRRPFILVGCLIICISVMIIGFSADIGRHLGDTKEHCSTYTGPRWSAAMVYIVGFWFLDFANNTVQGPARAMMADLSAGHHGPNVGQSIFSLWMAIGSVLGYLSGANGKWHEWFPWLKTAACCDACANLKGAFFTAVLLIVVSMTVTMYLADEMPLDKQDVDTSGGGGCAVFVDLFKSLRNLPPAMFKVLAVTAVTWLSWFPFIQYNTDWMGREIYHGEPQGTAAKADVYDAGVREGAMGLLFCSVALGVTSFVIPKLCRRLTSKVVWSISNFLVFALMAVMVAVGMVSMRGYRPSLAAGLTGPDPTLKAVALVVFALIGIPQAVLFSVPWAVASEVTAEEGGGQGLAIGVLNIAIVVPQLVIALTAGPIDGAFNKGNTPAFGIGGAFAFICGVLALIWLPKTRGVSNAAVVAGGH

>AtSTP1

MPAGGFVVGDGQKAYPGKLTPFVLFTCVVAAMGGLIFGYDIGISGGVTSMPSFLKRFFPSVYRKQQEDASTNQYCQYDSPTLTMFTSSLYLAALISSLVASTVTRKFGRRLSMLFGGILFCAGALINGFAKHVWMLIVGRILLGFGIGFANQAVPLYLSEMAPYKYRGALNIGFQLSITIGILVAEVLNYFFAKIKGGWGWRLSLGGAVVPALIITIGSLVLPDTPNSMIERGQHEEAKTKLRRIRGVDDVSQEFDDLVAASKESQSIEHPWRNLLRRKYRPHLTMAVMIPFFQQLTGINVIMFYAPVLFNTIGFTTDASLMSAVVTGSVNVAATLVSIYGVDRWGRRFLFLEGGTQMLICQAVVAACIGAKFGVDGTPGELPKWYAIVVVTFICIYVAGFAWSWGPLGWLVPSEIFPLEIRSAAQSITVSVNMIFTFIIAQIFLTMLCHLKFGLFLVFAFFVVVMSIFVYIFLPETKGIPIEEMGQVWRSHWYWSRFVEDGEYGNALEMGKNSNQAGTKHV

>AtSTP2

MAVGSMNVEEGTKAFPAKLTGQVFLCCVIAAVGGLMFGYDIGISGGVTSMDTFLLDFFPHVYEKKHRVHENNYCKFDDQLLQLFTSSLYLAGIFASFISSYVSRAFGRKPTIMLASIFFLVGAILNLSAQELGMLIGGRILLGFGIGFGNQTVPLFISEIAPARYRGGLNVMFQFLITIGILAASYVNYLTSTLKNGWRYSLGGAAVPALILLIGSFFIHETPASLIERGKDEKGKQVLRKIRGIEDIELEFNEIKYATEVATKVKSPFKELFTKSENRPPLVCGTLLQFFQQFTGINVVMFYAPVLFQTMGSGDNASLISTVVTNGVNAIATVISLLVVDFAGRRCLLMEGALQMTATQMTIGGILLAHLKLVGPITGHAVPLIVLILICVYVSGFAWSWGPLGWLVPSEIYPLEVRNAGYFCAVAMNMVCTFIIGQFFLSALCRFRSLLFFFFGIMNIIMGLFVVFFLPETKGVPIEEMAEKRWKTHPRWKKYFKD

>AtSTP3

MVAEEARKEAMAKSVSGGKITYFVVASCVMAAMGGVIFGYDIGVSGGVMSMGPFLKRFFPKVYKLQEEDRRRRGNSNNHYCLFNSQLLTSFTSSLYVSGLIATLLASSVTRSWGRKPSIFLGGVSFLAGAALGGSAQNVAMLIIARLLLGVGVGFANQSVPLYLSEMAPAKYRGAISNGFQLCIGIGFLSANVINYETQNIKHGWRISLATAAIPASILTLGSLFLPETPNSIIQTTGDVHKTELMLRRVRGTNDVQDELTDLVEASSGSDTDSNAFLKLLQRKYRPELVMALVIPFFQQVTGINVVAFYAPVLYRTVGFGESGSLMSTLVTGIVGTSSTLLSMLVVDRIGRKTLFLIGGLQMLVSQVTIGVIVMVADVHDGVIKEGYGYAVVVLVCVYVAGFGWSWGPLGWLVPSEIFPLEIRSVAQSVTVAVSFVFTFAVAQSAPPMLCKFRAGIFFFYGGWLVVMTVAVQLFLPETKNVPIEKVVGLWEKHWFWRRMTSKRDIQETTILSH

>AtSTP4

MAGGFVSQTPGVRNYNYKLTPKVFVTCFIGAFGGLIFGYDLGISGGVTSMEPFLEEFFPYVYKKMKSAHENEYCRFDSQLLTLFTSSLYVAALVSSLFASTITRVFGRKWSMFLGGFTFFIGSAFNGFAQNIAMLLIGRILLGFGVGFANQSVPVYLSEMAPPNLRGAFNNGFQVAIIFGIVVATIINYFTAQMKGNIGWRISLGLACVPAVMIMIGALILPDTPNSLIERGYTEEAKEMLQSIRGTNEVDEEFQDLIDASEESKQVKHPWKNIMLPRYRPQLIMTCFIPFFQQLTGINVITFYAPVLFQTLGFGSKASLLSAMVTGIIELLCTFVSVFTVDRFGRRILFLQGGIQMLVSQIAIGAMIGVKFGVAGTGNIGKSDANLIVALICIYVAGFAWSWGPLGWLVPSEISPLEIRSAAQAINVSVNMFFTFLVAQLFLTMLCHMKFGLFFFFAFFVVIMTIFIYLMLPETKNVPIEEMNRVWKAHWFWGKFIPDEAVNMGAAEMQQKSV

>AtSTP5

MAGGGLALDVSSAGNIDAKITAAVVMSCIVAASCGLIFGYDIGISGGVTTMKPFLEKFFPSVLKKASEAKTNVYCVYDSQLLTAFTSSLYVAGLVASLVASRLTAAYGRRTTMILGGFTFLFGALINGLAANIAMLISGRILLGFGVGFTNQAAPVYLSEVAPPRWRGAFNIGFSCFISMGVVAANLINYGTDSHRNGWRISLGLAAVPAAIMTVGCLFISDTPSSLLARGKHDEAHTSLLKLRGVENIADVETELAELVRSSQLAIEARAELFMKTILQRRYRPHLVVAVVIPCFQQLTGITVNAFYAPVLFRSVGFGSGPALIATFILGFVNLGSLLLSTMVIDRFGRRFLFIAGGILMLLCQIAVAVLLAVTVGATGDGEMKKGYAVTVVVLLCIYAAGFGWSWGPLSWLVPSEIFPLKIRPAGQSLSVAVNFAATFALSQTFLATLCDFKYGAFLFYGGWIFTMTIFVIMFLPETKGIPVDSMYQVWEKHWYWQRFTKPTST

>AtSTP6

MAVVVSNANAPAFEAKMTVYVFICVMIAAVGGLIFGYDIGISGGVSAMDDFLKEFFPAVWERKKHVHENNYCKYDNQFLQLFTSSLYLAALVASFVASATCSKLGRRPTMQFASIFFLIGVGLTAGAVNLVMLIIGRLFLGFGVGFGNQAVPLFLSEIAPAQLRGGLNIVFQLMVTIGILIANIVNYFTATVHPYGWRIALGGAGIPAVILLFGSLLIIETPTSLIERNKNEEGKEALRKIRGVDDINDEYESIVHACDIASQVKDPYRKLLKPASRPPFIIGMLLQLFQQFTGINAIMFYAPVLFQTVGFGSDAALLSAVITGSINVLATFVGIYLVDRTGRRFLLLQSSVHMLICQLIIGIILAKDLGVTGTLGRPQALVVVIFVCVYVMGFAWSWGPLGWLIPSETFPLETRSAGFAVAVSCNMFFTFVIAQAFLSMLCGMRSGIFFFFSGWIIVMGLFAFFFIPETKGIAIDDMRESVWKPHWFWKRYMLPEDDHHDIEKRNA

>AtSTP7

MAGGSFGPTGVAKERAEQYQGKVTSYVIIACLVAAIGGSIFGYDIGISGGVTSMDEFLEEFFHTVYEKKKQAHESNYCKYDNQGLAAFTSSLYLAGLVSTLVASPITRNYGRRASIVCGGISFLIGSGLNAGAVNLAMLLAGRIMLGVGIGFGNQAVPLYLSEVAPTHLRGGLNMMFQLATTIGIFTANMVNYGTQQLKPWGWRLSLGLAAFPALLMTLGGYFLPETPNSLVERGLTERGRRVLVKLRGTENVNAELQDMVDASELANSIKHPFRNILQKRHRPQLVMAICMPMFQILTGINSILFYAPVLFQTMGFGGNASLYSSALTGAVLVLSTFISIGLVDRLGRRALLITGGIQMIICQVIVAVILGVKFGDNQELSKGYSVIVVIFICLFVVAFGWSWGPLGWTIPSEIFPLETRSAGQSITVAVNLLFTFIIAQAFLGLLCAFKFGIFLFFAGWVTVMTIFVYFLLPETKGVPIEEMTLLWSKHWFWKKVLPDATNLEDESKNVSV

>AtSTP9

MAGGAFVSEGGGGGNSYEGGVTVFVIMTCIVAAMGGLLFGYDLGISGGVTSMEEFLSKFFPEVDKQMHEARRETAYCKFDNQLLQLFTSSLYLAALASSFVASAVTRKYGRKISMFVGGVAFLIGSLFNAFATNVAMLIVGRLLLGVGVGFANQSTPVYLSEMAPAKIRGALNIGFQMAITIGILIANLINYGTSQMAKNGWRVSLGLAAVPAVIMVIGSFVLPDTPNSMLERGKYEQAREMLQKIRGADNVDEEFQDLCDACEAAKKVDNPWKNIFQQAKYRPALVFCSAIPFFQQITGINVIMFYAPVLFKTLGFADDASLISAVITGAVNVVSTLVSIYAVDRYGRRILFLEGGIQMIVSQIVVGTLIGMKFGTTGSGTLTPATADWILAFICLYVAGFAWSWGPLGWLVPSEICPLEIRPAGQAINVSVNMFFTFLIGQFFLTMLCHMKFGLFYFFGGMVAVMTVFIYFLLPETKGVPIEEMGRVWKQHPFWKRYMPDDAVIGGGEENYVKEV

>AtSTP11

MAGGAFIDESGHGGDYEGRVTAFVMITCIVAAMGGLLFGYDIGISGGVISMEDFLTKFFPDVLRQMQNKRGRETEYCKYDNELLTLFTSSLYLAALFASFLASTITRLFGRKVSMVIGSLAFLSGALLNGLAINLEMLIIGRLFLGVGVGFANQSVPLYLSEMAPAKIRGALNIGFQLAITIGILAANIVNYVTPKLQNGIGWRLSLGLAGVPAVMMLVGCFFLPDTPNSILERGNKEKAKEMLQKIRGTMEVEHEFNELCNACEAAKKVKHPWTNIMQARYRPQLTFCTFIPFFQQLTGINVIMFYAPVLFKTIGFGNDASLISAVITGLVNVLSTIVSIYSVDKFGRRALFLQGGFQMIVTQIAVGSMIGWKFGFNGEGNLSGVDADIILALICLYVAGFAWSWGPLGWLVPSEICPLEIRSAGQSLNVSVNMFFTFFIGQFFLTMLCHMKFGLFYFFAGMVLIMTIFIYFLLPETKGVPIEEMGKVWKEHRYWGKYSNNDDGDDVDDDAYF

>AtSTP12

MPSVGIVIGDGKKEYPGKLTLYVTVTCIVAAMGGLIFGYDIGISGGVTTMDSFQQKFFPSVYEKQKKDHDSNQYCRFDSVSLTLFTSSLYLAALCSSLVASYVTRQFGRKISMLLGGVLFCAGALLNGFATAVWMLIVGRLLLGFGIGFTNQSVPLYLSEMAPYKYRGALNIGFQLSITIGILVANVLNFFFSKISWGWRLSLGGAVVPALIITVGSLILPDTPNSMIERGQFRLAEAKLRKIRGVDDIDDEINDLIIASEASKLVEHPWRNLLQRKYRPHLTMAILIPAFQQLTGINVIMFYAPVLFQTIGFGSDAALISAVVTGLVNVGATVVSIYGVDKWGRRFLFLEGGFQMLISQVAVAAAIGAKFGVDGTPGVLPKWYAIVVVLFICIYVAAFAWSWGPLGWLVPSEIFPLEIRSAAQSITVSVNMIFTFLIAQVFLMMLCHLKFGLFIFFAFFVVVMSIFVYLFLPETRGVPIEEMNRVWRSHWYWSKFVDARRI

>AtSTP13

MTGGGFATSANGVEFEAKITPIVIISCIMAATGGLMFGYDVGVSGGVTSMPDFLEKFFPVVYRKVVAGADKDSNYCKYDNQGLQLFTSSLYLAGLTATFFASYTTRTLGRRLTMLIAGVFFIIGVALNAGAQDLAMLIAGRILLGCGVGFANQAVPLFLSEIAPTRIRGGLNILFQLNVTIGILFANLVNYGTAKIKGGWGWRLSLGLAGIPALLLTVGALLVTETPNSLVERGRLDEGKAVLRRIRGTDNVEPEFADLLEASRLAKEVKHPFRNLLQRRNRPQLVIAVALQIFQQCTGINAIMFYAPVLFSTLGFGSDASLYSAVVTGAVNVLSTLVSIYSVDKVGRRVLLLEAGVQMFFSQVVIAIILGVKVTDTSTNLSKGFAILVVVMICTYVAAFAWSWGPLGWLIPSETFPLETRSAGQSVTVCVNLLFTFIIAQAFLSMLCHFKFGIFIFFSAWVLIMSVFVMFLLPETKNIPIEEMTERVWKKHWFWARFMDDHNDHEFVNGEKSNGKSNGFDPSTRL

>AtSTP14

MAGGALTDEGGLKRAHLYEHRITSYFIFACIVGSMGGSLFGYDLGVSGGVTSMDDFLKEFFPGIYKRKQMHLNETDYCKYDNQILTLFTSSLYFAGLISTFGASYVTRIYGRRGSILVGSVSFFLGGVINAAAKNILMLILGRIFLGIGIGFGNQAVPLYLSEMAPAKIRGTVNQLFQLTTCIGILVANLINYKTEQIHPWGWRLSLGLATVPAILMFLGGLVLPETPNSLVEQGKLEKAKAVLIKVRGTNNIEAEFQDLVEASDAARAVKNPFRNLLARRNRPQLVIGAIGLPAFQQLTGMNSILFYAPVMFQSLGFGGSASLISSTITNAALVVAAIMSMYSADKFGRRFLLLEASVEMFCYMVVVGVTLALKFGEGKELPKSLGLILVVLICLFVLAYGRSWGPMGWLVPSELFPLETRSAGQSVVVCVNLFFTALIAQCFLVSLCHLKYGIFLLFAGLILGMGSFVYFLLPETKQVPIEEVYLLWRQHWLWKKYVEDVDE

>OsMST1

MAGGVIVANDGDGSAVDHGGRLTFSVVITCLVAASGGLIFGYDVGISGGVSTMEPFLRRFFPGVVRRMAEARPGNEYCVYDSQALTAFTSSLYVAGLVASLVASRVTRAMGRQAVMVMGGALFFAGGAVTGFAVNIAMLIVGRMLLGFGVGFTNQAAPLFLAEMAPTRWRGSLTAGFQFFLAVGVVIATVTNYFASRVPWGWRLSLGLAGAPAVVIFLGALFLTDTPSSLVMRGDTARARAALAPGARGWRRTWRRSWKGIVRAVEVARQGEDGAFRRMAARREYRPNLVFAVAMPMFFQLTGVIVISFFSPLVFRTVGFGSNAALMGNVILGAVNLVCLMLSTLVIDRYGRKVLFMVGGAIMIIAQVGVAWIMGAQVGKNGSEAMARPYAVAVVAFTCLHTAGFGWSWGPLGWVIPGEIFPVDIRSAGQAMNVSIGLGLTFVQTQSFLAMLCRFRYGTFAYYAAWVAVMTVFIAVFLPETKGVPLESMATVWARHWYWKRFAREQPKTSADEPTGTY

>OsMST2

MAAATAADVAEDTASVYSGKLTLYVFLTCGVAATGGLIIGYDIGISGGVTSMDTFLGKFFPSVLHQEQTAQGTSQYCKFNSQPLTAFTSSLYLAALVASFFVASFTRALGRKWSMFGGGVSFLAGATLNGAARNVAMLIVGRILLGIGVAFCGLSTPIYLSEMAPPRLRGMLNIGLQLMITVGIFSANLVNYGAAKIRGGWGWRVSLGLAAAPACVIAVGSLFLPDSPSSLINRGRHEQARRVLRRIRGTDEVDDEYGDLVAAASEIEVYSGCSARRRPWRDVLQRRYRPQLAMAVLIPFFQQLTGINVIMFYAPVLFKTIGLGGDASLMSAVITGLVNIVATFVSIATVDSLGRRKLLFQGGCQMLVSQVIIGTLIGVVFGTSGDGNISRALVCIVVFICVYVAGFAWSWGPLGVLLPSEIFPLEVRPAGQSISVAVNMLCTFAVAEAFLPMLCHMRFGLFYFFSGWVLVMTLFVSAFLPETKGVPIEKMTVVWRTHWFWGRFYCNQDADAHVQVANSKV

>OsMST3

MAGGAVVSTGAGKDYPGKLTLFVFFTCVVAATGGLIFGYDIGISGGVTSMDPFLRKFFPEVYRKKQMADKNNQYCKYDNQLLQTFTSSLYLAALVSSFFAATVTRVLGRKWSMFAGGLTFLIGAALNGAAENVAMLIVGRILLGVGVGFANQSVPVYLSEMAPARLRGMLNIGFQLMITIGILAAELINYGTAKIKAGWGWRVSLALAAVPAAIITLGSLFLPDTPNSLIDRGHPEAAERMLRRIRGSDVDVSEEYADLVAASEESKLVQHPWRNILRRKYRAQLTMAICIPFFQQLTGINVIMFYAPVLFDTLGFKSDASLMSAVITGLVNVFATLVSIFTVDRLGRRKLFLQGGAQMVVCQVVVGTLIAVKFGTSGIGDIPKGYAAVVVLFICMYVAGFAWSWGPLGWLVPSEIFPLEIRPAGQSINVSVNMLFTFVIAQAFLTMLCHMKFGLFYFFAGWVVIMTVFIALFLPETKNVPIEEMVLVWKSHWFWRRFIGDHDVHVGANHVSNNKLQP

>OsMST4

MAGGFSVSGSGVEFEAKITPIVIISCIMAATGGLMFGYDVGISGGVTSMDDFLREFFPTVLKKKHEDKESNYCKYDNQGLQLFTSSLYLAGLTATFFASYTTRRLGRRLTMLIAGVFFIVGVIFNGAAQNLAMLIVGRILLGCGVGFANQAVPLFLSEIAPTRIRGGLNILFQLNVTIGILFANLVNYGTAKIHPWGWRLSLSLAGIPAALLTLGALFVVDTPNSLIERGRLEEGKAVLRKIRGTDNVEPEFNEIVEASRVAQEVKHPFRNLLQRRNRPQLVIAVLLQIFQQFTGINAIMFYAPVLFNTLGFKTDASLYSAVITGAVNVLSTLVSVYSADRVGRRMLLLEAGVQMFLSQVAIAVVLGIKVTDRSDNLGHGWAIMVVVMVCTFVSSFAWSWGPLGWLIPSETFPLETRSAGQSVTVCVNLLFTFVIAQAFLSMLCHLKYAIFAFFSAWVVVMSLFVLFFLPETKNIPIEEMTERVWKQHWFWKRFMDDADKHHVVPNGGKSNGATV

>OsMST5

MAGGAMVQTVGGKTYPGKMTAFVFFTCLVASSGGLIFGYDIGISGGVTSMDSFLSEFFPSVYAQAKASKDTNQYCKFDSQLLTLFTSSLYLAALATSFVAAWVTRVFGRKWSMFCGGVTFLAGSALNGAATDVMMLILGRILLGIGVGFANQSVPLYLSEMAPANLRGMLNIGFQLMTTIGILSANLINYATSSIEGGWGWRIGLGLAGVPALIITLGALVLPDTPNSLIARGYAGDAKRVLVKIRGTDDVHDEYDDMVAASEEAASIEHPWRNILHRKYRPQLTIAILIPCFQQLTGINVIMFYAPVLFLTIGFAGDASLMSAVITGLVNMFATVVSIISVDRLGRRVLFLQGGTQMFISQVVVGTLIALQFGVAGVGEMSRSYAILLVLFICMYVAGFAWSWGPLGWLVPSEVFALEIRSAGQSIAVCVNMMLTFVIGQAFLTMLCHLKFGLFYFFAGWMLVMTTFVALFLPETKGVPIEEMNHVWSRHWFWGSYVTAHDVAGAGAGGGGNRRSHNV

>OsMST6

MAGGVVVNNGGGKDYPGKLTMFVLFACIVAATGGLIFGYDIGISGGVTSMNPFLIKFFPSVYRKEQAAEKNQSNQYCKFDSPLLTMFTSSLYLAALVASFFASTVTRVAGRKWSMFGGGVTFLVGAALNGAAKNVLMLILGRVLLGVGVGFANQSVPLYLSEMAPARLRGMLNIGFQLMITIGILCANLINYGTAKIKGGWGWRVSLALAAVPAAIIAVGALFLPDTPNSLIDRGHTDAAKRMLRRVRGTDDIEEEYNDLVAASEESKLVAHPWRNILQRRYRPQLTMAIAIPLFQQLTGINVIMFYAPVLFKTLGFADDASLMSAVITGLVNVFATFVSIVTVDRLGRRKLFLQGGTQMLACQIVVGGLIGAEFGFSGVADIPKAYAAFVVLFICAYVAGFAWSWGPLGWLVPSEIFPLEIRSAGQSINVSVNMLFTFIIAQAFLPMLCRFKFILFFFGAWVVIMTLFVAFFLPETKNVPIEEMVLVWKSHWYWGRFIRDEDVHVGADVEMPAAGNRNGKVDPAKLAN

>OsMST7

MENAGAGDGAPKHYPGKMTVFVFIACLVASSGGLIFGYDIGISGGVTSMDPFLSRFFPSVYAKEKEVVDTNQYCKFDSEPLTLFTSSLYLAALIASLFASVITRKLGRKMTMLGGGFIFLIGAVLNGAAVNVAMLIIGRILLGIGVGFSIQAVPLYLSEMAPAKMRGMLNIIFQLMITVGILFANLINYFTDKIAGGWGWRVSLGLAAVPAVIMTVGSILLPDTPNSLLSRGKENEARTMLRRIRGTEDIGPEYDDLVAASEATKAIENPWRTLLERRYRPQLVMSVLIPTLQQLTGINVVMFYAPVLFKTIGFGGTASLMSAVITGLVNMFATFVSIATVDRFGRRVLFIQGGIQMIIAQFILGTLIAVKFGTAGVANISQGYAIVVVLFICLFVSAFAWSWGPLGWLVPSEIFPLEIRSAAQSVVVVFNMAFTFFIAQIFLMMLCRLKFGLFFFFGAMELIMTGFVLVFLPETKGIPIEEMDRIWGEHWYWSRFVGAGRNRVMQMASTNV

>OsMST8

MAGGAMTDTDGAHKNYPGKMTIFVFLACLVASSGGLIFGYDIGISGGVTSMDSFLIKFFPSVYAKEKEMVETNQYCKFDSELLTLFTSSLYLAALIASLFASVITRKFGRRITMLGGGVIFLVGAILNGAAADVAMLIIGRILLGIGVGFSNQAVPLYLSEMAPARMRGMLNISFQLMITVGILAANLINYFTDKIAGGWGWRVSLGLAAVPAVIMAGGSLFLPDTPNSLLSRGKENEARAMLRRIRGTDDVGPEYDDLVAASEASKAIENPWRTLLERRYRPQLVMSVLIPTLQQLTGINVVMFYAPVLFKTIGFGGTASLMSAVITGLVNMFATFVSIATVDRLGRRKLLLQGGVQMIFAQFILGTLIAVKFGTAGVANISRGYAIVVVLCICVFVSAFAWSWGPLGWLVPSEIFPLEIRSAAQSVVVVFNMAFTFIIAQIFLMMLCHLKFGLFYFFGAMELIMTGFVFFFLPETKGIPIEEMDRIWGKHWYWRRFVGAGAGGKVEITSTV

>AtPMT1

MNSSGVEQGVVIAESEPPRGNRSRYAFACAILASMTSIILGYDIGVMSGASIFIKDDLKLSDVQLEILMGILNIYSLVGSGAAGRTSDWLGRRYTIVLAGAFFFCGALLMGFATNYPFIMVGRFVAGIGVGYAMMIAPVYTAEVAPASSRGFLTSFPEIFINIGILLGYVSNYFFSKLPEHLGWRFMLGVGAVPSVFLAIGVLAMPESPRWLVLQGRLGDAFKVLDKTSNTKEEAISRLDDIKRAVGIPDDMTDDVIVVPNKKSAGKGVWKDLLVRPTPSVRHILIACLGIHFAQQASGIDAVVLYSPTIFSKAGLKSKNDQLLATVAVGVVKTLFIVVGTCVVDRFGRRALLLTSMGGMFLSLTALGTSLTVINRNPGQTLKWAIGLAVTTVMTFVATFSIGAGPVTWVYCSEIFPVRLRAQGASLGVMLNRLMSGIIGMTFLSLSKGLTIGGAFLLFAGVAAAAWVFFFTFLPETRGIPLEEMETLFGSYTANKKNNSMSKDNEVVDGQ

>AtPMT2

MSSSGEERGVVVAESEPPRGNRSRFAFACAILASMTSIILGYDIGVMSGAAIFIKDDLKLSDVQLEILMGILNIYSLIGSGAAGRTSDWIGRRYTIVLAGFFFFCGALLMGFATNYPFIMVGRFVAGIGVGYAMMIAPVYTTEVAPASSRGFLSSFPEIFINIGILLGYVSNYFFAKLPEHIGWRFMLGIGAVPSVFLAIGVLAMPESPRWLVMQGRLGDAFKVLDKTSNTKEEAISRLNDIKRAVGIPDDMTDDVIVVPNKKSAGKGVWKDLLVRPTPSVRHILIACLGIHFSQQASGIDAVVLYSPTIFSRAGLKSKNDQLLATVAVGVVKTLFIVVGTCLVDRFGRRALLLTSMGGMFFSLTALGTSLTVIDRNPGQTLKWAIGLAVTTVMTFVATFSLGAGPVTWVYASEIFPVRLRAQGASLGVMLNRLMSGIIGMTFLSLSKGLTIGGAFLLFAGVAVAAWVFFFTFLPETRGVPLEEIESLFGSYSANKKNNVMSKGKQVVDEQ

>AtPMT3

MVHADGHNFPGSDPNPHMNKFAFGCAIVASIISIIFGYDTGVMSGAQIFIRDDLKINDTQIEVLAGILNLCALVGSLTAGKTSDVIGRRYTIALSAVIFLVGSVLMGYGPNYPVLMVGRCIAGVGVGFALMIAPVYSAEISSASHRGFLTSLPELCISLGILLGYVSNYCFGKLTLKLGWRLMLGIAAFPSLILAFGITRMPESPRWLVMQGRLEEAKKIMVLVSNTEEEAEERFRDILTAAEVDVTEIKEVGGGVKKKNHGKSVWRELVIKPRPAVRLILIAAVGIHFFEHATGIEAVVLYSPRIFKKAGVVSKDKLLLATVGVGLTKAFFIIIATFLLDKVGRRKLLLTSTGGMVFALTSLAVSLTMVQRFGRLAWALSLSIVSTYAFVAFFSIGLGPITWVYSSEIFPLRLRAQGASIGVAVNRIMNATVSMSFLSMTKAITTGGVFFVFAGIAVAAWWFFFFMLPETKGLPLEEMEKLFGGGGPRGDRDGLEIQTKTISIGGFS

>AtPMT4

MMKNLPEVGNGGGSGFPAVSVGNKKNKYQRMDSDAEESQNHREAEARNSRTRKYVMACAFFASLNNVLLGYDVGVMSGAVLFIQQDLKITEVQTEVLIGSLSIISLFGSLAGGRTSDSIGRKWTMALAALVFQTGAAVMAVAPSFEVLMIGRTLAGIGIGLGVMIAPVYIAEISPTVARGFFTSFPEIFINLGILLGYVSNYAFSGLSVHISWRIMLAVGILPSVFIGFALCVIPESPRWLVMKGRVDSAREVLMKTNERDDEAEERLAEIQLAAAHTEGSEDRPVWRELLSPSPVVRKMLIVGFGIQCFQQITGIDATVYYSPEILKEAGIQDETKLLAATVAVGVTKTVFILFATFLIDSVGRKPLLYVSTIGMTLCLFCLSFTLTFLGQGTLGITLALLFVCGNVAFFSIGMGPVCWVLTSEIFPLRLRAQASALGAVGNRVCSGLVAMSFLSVSRAITVGGTFFVFSLVSALSVIFVYVLVPETSGKSLEQIELMFQGGLERKDGEVELGDAERLVRKEQEF

>AtPMT5

MTGATPENRTAPSPPPVKHVPESVLPAKPPKRNNYAFACAILASMTSILLGYDIGVMSGAMIYIKRDLKINDLQIGILAGSLNIYSLIGSCAAGRTSDWIGRRYTIVLAGAIFFAGAILMGLSPNYAFLMFGRFIAGIGVGYALMIAPVYTAEVSPASSRGFLNSFPEVFINAGIMLGYVSNLAFSNLPLKVGWRLMLGIGAVPSVILAIGVLAMPESPRWLVMQGRLGDAKRVLDKTSDSPTEATLRLEDIKHAAGIPADCHDDVVQVSRRNSHGEGVWRELLIRPTPAVRRVMIAAIGIHFFQQASGIDAVVLFSPRIFKTAGLKTDHQQLLATVAVGVVKTSFILVATFLLDRIGRRPLLLTSVGGMVLSLAALGTSLTIIDQSEKKVMWAVVVAIATVMTYVATFSIGAGPITWVYSSEIFPLRLRSQGSSMGVVVNRVTSGVISISFLPMSKAMTTGGAFYLFGGIATVAWVFFYTFLPETQGRMLEDMDELFSGFRWRDSKSKPKGNPEKTVPNPEVEIGSNKQWKEGDTQSS

>AtPMT6

MADQISGEKPAGVNRFALQCAIVASIVSIIFGYDTGVMSGAMVFIEEDLKTNDVQIEVLTGILNLCALVGSLLAGRTSDIIGRRYTIVLASILFMLGSILMGWGPNYPVLLSGRCTAGLGVGFALMVAPVYSAEIATASHRGLLASLPHLCISIGILLGYIVNYFFSKLPMHIGWRLMLGIAAVPSLVLAFGILKMPESPRWLIMQGRLKEGKEILELVSNSPEEAELRFQDIKAAAGIDPKCVDDVVKMEGKKTHGEGVWKELILRPTPAVRRVLLTALGIHFFQHASGIEAVLLYGPRIFKKAGITTKDKLFLVTIGVGIMKTTFIFTATLLLDKVGRRKLLLTSVGGMVIALTMLGFGLTMAQNAGGKLAWALVLSIVAAYSFVAFFSIGLGPITWVYSSEVFPLKLRAQGASLGVAVNRVMNATVSMSFLSLTSAITTGGAFFMFAGVAAVAWNFFFFLLPETKGKSLEEIEALFQRDGDKVRGENGAA

>MdSOT1

MTSILMGYDIGVMSGASIYIEKDLKVTDTQIEIMIGVIEIYSLIGSAMAGKTSDWVGRRYTIVISGAIFFIGAILMGFSTNYTFLMCGRFVAGIGVGYALTIAPVYSAEVSPTSSRGFLTSFPEVFVNIGILLGYLSNYAFSFCPLDLGWRLMLGVGAIPSVGLAVGVLAMPESPRWLVMQGRLGEAKRVLDRTSDSKEESMLRLADIKEAAGIPEECNDDIVQVSGHSHGEGVWKELLVHPTPTVRHILIAAIGFHFFQQASGIDALVLYSPRVFAKAGITSTNQLLLCTVGVGLSKTVFTLVATFFLDRVGRRPLLLTSMAGMVGALVCLGTSLTIVDQHEGVRMTWAVILCLCCVLAYVGFFSSGIGPIAWVYSSEIFPLRLRAQGCGMGVAVNRLMSGILSMTFISLYKAITMGGTFFLYAAIGTVGWIFFFTMLPETQGRTLEDMEVLFGKFHKWRKANKLLEKKKRVAHGDIGTSDPDNRAHLLA

>MdSOT2

MTSILLGYDIGVMSGASLFIKENLKISDVQVEIMNGTLNLYSLIGSALAGRTSDWIGRRYTIVLAGTIFFIGALLMGFAPNYAFLMFGRFVAGVGVGYALMIAPVYTAEISPASFRGFLTSFPEVFVNIGILLGYVSNYAFSKLPIHLNWRIMLGVGAFPSVILAVGVLAMPESPRWLVMQGRLGDAKRVLQKTSESIEECQLRLDDIKEAAGIPKESNDDVVQVSKRSHGEGVWKELLLHPTPAVRHILIAALGIHFFEQSSGIDSVVLYSPRIFEKAGITSYDHKLLATVAVGVVKTICILVATVFLDKFGRRPLLLTSVAGMVFSLSCLGASLTIVDQQHGKIMWAIVLCITMVLLNVAFFSIGLGPITWVYSSEIFPLQLRAQGCSMGVAVNRVTSGVISMTFISLYKAITIGGAFFLYAGIAAVGWVFFYMLYPETQGRTLEDMEVLFGKYHKWREANALLQKAKQVDGDDKGQVH

>MdSOT3

MADRRAEENAVTGGPQNTSIEDFDPPMKPKTSKFAIACALLACTTSVLLGYDIGVMSGASLYIQKNLKISDVQVEVLAGTLNIYSLLGSAFAGRTSDWIGRKYTIVLAGVIFLVGALLMGFATNYAFLMVGRFVAGVGVGYGMMIAPVYTAEISPASFRGFLTSFPEVFVNVGILLGYIANYAFSKLPLHLGWRFMLGVGGVPAIFLTVGVLFMPESPRWLVMQGRLGDAKKVLQRTSESKEECQLRLDDIKEAAGIPPHLNDDIVQVTKSSHGEGVWKELILHPTPAVRHILIAAVGIHFFEQASGIDTVVLYSPRIFAKAGITSSNHKLLATVAVGFTKTVFILVATFFLDKFGRRPLLLTSVGGMVFSLMFLGVGLTIVDHHKGSVPWAIGLCMAMVYFNVAFFSIGLGPITWVYSSEIFPLKLRAQGVSIGVACNRVTSGVVSMTFISLYKAITIGGAFFLYAGISAAAWIFFYTMLPETQGRTLEDTEVLFGKYHRWRKANAMLKERKQVDGDDNNNAQVF

>MdSOT4

MTSILLGYDIGVMSGAAIYIEKDLKVTDTQIEILLGILNLYSLIGSAMAGRTSDWVGRRYTIVISGAIFFTGAILMGLSTNYTFLMCGRFVAGLGVGYALTIAPVYAAEVSPASSRGFLTSFPEVFVNVGILLGYISNYAFSFCALDVGWRLMLGVGAIPSVILAIGVLAMPESPRWLVMQGRLGDARQVLDKTSDSKEESMLRLADIKEAAGIPEDCNDDIVQVTGHSHGEGVWKELFVHPTPTVLHIVIAALGFHFFQQASGIDALVLYSPRVFEKAGITNSNQLLLCTVGVGLSKTVFTFVATFFLDRVGRRPLVLTSMAGMVASLVCLGTSLTIVDQHEGARMTWAVVLCLCCVLAFVGFFSTGIGPIAWVYSSEIFPLRLRAQGCGMGVAVNRVMSGVLSMTFISLYKAITMGGAFFLYAAIGAVGWIFFFTMLPETQGRTLEDMEVLFGKFHKWRKANKLLEKERQVARGDGGTSDPDNRVNLLA

>MdSOT5

MADRTTDDNTVSSQPQKTIADFDPPQKPKRNKYAFACAILASMTSILLGYDIGVMSGAAIYIKDDLKISDVEVEVLLGILNLYSLIGSAAAGRTSDWVGRRYTIVLAGAIFFVGALLMGFATNYSFLMFGRFVAGIGVGYALMIAPVYTAEVSPASSRGFLTSFPEVFINSGILLGYVSNYAFSKLPTHLGWRLMLGVGAIPSIFLAVGVLAMPESPRWLVMQGRLGDATRVLDKTSDSKEESMLRLADIKEAAGIPEHCTDDVVQVPKRSQGQDVWKELLLHPTPAVRHILICAIGIHFFQQASGIDAVVLYSPRIFEKAGITNSDKKLLCTVAVGFVKTVFILVATFFVDKVGRRPLLLASVAGMILSLTGLGLGLTIIDQNHERILWAAVLCLTMVLLYVAFFSIGMGPITWVYSSEIFPLKLRAQGCSLGVAMNRVVSGVLSMTFISLYEAITIGGAFFLYAAIASVAWVFFFTMLPETHGRTLEDMEVLFGKFHKWRKANALLKQKKNASHGDGITNNGQVQLGTRGQVN

>OsPLT1-Os10g0360100

MGEEKQNDERKNKYAVGCSIIGSIISVLMGYDTGVMSGAMLFIKEDLKTNDTQVQVLAGILNVCALVGSLTAGRVSDCVGRRLTISLAACIFLVGSVLMGLAPNFATLLAGRCVAGVGVGYALMIAPVYAAEIASADIRGSLTSLPEICISFGILIGYVANYLLAKLPLVYGWRAMLGLGALPSAALALGVLAMPESPRWLVVQGRAEEALSVLRRVCDRPSEADARLAEIKAAAGLADDDGAAANAGSGGKGVWRELFLHPTPPVRRIVIAALGIHFFQHLTGIEAVVLYSPRIFKAAGIASRNSVLAATIGVGVTKTAFILTAILLVDRIGRRPLYLSSLAGIIASLACLGMGLTVIERSPPHHSPAWAVVLAIATVFTFVASFSIGVGPITWAYSSEVYPLRLRAQGASVGVAINRVMNAGVSMTFVSLYKAITIGGAFFLFAGLAVAAATFFYLLCPETQGKPLEEIEEVFSQGWRARRRASAAAVEMPASGGGGGGGAPMA

>OsPLT2-Os07g0582400

MASAALPEAVAPKKKGNVRFAFACAILASMTSILLGYDIGVMSGASLYIKKDFNISDGKVEVLMGILNLYSLIGSFAAGRTSDWIGRRYTIVFAAVIFFAGAFLMGFAVNYAMLMFGRFVAGIGVGYALMIAPVYTAEVSPASARGFLTSFPEVFINFGILLGYVSNYAFSRLPLNLGWRIMLGIGAAPSVLLALMVLGMPESPRWLVMKGRLADAKVVLEKTSDTAEEAAERLADIKAAAGIPEELDGDVVTVPKRGSGNEKRVWKELILSPTPAMRRILLSGIGIHFFQQASGIDSVVLYSPRVFKSAGITDDKHLLGTTCAVGVTKTLFILVATFFLDRVGRRPLLLSSTGGMILSLIGLGAGLTVVGQHPDAKIPWAIGLSIASTLAYVAFFSIGLGPITWVYSSEIFPLQVRALGCSLGVAANRVTSGVISMTFLSLSKAITIGGSFFLYSGIAALAWVFFYTYLPETRGRTLEEMSKLFGDTAAASESDEPAKEKKKVEMAATN

>OsPLT3-Os07g0582500

TKLTAMAVDPKKKNASYAFTCAILASMASIILGYDIGVMSGASLYIKKDLKITDVQVEILMGILNIYSLVGSFAAGRTADWIGRRFTVVFAAAFFFARALLMGFSGDYATLMVGRFVAGVGVGYAIMIAPVYTAEISPASSRGFLTSFPEVSINLGILLGYVSNYAFARLPLSLGWRVMLGVGAAPSVLLALMVLGMPESPRWLVMKGRLADAKAVLEKIADTPEEASERLADIKAAAGIPDDLDGDVVTVSKKRGGEEGQVWRELVVSPTPAMRRIVLAAVGLHFFQQASGVDSVVLYSPRVFQSAGITGDDQLLGTTCAVGFAKTVFILVAAFLLDRAGRRPLLLTSTGGMVFSLVGLATGLTVVGGSPDAQVPSWAVGLCVASILAYVAFFSVGLGPMSGVYTSEIFPLRARALGFAVAVACNRVTSGVISMTFLSLSSAITIGGSFFLYAAISSLAWVFFFTRLPETRGQTLEEIGKVFGMDDTAMEAEDSAAYRERLLATSP

>OsPLT4-Os03g0197100

MTKDDAVPVAVAPAKRPPINKYAFGCALLASMNSVLLGYDISVMSGAQIFMKEDLKITDTQIEILAGVINIYSLFGSLAAGMTSDWLGRRYTMVLAAAIFFTGALLMGLAPNYAFLMAGRFVAGIGVGYALMIAPVYTAEVAPTSARGFLTSFPEVFNNSGILLGYVSNFAFARLPVHLSWRAMFLVGAVPPIFLGIAVLAMPESPRWLVMRGRIEDARRVLLKTSDSPDEAEDRLLDIKKAVGIPEDASDGEDVVAIVRANKASQGEGVWKELLLNPTRPVRRMLVAGLGLMFIQQATGVDCVVMYSPRVFERAGIKSKTNSLGASMAVGVCKTFFIPIATLLLDRVGRRPLLLASGGGMAIFLFTLATSLLMMDRRPEGEAKALGAISIAAMLSFVASFASGLGPVAWVYTSEIYPVRLRAQAAAIGTGLNRLMSGATTMSFLSLSNAITIAGSFYLYASIAAAGWVFMYFFLPETKGKSLEDTVKLFGKDTDDDDDVDTSRHERKRSTELSAQH

>OsPLT5-Os01g0966900

MTSQLGSARRPSKFQPLQVNKLQWANVILSSTDYFDRRSHTSMAYIYTLQMSYCHQLITLTVDHTHSHQYCLYIHALSSLQRASCCICSLINYICGASLFIKEDMKITDVEVEILLGILNLYSLVGSFAAGRTSDWIGRRLTIILAAVIFFVGAIMMGLSVNYPMLMAGRFVAGIGVGYAFMIAPVYTAEVSPASSRGFLTSFPEVFINFGILLGYVSNYAFSRLRLQLGWRLMLGVGAAPSVALALMVLAMPESPRWLVMKGRLADAKVVLGETSDTAEEAATRLAEIKEAVAIPADLDGDVVAVPKRAGGERRVWKELILSPTPAVRRVLLSALGIHFFQQSSGIDAVVLYSPRVFQSAGITDKNKLLGTTCAVGVTKTLFILVATFTLDRFGRRPLLLASAGGMIATLVTLGLGLTVIGEDATGGGWAIAVSIASILAFVAFFSIGLGPITWVYSSEIFPLHLRALGCALGVGLNRVTSGVISMTFLSLSKAITIGGSFFLYAGVASLAWLFFFTYLPETRGRTLEQMGELFRIHNMAGDDDSAATRPPSPEEEEKITNYVEMAAPSSSSSTATSSHHAK

>OsPLT6-Os11g0637200

MAHAGDATAPLLSSPAKPGDEPRRNMYAFGCATLASMTTILMGYNLALMSGAQLFVREDVGLSDAQIEVLAGSMNVFMLVSILAAGWAADVLGRRGTLVLANAYLMAGALAMSLGATYAALMAARFVTSVGVGFSLVVAPVYNAEISPASARGVLSSLLDMFVNVGILLSYVSNYALAGLPVHVGWRVMYGIGVLPPVFLAAGVLAMPESPRWLAMRGRHADARAVLVRTSDSVEEAELRLEEIKRAVEAPQESAGVGVWRELLLRPSAMVRRIVTCVVGLHFFQQASGIDAIVLYSPLVFKKAGMASNTSVLGATVAVGVVKTCFILVATLLSDRLGRRPLLLASTGGVAVTLTSLALALRVASPSTASAAACVASVMAFVAAFSVGFGPMTATYTAEIMPLRLRAQGASLGMAVNRLTCGVVSMTFISLAGGITMAGCFFLYAGVAAVACVFVYVRLPETRGRSLEDMDVLFAK

>OsPLT7-Os12g0514000

MAPDVEARLLAASSKPATAAAASLPRRNKYPFFCAVLASMTSVLMGYNVAVTSGAQIFMAEDLGVSDAQIEVLSGAINIYSLVGALLAGWTSDRLGRRLTIVLTNGFFLAGPLVMSLAGGYAALMAGRFVAGIGVGYALVIAPVYAAEISPASSRGLLSSLPEIFINGGVMLSYVSNFAFSGLPVHLSWRLMFAAGVVPTVFLAAGVLTMPESPRWLAMKGRRGEARVVLDRTSDTPAEAEQRLQEIEDVVAAAGSVAGNGNGGGGAWKEVATKPGVRRVLAIVLTLQFFQQASGIDSVVLYGPRVLAAAGVASNTLLLGLNVVFGVAKASSILVAMALTDRVGRRPLLLASTGGMTASLLALGSVFAAFGGARDDAAVAAGAAVAVVVAFVCAFSVGIGPLAWVYSSEILPLRLRGQGAGVGTAMNRVVSGVVTMTFISLYGAITMAGAFYLYAAIAAASFVFIYACLPETRGRSLEDMEELFHTK

>OsPLT8-Os04g0529800

MAGAEAANGRNKYAVLDPSDEPEGRRRPSAWERRSKERFVLACAIFASLNAILLGYDVGVMSGAIIYIQKDLHITEFQEEILVGCLSVVSLLGSLSGGRTSDAIGRKWTMALGAIVFQAGAAIMTFAPSFTVLMIGRLLAGVGIGFGAMVSAVYIAEISPAAARGTLTSLPEICINLGILLGYVSNYAFSGLSEHINWRIMLGVGILPSVFIGFALFVIPESPRWLMMEKRVPEARAVLLQISESEAEVEERIAEIEEAANLLKSTKSEDKAVWMELLNPSPAVRRMLYAGCGIQMFQQITGIDATVYYSPTIFRDAGIKSDQELLAATVAVGFTKTVFILVAIFLIDKVGRKPLLYVSTIGMTMCLFVLGIALTLQKHAMGLISPRIGIDLAVFAVCGNVAFFSIGMGPICWVLSSEIFPLRLRAQASALGQVGGRVSSGLVSMSFLSMARIISVAGMFFVFAVISTVSVAFVYFCVPETKGKTLEQIEMMFEGGKEWRGSEIELEDTQHLIQSSKKSVSLG

>OsPLT9-Os04g0678900

MGLPGAEPPAASGGGLPGFFGGKSKYVRMDDVLPQEQEEDGVGGGGGGVRVRRSHSSRRYVFACSVFASLNSVLLGYDVGVMSGCILFIQRDLHINEVQQEVLVGCLSFISLLGSLAGGRTSDAVGRKWTIGLAAIVFQAGAAVMTLAPSFEVLMVGRLLAGVGIGFGVMIAPVYIAEISPAASRGSFTSFPEIFINLGILLGYISNYAFSGLPDHVSWRVMLAVGILPSVSIAFALLVIPESPRWLVMKNRADEAREVLLKVTDSEDEAKERLAEIEAAAAVASAGKYGDKTVWQELTRPSPVIRRMLITGLGIQCFQQITGIDALVYYSPTIFRDAGITTESQLLVATVAVGFFKTAFIALAIVLIDRVGRKPLLYVSTVGMTACLVVLAATLAALAHGSASRSAGIAVAILTVCGDVAFFSVGIGPICWVMSSEIFPLRLRSQAAALGAVMNRVTSGAVAMSFLSVCRAISVAGAFSVFAVISALSVVFVYRYVPETSGKTLEEIELLFGGGGGDGEAARGEVELGDGEHLVHKG

>OsPLT10-Os04g0679000

MGLPGAEPPAASGGGLPGFFGGKSKYVRMDDVLPQEQEEDGVGGGGGGVRVRRSHSSRRYVFACSVFASLNSVLLGYDVGVMSGCILFIQRDLHINEVQQEVLVGCLSFISLLGSLAGGRTSDAVGRKWTIGLAAIVFQAGAAVMTLAPSFEVLMVGRLLAGVGIGFGVMIAPVYIAEISPAASRGSFTSFPEIFINLGILLGYISNYAFSGLPDHVSWRVMLAVGILPSVSIAFALLVIPESPRWLVMKNRADEAREVLLKVTDSEDEAKERLAEIEAAAAVASAGKYGDKTVWQELTRPSPVIRRMLITGLGIQCFQQITGIDALVYYSPTIFRDAGITTESQLLVATVAVGFFKTAFIALAIVLIDRVGRKPLLYVSTNPL

>OsPLT11-Os12g0512100

MKNDDDVESPLLAAAADADHHDVDNSHPAAGSSFALACAVAASLTSIIYGYNRGVMSGAQKFVQLDLGVSDAEIEVLIGATSIYSLVGSLAAGWACDRAGRRRTIALSAAMFLAGSAATAAASGYAALMAGQLVAGVACGFGLVVAPVYIAEIAPPSSRGFLASIPEIAGNSGILLSYIADFALAGLPMSLNWRLMIGIGAVPPLFLAAAALLAMPETPRWLVLHGHHDDARQVLVRTTGGDAALAERRLQEIVSSVKESATKQQLSSAAAAGGGGASTGVWRDILVRPTPAVRRVLFAILGLQFFQQASGVAAMVLYAPRVFNHVGVTSERAVLGATVLLGATKTASIVVPLFLADRLGRRPMLLSSAGGMAVSLLVLGFSLRVSSSSGSGSEWWAAATSVAAAAAFMATFSLGFGPVIWMYGSEILPLRLRAQGTGIGTAANRVMSAAVGMSFISLYEAAGMAGTFYLFAACSAAAWVFVYACLPETKGRSLEEMEALFDAAHPSSPPPAS

>VvPMT1

MGLVGVLESGNGNGVGSKSAYTRMDSEIRSEDGVSPCHPQANTSTNKYVFVCAVFASLNSVLLGYDVGVMSGAILFIQEDLKITEVQEEVLVGCLSIISLLGSLAGGKTSDAIGRKWTIALAAFVFQTGAAVMALAPSFPVLIVGRLLAGVGIGFGVMIAPVYIAEISPAITRGSLTSFPEIFINLGILLGYVSNYAFSGLPVHINWRIMLGVGILPSVFIGLALFIIPESPRWLVMQNRIEEARLVLLKTNVSEIEVEDRLVEIQQAAGIANATRHEQKAVWRELFCPSPSVRRMLITGCGIQCFQQITGIDATVYYSPTIFKDAGIKGNAGLLAATVAVGFTKTMFILVATFLIDRVGRKPLLYVSTIGMTTCLFGLGLTLSLLGNGPLGIKLAILSVCGNVAFFSVGIGPICWVLSSEIFPLRLRAQASALGAVGSRVSSGTIAMSFLSVARAITVAGTFFVFSGISALSIAFVYMCVPETKGKTLEEIEMLFKNEKERRGGELELRDVEHLVQKEPSG

>VvPMT2

MAMDTPEQPTVVESHQPQASEAGGASRKKSSLNKFSLVCALLASTCSILLGYDIGVMSGAVLYIKDEIHISSVQVEILVGSLNVCSLIGSLASGKTSDLIGRRYTIVLAAATFLIGALLMSLAPSYLFLMAGRVVAGIGVGYSLMIAPVYTAELSPAMTRGFLTSLPEVFITFGILLGYIANYALAGLPPKINWRMMLGIAAVPAIVIGISVIGMPESPRWLVMKGRISQAKQILIRTSDDEEEAELRLSEIMREASTTTSAEWSGQGVWMELLCRPSKPIRRILVAAIGMNFFMQASGNDAVVYYSPAVFENAGINDRRQLVGVTIIMGITKTAFVLVSALFLDRYGRRPLLLLGSIGMAVSLGGLALGSKYLEDSEHKPTWAIALCVVAVCADVSFFSIGLGPITWVYSSEIFPTRLRAQGTSMAVSVNRLVSGVVAMTFLSISKAITFGGMFLVFCGVMVIGSIFFYFFIPETKGKSLEDIATLFEDKPLLTDSTRD

>VvPMT3

MDSKEEGKNQTGINKYACACAAVASMISIIFGYDTGVMSGAMLFIKEDLKVNDTQVSVLAGILNVCALVGSLAAGRTSDFLGRRYTIVLASIIFLVGSVLMGYAPNYAVLLTGRCTAGIGVGYALMIAPVYSAEISSPKSRGFLTSLPELGISTGILSGYLANYFMAELPLKLGWRLMLGIAAVPSLGLAIGILKMPESPRWLVMQGRLGDAEKILLRVSNTREEAETRYRDIKIAAGIDEDCNEDVVKPPSNTHGGGVWKELLLRPTPAVRWMLLATIGIHFFEHATGIEAVMLFSPRILKKAGVTSKDKLLLATVGVGITKLTFMALSTLLIDRVGRRPLLLTSTTGMIVALTGLGFGLTMVEHAKERLFWALNLSLVATYTFVAFFNIGVAPVTWVYPAEIFPLKLRAQGASIGVAVNRGTNAAISISFIPIYKAMTIGGAFFMFAGISVVAWIFFYFLLPETKGKPLEEMEMLFTRGGRSKSKNAGIEAQPVG

>VvPMT4

MGLVGVQENGNGEMGLSGVPLGSKNKYRRMDSELTEEDDASQSHHHHVSNSTKKYVFACAVFASLNSVLLGYDVGVMSGAIIFIQEDLKITEVQEEVLVGSLSIVSLLGSLAGGRTSDVIGRKWTMGLAAVIFQTGAAIMTFAPSFQILMVGRLLAGVGIGFGVMIAPVYIAEISPTVARGALTSFPEIFINLGILLGYISNYAFSSFPVHTNWRIMLAVGILPSVFIGFALFIIPESPRWLVMKNRVEEARSVLLKTNENESEVEERLAEIQLAAGTGNAEKHEEKAVWRELLKPSPSLRRMLVTGFGIQCFQQITGIDATVYYSPEIFKGAGIEGNSNLLAATVAVGITKTVFILVAIFLIDKLGRKPLLYISTIGMTVCLFSLGFTLTFLGSGNVGIALAVLSVCGNVAFFSVGIGPVCWVLTSEIFPLRLRAQAAALGAVGNRVCSGLVAMSFLSVSDAITVGGTFFIFSVISALSVAFVYMFVPETKGKSLEQIGLLFQNEHEWQRGEVELGDVEHLVQKE

>VvPMT5

MATGKEKSSVVHGEAQKVVTEFDAPKKNGRNKYAIACTILASMTSILLGYDIGVMSGAAIYIKKDLKISDVEVEILVGILNVYCLFGSAAAGRTSDWIGRRYTIVLASVIFFLGALLMGFATNYVFLMVGRFVAGIGVGYALMIAPVYAAEVSPASSRGFITSFPEVFINAGILFGYISNYAFSKLPTNLGWRFMLGIGAIPSVFLALVVIAMPESPRWLVMQGQLGLAKRVLDKTSDSKEESQLRLADIKAAAGIPEECTDDVVAVPKRSHGEGVWRELLIFPTPSVRRILIAAVGIHFFQQASGIDAVVLYSPRIFEKAGIKDDEHILLATVAVGFVKTCFILVATFLLDRVGRRPLLLTSVAGMIFSLAALGMGLTVIDHSDTKLIWAVALSLCTVLSYVAFFSIGMGPITWVYSSEIFPLRLRAQGCSIGVGVNRVTSGVLSMTFISLYKAITIGGAFFLYSGVALVGWIFFYTWLPETQGRTLEDMEILFTNSSWNKKKSSTNDTSGNSNDHINGQIQLGTNG

>AtERDL6

MSFRDDNEEARNDLRRPFIHTGSWYRMGSRQSSMMGSSQVIRDSSISVLACVLIVALGPIQFGFTCGYSSPTQAAITKDLGLTVSEYSVFGSLSNVGAMVGAIASGQIAEYIGRKGSLMIAAIPNIIGWLCISFAKDTSFLYMGRLLEGFGVGIISYTVPVYIAEIAPQNMRGGLGSVNQLSVTIGIMLAYLLGLFVPWRILAVLGILPCTLLIPGLFFIPESPRWLAKMGMTDEFETSLQVLRGFETDITVEVNEIKRSVASSTKRNTVRFVDLKRRRYYFPLMVGIGLLVLQQLGGINGVLFYSSTIFESAGVTSSNAATFGVGAIQVVATAISTWLVDKAGRRLLLTISSVGMTISLVIVAAAFYLKEFVSPDSDMYSWLSILSVVGVVAMVVFFSLGMGPIPWLIMSEILPVNIKGLAGSIATLANWFFSWLITMTANLLLAWSSGGTFTLYGLVCAFTVVFVTLWVPETKGKTLEELQSLFR

>AtERD6-like1

MESGSMKTPLVNNQEEARSSSSITCGLLLSTSVAVTGSFVYGCAMSYSSPAQSKIMEELGLSVADYSFFTSVMTLGGMITAAFSGKIAAVIGRRQTMWIADVFCIFGWLAVAFAHDKMLLNIGRGFLGFGVGLISYVVPVYIAEITPKAFRGGFSFSNQLLQSFGISLMFFTGNFFHWRTLALLSAIPCGIQMICLFFIPESPRWLAMYGRERELEVTLKRLRGENGDILEEAAEIRETVETSRRESRSGLKDLFNMKNAHPLIIGLGLMLLQQFCGSSAISAYAARIFDTAGFPSDIGTSILAVILVPQSIIVMFAVDRCGRRPLLMSSSIGLCICSFLIGLSYYLQNHGDFQEFCSPILIVGLVGYVLSFGIGLGGLPWVIMSEVFPVNVKITAGSLVTVSNWFFSWIIIFSFNFMMQWSAFGTYFIFAGVSLMSFVFVWTLVPETKGRTLEDIQQSLGQLS

>AtERD6

MERQKSMEKGLLRKSLSIRERKFPNEDAFLESGLSRKSPREVKKPQNDDGECRVTASVFLSTFVAVSGSFCTGCGVGFSSGAQAGITKDLSLSVAEYSMFGSILTLGGLIGAVFSGKVADVLGRKRTMLFCEFFCITGWLCVALAQNAMWLDCGRLLLGIGVGIFSYVIPVYIAEIAPKHVRGSFVFANQLMQNCGISLFFIIGNFIPWRLLTVVGLVPCVFHVFCLFFIPESPRWLAKLGRDKECRSSLQRLRGSDVDISREANTIRDTIDMTENGGETKMSELFQRRYAYPLIIGVGLMFLQQLCGSSGVTYYASSLFNKGGFPSAIGTSVIATIMVPKAMLATVLVDKMGRRTLLMASCSAMGLSALLLSVSYGFQSFGILPELTPIFTCIGVLGHIVSFAMGMGGLPWIIMAEIFPMNVKVSAGTLVTVTNWLFGWIITYTFNFMLEWNASGMFLIFSMVSASSIVFIYFLVPETKGRSLEEIQALLNNSVQ

>AtSFP1 MVMEEGRSIEEGLLQLKNKNDDSECRITACVILSTFVAVCGSFSFGVATGYTSGAETGVMKDLDLSIAQFSAFGSFATLGAAIGALFCGNLAMVIGRRGTMWVSDFLCITGWLSIAFAKEVVLLNFGRIISGIGFGLTSYVVPVYIAEITPKHVRGTFTFSNQLLQNAGLAMIYFCGNFITWRTLALLGALPCFIQVIGLFFVPESPRWLAKVGSDKELENSLFRLRGRDADISREASEIQVMTKMVENDSKSSFSDLFQRKYRYTLVVGIGLMLIQQFSGSAAVISYASTIFRKAGFSVAIGTTMLGIFVIPKAMIGLILVDKWGRRPLLMTSAFGMSMTCMLLGVAFTLQKMQLLSELTPILSFICVMMYIATYAIGLGGLPWVIMSEIFPINIKVTAGSIVTLVSFSSSSIVTYAFNFLFEWSTQGTFFIFAGIGGAALLFIWLLVPETKGLSLEEIQVSLIHQPDERNQT

>AtSFP2

MHKMMVVEKERSIEERLLQLKNQNDDSECRITACVILSTFIAVCGSFSFGVSLGYTSGAEIGIMKDLDLSIAQFSAFASLSTLGAAIGALFSGKMAIILGRRKTMWVSDLLCIIGWFSIAFAKDVMWLNFGRISSGIGLGLISYVVPVYIAEISPKHVRGTFTFTNQLLQNSGLAMVYFSGNFLNWRILALLGALPCFIQVIGLFFVPESPRWLAKVGSDKELENSLLRLRGGNADISREASDIEVMTKMVENDSKSSFCDLFQRKYRYTLVVGIGLMLIQQFSGSSAVLSYASTILRKAGFSVTIGSTLLGLFMIPKAMIGVILVDKWGRRPLLLTSVSGMCITSMLIGVAFTLQKMQLLPELTPVFTFICVTLYIGTYAIGLGGLPWVIMSEIFPMNIKVTAGSIVTLVSWSSSSIVTYAFNFLLEWSTQGTFYVFGAVGGLALLFIWLLVPETKGLSLEEIQASLIREPDRINQS

>AtESL1

MTMSENSRNLEAGLLLRKNQNDINECRITAVVLFSTFVSVCGSFCFGCAAGYSSVAQTGIINDLGLSVAQYSMFGSIMTFGGMIGAIFSGKVADLMGRKGTMWFAQIFCIFGWVAVALAKDSMWLDIGRLSTGFAVGLLSYVIPVYIAEITPKHVRGAFVFANQLMQSCGLSLFYVIGNFVHWRNLALIGLIPCALQVVTLFFIPESPRLLGKWGHEKECRASLQSLRGDDADISEEANTIKETMILFDEGPKSRVMDLFQRRYAPSVVIGVGLMLLQQLSGSSGLMYYVGSVFDKGGFPSSIGSMILAVIMIPKALLGLILVEKMGRRPLLLMNDLYLQASTGGMCFFSLLLSFSFCFRSYGMLDELTPIFTCIGVVGFISSFAVGMGGLPWIIMSEIFPMNVKVSAGTLVTLANWSFGWIVAFAYNFMLEWNASGTFLIFFTICGAGIVFIYAMVPETKGRTLEDIQASLTDFLQ

>AtESL2

MESERLESHLLNKQEEEASSFTSGLLLSTSVVVAGSFCYGCAMSYSSPAQSKIMEELGLSVADYSFFTSVMTLGGMITAVFSGKISALVGRRQTMWISDVCCIFGWLAVAFAHDIIMLNTGRLFLGFGVGLISYVVPVYIAEITPKTFRGGFSYSNQLLQCLGISLMFFTGNFFHWRTLALLSAIPSAFQVICLFFIPESPRWLAMYGQDQELEVSLKKLRGENSDILKEAAEIRETVEISRKESQSGIRDLFHIGNAHSLIIGLGLMLLQQFCGSAAISAYAARIFDKAGFPSDIGTTILAVILIPQSIVVMLTVDRWGRRPLLMISSIGMCICSFFIGLSYYLQKNGEFQKLCSVMLIVGLVGYVSSFGIGLGGLPWVIMSEIFPVNVKITAGSLVTMSNWFFNWIIIYSFNFMIQWSASGTYFIFSGVSLVTIVFIWTLVPETKGRTLEEIQTSLVRLS

>ATZIFL1

MAEEYAECLLEKNFHEDCSGCKVDQMKRLRRGFPFWELFTVWIIVLCTALPISSLFPFLYFMIDDFNIAKKEEDIGFYAGFVGCSFMLGRAFTSVAWGLVADRYGRKPVILIGTASVVVFNTLFGLSLNFWMAIITRFCLGSFNGLLGPIKAYAMEIFRDEYQGLALSAVSTAWGIGLIIGPAIGGFLAQPAKQYPSLFSQDSIFGKFPFFLPCLAISVFAFLVTIVSSRIPETLHNHKFNDDESYDALKDLSDDPESNKVAERNGKSSLLNNWPLISSIIVYCVFSLHDMAYTEIFSLWANSPRKYGGLGYSTADVGSVLAFSGFGLLIFQLSLYSYAERLLGPIIVTRISGSLAMVVLSCYPLIAKLSGLALTVTVTSASVAKSVLGTSAITGLFILQNKAVRQDQRGAANGIAMTAMSLFKAIGPAAAGIIFSWSEKRQGAAFLPGTQMVFFILNVVLALGVVLTFKPFLAETQQ

>ATZIFL2

MADDESRTILLEKNEDCPGCIIDRTKQQQRGVPYLHLSFIWLVSLCTALPISSLFPYIYFMIRDFHIAKQEEDIGFYAGFVGSSFMIGRALTSIFWGKLADRYGRKPIILIGTFSVIIFNTLFGLSTSFWLAISVRFLLGCFNCLLGVIRAYASEVVSEEYNALSLSVVSTSRGIGLILGPAIGGYLAQPAEKYPNIFSQSSVFGRFPYFLPSLVISVYATAVLIACWWLPETLHTRCRIAQGRLNPTELNDDESRGGGLDEQKIINKPSLLRNRPLMAIIIVYCVFSLQEIAYNEIFSLWAVSDRSYGGLSFSSQDVGEVLAISGLGLLVFQLLVYPPLEKSVGLLAVIRLSAVLLIPLLSCYPYIALLSGVTLHLVINCASIIKNALSISLVTGLFIMLNKAVPQNQRGAANGISMTAMSVFKSFGPAGGGVLFSWAQKRQDATFLPGDEMVFLVLNLVQLVGLILTFIPYISQIQ

>OsZIFL1

MVGGGAGEPLRRAWGYHPECPGCRVDRRKEEREGIPYTELSLIWLVTVSSTLPIQSLFPFLYFMIRDLHIAKQEEDIGFYAGFVGASYMFGRALSSVIWGIVADKYGRKPIIIITLISIIIFNTLFGLSSSYWMALTSRGLLGLMCGILGPIKAYATEVCRKEHGHLGLSLVSSSRGIGLIVGPAIGGYLAQPADKYPSIFSEKSIFGRFPYFLPCLCISLLAIVALLASFWLPETLHKHTQDMVLEDSISVEEGLSGPTAEENSAGCLNLFTNWPLMSAIIAYCIFSLQDVAYAEVFSLWAVSDRKYGGLSFSSQDVGSVLAFSGLFLLVFQILVYPSVAKSVEPITLVRIVAILTIPLLSSYPFMAGLSGSILQLIVNCASFLKNAFAVTTITVFNILMNDAVAQDVRASANGVAVTLMSIFKAIAPAIAGAIFAWAQRRQTASFLPGDHLVFFMLNVFTVIGLVSTFRPFYARRSTKHDPITT

>OsZIFL2

MAAGDKAGGDDAAAAAAAPLLVSAAGRRRRCPGCLTEERCKADAGIPYLNFFYIWVVCLCSSLPIQSLFPYLYFMIRDLKVAKEEQDIGFYAGFVGATYFLGRTISAVPWGIFADKYGRKPCIVISILSVIVFNTLFGLSTTYWMAIVTRGLLGLLCGILGPIKAYASEVCRKEHQALGISLVTSSRAIALVVGPAIGGFLSQPAKKYPNLFSEESVFGRFPYFLPCFVISVLAAGACVACIWLPETLHMHHDDKEVIDALEAQDATSDLGETTKESGSGRMGHTKSLLKNWQLMSAITLYCVFSLHDTAYLEIFSLWAVSSRKYRGLSFTSQDVGIVLAISGFGVLVYQLAIYPLLAKYVGPIKPFRYAAVLSILLLSTYPFMANLYGLELKVLINIASLLKNMFAATITIACNILQNTAVTQEQRGVANGISVTLMSIFKAVAPAAAGILFSWAQKHITGLFLPGEQILFLMLNMVSVIGFILTFKPFFALPDMR

>At1G08890.1

MESGSMKTPLVNNQEEARSSSSITCGLLLSTSVAVTGSFVYGCAMSYSSPAQSKIMEELGLSVADYSFFTSVMTLGGMITAAFSGKIAAVIGRRQTMWIADVFCIFGWLAVAFAHDKMLLNIGRGFLGFGVGLISYVVPVYIAEITPKAFRGGFSFSNQLLQSFGISLMFFTGNFFHWRTLALLSAIPCGIQMICLFFIPESPRWLAMYGRERELEVTLKRLRGENGDILEEAAEIRETVETSRRESRSGLKDLFNMKNAHPLIIGLGLMLLQQFCGSSAISAYAARIFDTAGFPSDIGTSILAVILVPQSIIVMFAVDRCGRRPLLMSSSIGLCICSFLIGLSYYLQNHGDFQEFCSPILIVGLVGYVLSFGIGLGGLPWVIMSEVFPVNVKITAGSLVTVSNWFFSWIIIFSFNFMMQWSAFGTYFIFAGVSLMSFVFVWTLVPETKGRTLEDIQQSLGQLS

>At1G08900.1

MESERLESHLLNKQEEEASSFTSGLLLSTSVVVAGSFCYGCAMSYSSPAQSKIMEELGLSVADYSFFTSVMTLGGMITAVFSGKISALVGRRQTMWISDVCCIFGWLAVAFAHDIIMLNTGRLFLGFGVGLISYVVPVYIAEITPKTFRGGFSYSNQLLQCLGISLMFFTGNFFHWRTLALLSAIPSAFQVICLFFIPESPRWLAMYGQDQELEVSLKKLRGENSDILKEAAEIRETVEISRKESQSGIRDLFHIGNAHSLIIGLGLMLLQQFCGSAAISAYAARIFDKAGFPSDIGTTILAVILIPQSIVVMLTVDRWGRRPLLMISSIGMCICSFFIGLSYYLQKNGEFQKLCSVMLIVGLVGYVSSFGIGLGGLPWVIMSEIFPVNVKITAGSLVTMSNWFFNWIIIYSFNFMIQWSASGTYFIFSGVSLVTIVFIWTLVPETKGRTLEEIQTSLVRLS

>At1G08930.1

MERQKSMEKGLLRKSLSIRERKFPNEDAFLESGLSRKSPREVKKPQNDDGECRVTASVFLSTFVAVSGSFCTGCGVGFSSGAQAGITKDLSLSVAEYSMFGSILTLGGLIGAVFSGKVADVLGRKRTMLFCEFFCITGWLCVALAQNAMWLDCGRLLLGIGVGIFSYVIPVYIAEIAPKHVRGSFVFANQLMQNCGISLFFIIGNFIPWRLLTVVGLVPCVFHVFCLFFIPESPRWLAKLGRDKECRSSLQRLRGSDVDISREANTIRDTIDMTENGGETKMSELFQRRYAYPLIIGVGLMFLQQLCGSSGVTYYASSLFNKGGFPSAIGTSVIAtIMVPKAMLAtVLVDKMGRRTLLMASCSAMGLSALLLSVSYGFQSFGILPELTPIFTCIGVLGHIVSFAMGMGGLPWIIMAEIFPMNVKVSAGTLVTVTNWLFGWIITYTFNFMLEWNASGMFLIFSMVSASSIVFIYFLVPETKGRSLEEIQALLNNSVQ

>At1G08920.2

MTMSENSRNLEAGLLLRKNQNDINECRITAVVLFSTFVSVCGSFCFGCAAGYSSVAQTGIINDLGLSVAQYSMFGSIMTFGGMIGAIFSGKVADLMGRKGTMWFAQIFCIFGWVAVALAKDSMWLDIGRLSTGFAVGLLSYVIPVYIAEITPKHVRGAFVFANQLMQSCGLSLFYVIGNFVHWRNLALIGLIPCALQVVTLFFIPESPRLLGKWGHEKECRASLQSLRGDDADISEEANTIKETMILFDEGPKSRVMDLFQRRYAPSVVIGVGLMLLQQLSGSSGLMYYVGSVFDKGGFPSSIGSMILAVIMIPKALLGLILVEKMGRRPLLLMNDLYLQASTGGMCFFSLLLSFSFCFRSYGMLDELTPIFTCIGVVGFISSFAVGMGGLPWIIMSEIFPMNVKVSAGTLVTLANWSFGWIVAFAYNFMLEWNASGTFLIFFTICGAGIVFIYAMVPETKGRTLEDIQASLTDFLQ*

>At3G05400.1

MEGENNMEKGLLLAKKEDSANTTPLLIFSTFIIVSASFTFGAAIGYTADTMSSIMSDLDLSLAQFSLFGSLSTFGGMIGAIFSAKAASAFGHKMTLWVADLFCITGWLAISLAKDIIWLDMGRFLVGIGVGLISYVVPVYIAEITPKHVRGAFTFSNQLLQNCGVAVVYYFGNFLSWRTLAIIGSIPCWIQVIGLFFIPESPRWLAKKGRDKECEEVLQKLRGRKYDIVPEACEIKISVEASKKNSNINIRSLFEKRYAHQLTIGIGLMLLQQLCGTAGISSYGSTLFKLAGFPARIGMMVLSLIVVPKSLMGLILVDRWGRRPLLMTSALGLCLSCITLAVAFGVKDVPGIGKITPIFCFIGILSFTMMFAIGMGALPWIIMSEIFPMDIKVLAGSLVTIANWFTGWIANYAFNFMLVWSPSGTFIISAIICGAtIVFTWCLVPETRRLTLEEIQLSFVNV

>At3G05155.1

MEGENSSIEKGLLLIRKEESANTTPFLVFTTFIIVSASFSFGVALGHTAGTMASIMEDLDLSITQFSVFGSLLTFGGMIGALFSAtIADSFGCKMTLWITEVFCISGWLAIALAKNIIWLDLGRFFVGIGVGLLSYVVPVYIAEITPKTVRGTFTFSNQLLQNCGVAtAYYLGNFMSWRIIALIGILPCLIQLVGLFFVPESPRWLAKEGRDEECEVVLQKLRGDEADIVKETQEILISVEASANISMRSLFKKKYTHQLTIGIGLMLLQQLSGSAGLGYYTGSVFDLAGFPSRIGMTVLSIVVVPKAILGLILVERWGRRPLLMVM

>At3G05160.1

MEEGLLRHENDRDDRRITACVILSTFVAVCSSFSYGCANGYTSGAETAIMKELDLSMAQFSAFGSFLNLGGAVGALFSGQLAVILGRRRTLWACDLFCIFGWLSIAFAKNVLWLDLGRISLGIGVGLTSYVVPVYIAEITPKHVRGAFSASTLLLQNSGISLIYFFGTVINWRVLAVIGALPCFIPVIGIYFIPESPRWLAKIGSVKEVENSLHRLRGKDADVSDEAAEIQVMTKMLEEDSKSSFCDMFQKKYRRTLVVGIGLMLIQQLSGASGITYYSNAIFRKAGFSERLGSMIFGVFVIPKALVGLILVDRWGRRPLLLASAVGMSIGSLLIGVSFTLQEMNLFPEFIPVFVFINILVYFGFFAIGIGGLPWIIMSEIFPINIKVSAGSIVALTSWTTGWFVSYGFNFMFEWSAQGTFYIFAMVGGLSLLFIWMLVPETKGQSLEELQASLTGTT

>At3G05165.1

MVVEEENRSMEEGLLQHQNDRDDRRITACVILSTFVAVCSAFSYGCAAGYTSGAETAIMKELDLSMAQFSAFGSFLNVGGAVGALFSGQLAVILGRRRTLWACDFFCVFGWLSIAFAKNVFWLDLGRISLGIGVGLISYVVPVYIAEITPKHVRGAFTASNQLLQNSGVSLIYFFGTVINWRVMAVIGAIPCILQTIGIFFIPESPRWLAKIRLSKEVESSLHRLRGKDTDVSGEAAEIQVMTKMLEEDSKSSFSDMFQKKYRRTLVVGIGLMLIQQLSGASGITYYSNAIFRKAGFSERLGSMIFGVFVIPKALVGLILVDRWGRRPLLLASAVGMSIGSLLIGVSFTLQQMNVLPELIPIFVFVNILVYFGCFAFGIGGLPWVIMSEIFPINIKVSAGTIVALTSWTSGWFVSYAFNFMFEWSAQGTFYIFAAVGGMSFIFIWMLVPETKGQSLEELQASLTGTS

>AtSGB1

MDSVRRTYTIMRGRHIDKRVPSKEFLSALDKAETAVRLPTGTGKDCGNPSWKRSLPHVLVASLTSLLFGYHLGVVNETLESISIDLGFSGNTIAEGLVVSTCLGGAFIGSLFSGLVADGVGRRRAFQLSALPMIVGASVSASTESLMGMLLGRFLVGIGMGIGPSVTALYVTEVSPAYVRGTYGSSTQIAtCIGLLGSLFAGIPAKDNLGWWRICFWISTVPAAMLAVFMELCVESPQWLFKRGRAAEAEAVFEKLLGGSYVKAAMAELVKSDRGDDADSAKLSELLFGRSFRVVFIGSTLFALQQLSGINAVFYFSSTVFKKAGVPSASANICVGVCNLLGSTVAVVLMDKLGRKVLLIGSFAGMAVSLGLQAIAYTSLPSPFGTLFLSVGGMLLFVLSFAtGAGPVPSLLLSEICPGRLRATALAVCLAVHWVINFFVGLLFLRMLEQLGSVLLNAIFGFFCVVAVIFVQKNVVETKGKSLQEIEISLLSSTQ

>VvSFP1

MDIESVEEGSTTRSLIIKEKPKAHGNGDDDGGRGVGGPESGGSATPVVVLSTLVAICGSYEFGAAVGYSSPAESGIMDDLGLSVTEYSFFGSIMTIGAMIGAVTSGKIADLIGRRGIMRLSALLCGLGWFAIMFSKGAWSLDLGRLSIGCGVGLISYAVPVYIAEISPKNLRGGFTAtHQFMLTIGSALMYFIGTSVNWRILAAIGAIPAVVQLVGLFFIPESPRWLAKIGRENDCEAALRRLRGEKTDISLEAAEIIDYTETMKQLSEGKILDLLQWRYAHSLVVGVGLMILQQFGGCNGIGFYASSIFVSAGFPSKIGTIAMAAVQIPTTIMGIFLMDKSGRRPLLLVSAAGTCLGCFLVGLSFLLQDFNQWKELTSILVLVGMVAFNAFFGIGMAGIPWLIMSEIFPINMKGSAGSLVSLVNWSFSWIITYAFNFMMEWSSAGTFFIFASSGGLTILFVAKLVPETKGRTLEEIQAtMNPFSATE

>VvSFP2

MVKESIEEGQLSSPLIVADKPCENGCSGDGHEEMGGSSAtSVLVLSTLIAVCGSYVFGTAVGYSSPAESGIMDELGLSLAEYSLFGSILTIGAMLGAIVSGRIADLIGRRGAMGFSEVFCIMGWLAVVFSKDAWWLDFGRLSIGCGMGLLSYVVPVYIAEITPKNLRGGFTTVHQLMICCGSSITFLLGTLVNWRILALIGTIPCLIQIVGLPFIPESPRWLARSGRWQDCEDALQRLRGEGAIISQEAAEIKDYSETLQRLSEAtILDLFQWTYARSLIVGVGLMVLQQFGGVNAIVFYASAIFVSAGFSGRVGSIAMVAVQIPMTTLGTILMDKSGRRPLLLASAAGTCLGCFFVGISFLLQGLQGWKELGPIFALLGVLIYDGAFSLGMGGIPWVIMSEIFPINMKGSAGSLVTLVSWLGSWIISYAFNFLMKWSSAGTFFIFSSICGITVLFVAKLVPETKGRTLEEIQASMNPLSAKDIHSIS

>VvSFP3

MESLLVEEKTNGEHCIGPNSDIGGKSSSVTAVVVFSTLVVVCGFVTYGHILGYSSPAESGLMDDQDLSVAGVGTNICFRFFLTVGGIVRAFIGGRIADLIGRRGTMWLAQIFCIMGWLAIVFTKTIQQLMLCCGLSLSFYIGTIITWPVLALIGTVPCLLQLLGLFFIPESPRWLVKLEAALWRLRGENDDIFQEAADIWDFTEAFQHHSEARILDLFQRRYAYSLTFGCGLMVLQQFSG

>VvSFP4

MERGTNVRGHAtRPLLDEERTENFCNRDDGSGSGSSCSDASSFTIVLVFSVAVVFCGSFTLGCALGYSSPAESGIMDDLGLSIAGYSVFSSLLTLGAtISGVTSGRTTDLIGPRGTMWLSEIFCSTGWLAIVFSKDYWWLDLGRLINGIGIGLISYTVPIYISEITPKNIRGLFASAHTLVICCGFSTTFLLGNAVSWRILALIGNAPCILHIIGVFFIPESPRWLAKTGREKELEVALQRLRGENTDISQELAEIKDYTEICQRLSEDRILDLFQWKYAHSLVVGVGLMLLQQLAGSIAIPSYAGSIFESADFSSTFGTTAtAIIQIPAVVIGVLLADRSGRRPLLIVSAAGMCLSCLIIGISFLLQQDHHKWKELTPIMVLIGMVAYLAWYSLGFRGLPWVIISEIYPVNIKGSAGSLVTFIVWSSSTIVVYVFNFMFEWNSAGTFFIFSVFSAAtVLFTKKLVPETKGQTLEEIQASMTQFLQH

>VvSFP5

MERASMEEGLLVEEKTADKYGGIGGSDDGDSSSSITAAVVFSTAVAVCASFTYGCAAGYTSPAESGIIDDLTLSVAEYSFFGSILTIGGILGAAISGKITDLIGRRGTMWFSEIFCTMGWLAIAFAKDHWWLDLGRLSIGFGIGLICYVVPVYIAEITPQNIRGGFTSAHMLMICCGFSLTFFVGTIISWRILALIGAIPCILQVIGLFFIPESPRWLAKVGREEDLVAALRRLRGVNADISQEAAEIQDYTEAFQHLSEARILDLLQRRYAHSLIVGVGLMVLQQFGGSNAIAYYASAIFESADFSSTFGIRAMAILQIPVTLLAVFLIDKCGRRPLLMVSAAGMCLSCLVVALSFLLQQDLHQWKEITPILVLIGILAYTASFSMGVAGLPWVVMSEIFPINIKGSAGSLVTLSNWFCSWITTYTFNFVFEWSSAGTFLLFSIICGATVLFVAKLLPETKGRRLEEIQATMIG

>VvSFP6

MERAGIEEGLLVEEKTNDKYGGIGGSSGDDSYPVTAVAVFSTAVAVCGAFTNGCAVGYSSPAESGIMDDLGLSVAEYSVFGSILTIGGIVGAVICGKITDLFGRRGTMWFSDIFCLMGWLAIALAKDYWWLDLGRLSIGFGIGLICYVVPVYIAEIMPKNIRGGFTSANTLMICCGSSLTFFVGTVVSWRILAVIGAIPCILQVIGLFFIPESPRWLAKVGQEARLEAALQRLRGKNADISQEAAEIREYTEAFQQLSEARILDLFQRRYAHSLIVGVGLMVLQQFGGSNAILYYASSIFESAGFSTTFGTRAMAILQIPVTFLGIVLIDKSGRRPLLMASAAGMCLGCLVVALSFLLQQDLQQMKVLTPIFVLIGVLAYLASLCMGVAGLPWVVMSEIFPINIKGSAGSLVASSNLFCSWITTYTFNFVFAWSSAGTFFLFSIICSAtVLFVAKLLPETKGRRLEEIQATITHFLE

>VvSFP7

VAVCGSFNTGCAAAYSSPAKSGLMEDLGLSVAEAIFGSIWTAGGILGAIISGKTADLIGRRGTMWFADIFCIMGWLLIAFAKDYWWLDLGRLSMGFGVGLISYVAAVYISEISPKSLRGGFTSVSSLMICCGFSLIYFLGTVISWRTLAIIGAVPCTLQTIGLFFIPESPRWLAKVGREKELEAALQRLRGQRANISQEAADIKEYTETFQQLPKAtIVDLFQRRYAHSLIVGVGLMVLAQFSGVTAVQCFASSILESADFSTTLGSRAIAILQIPATAVAILLIDKLGRRPLLMVSAAGMGLSSFLIGLSFLLQDLNLWKEITPILVLIGLLTYSAtYSLGMAGLPWVIMAEIYPINIKGVAGSLVTLSNWFFSWVVTYTFNYIFDWSSTGTFFFYSIISGAtVVFTAKLVPETKGRKLEEIQASMTQSLL

>VvSFP8

MERRNYEEVLVRSSLLVDQESVTNYYHCIGPGGVDGGSSPVTAVVIISTVVAACGSFNTGCAAGYSSPAESGIMEDLGLSLAEYSVFGSLWTVGGIVGALISGTTADLIGRRGTMWFADIFCIMGWLLIAFAKDYWWLDFGRLAtGFGVGLISYVVTVYISEIAPTNIRGGFTSASSLMMCCGFSMIFFVGTVVSWRTLAIIGAVPCVLQAIGLFLVPESPRWLAKVGREKELEASLGRLRGERADITQEAADIIEYTKIFLQFPKAtILDVFQRRYAHSLIVGVGLMVLTQFSGVTAIACFMSSILESADFSTTFGSRAIAILQIPVTAVSVVLIDKSGRRPLLMVSAAGMGLSSLLIGFSFLLQDLNQLKEVTPIVVLIGLLTYSAtNSLGMAGLPWLIMAEIYPINIKGVAGSLVIFSNWFFSWVVTYTFNYMFDWSSTGTFFFYSIISGSTVLFTAKLVPETKGRKLEEIQASMTHFLR

>VvSFP9

MNQLKEVTPIVVLIGLLTYCAtYSMGMAGIPWLIMAEIYPINIKGVAGSVVTLSNWFFSWVVTYTFNYMFDWSSSGTFFFYSIISGAtVLFTAKLVPETKGRKLEEIQASMTHFLP

>VvSFP10

MERRNNEEVLVTSSLLVDQENAtNYYHCIVPGGVDGGSSSVTAAVIFSTVVVVCGTFNTGCAAGYSSPAESGIMEDLGLSVAEYSVFGSLWTAGGIVGALISGRTADLIGRRGAMWFADIFCIMGWLLIAFAKDYWWLDFGRLAIGFAVGLISYVVTVYISEIAPRNIRGGFTSAGSLMMCCGFSMFYFVGTVVSWRTLAIIGAVPCVLQAVGLFFVPESPRWLAKVGREKELEAALWRLRGERADIALEAADIMEYTKTFQQFPKAtILELFRMRYAHSLIVGVGLMVLTQFSGVTAVQCFTSSILESADFSTTFGSRAIAILQIPVMAVSVVLIDKSGRRPLLMVSAAGMGLSSLLIGFSFLMQDMNQLKEVTPIVVLIGLLTYSATYSLGMAGLPWLIMAEIYPINIKGVAGSLVTFSNWLFSWVVTYTFNYMFDWSSAGTFFFYSIISGSTVLFTAKLVPETKGRKLEEIQASMTHFLH

>VvSFP11

MERGNMDEGLTTSSLLLAEKEVRDGSFNCSFIWGYSSPVEHELMDDLGLSLAEYSVFVSIWAFGGIIASLMTGTAIDFIGRRGTMLFADISCIIGWLLIALAKDHWWLDSGRFLTGFAAGHFTYLMLCSGSSLIFFIRTIVSWRTLALIGMVPGLLQFIGLFFVPESPRWLAKLGRDEELEVALQRLRGPRTNVSQEAADIKVSFQLHMSMNENSRILDLFQRRYAHSLIVGVGLIVLRQFSGNNAIWCYASSIFESADFSSGFGTRAIPILQIPAPALGLLIIDKFGRRPILMVSAAGMCFSCFLAGLSFLLQDLKQWKETTPILVLIILLIYFAtFSLGVSGVPWLVVSEMYPINIKGSAGGLVSLANWFFSVVVTYTFNYMFEWSSPGTFFFYSLISAAtVLFTAKLIPETKGRTLEEIQASMTKFLE

>VvSFP12

MGRDSSNGGLITASLLHGEVRDEDGPISINDLEGGDAPPAGGGSSSFFTIAVVFSTLIAVCGSFIFGTAVGYSSPAESGIVNDLGLSTAEYSIFGSILTIGGMIGAVMSGKIADLIGRRGAMWVSEFFCTIGWIAIAFSEGAWLLDIGRLLIGCGIGALSYVVPVYIAEITPKNLRGRFSGLNMLFISCGTSVMYFTGGVVTWRILALIGTIPCLLPLFGLFFVPESPRWLAKVGREKEFEASLQHLRGKDTDISFEASDIKDYTRYLEGLSETRIIDIFQRKYAYCLTVGVGLMIVQEFGGLNGFAFYTSSILDSAGFLSKVGTMAYGLVQIPAtILGVFLFDKIGRRPVLLVSAAGTCLGCFLTGLAFLLQDLHYWKEGTPILALVGVLVFSSSFVFGMGGIPWIIMSEIFPINIKGPAGSLVTFVCWFGSWLVACTFYFLFEWSSAGTFFIFSSICGLGVLFIAKLVPETKGRTLEEIQASITYFLQ

>VvSFP13

MAAKQEVEKGNANITEPLIVQEKQGEAQIKSNNGGLRMVLLSIFVAVCGSFEFGSCAGYSAPAQYGIMNELGLSYSQYSVFGSILSIGAMIGAISSGWIADSIGRKGAMRMSSMVCIAGWITVYLSFGSVSLDSGRFLLGYGIGILSYVIPVFIAEITPKNHRGTLAtANQLFIVTGLFIAFVVGAFVTWRTLALTGILPCMVLLVGLFFIPESPRWLARAGYEREFKAELQKLRGVEADISEEEAEIQEYMVTHQLLPKVGIMVLLDKQNVRSVIVGVGLMVFQQFGGYNGIVFYADQIFVSAGVPPNLGGILYSSLQVIVTAFGASLIDRLGRRPLLMVSAFGLLLGCLLTGISFFLKAHQLAPNLVPILAVTGIMVHIGFYSVGLGPIPWLIMSEIFPLHVKAIAGSLVTLVNWFGAWAVSYTFNFLMNWSSHGTFFGYAFVCAAAIVFIIMVVPETKGQTLEEIQASMNR

>VvSFP14

MAtRQDVEKGNDTITKPLIGQKKEVQIQSNNGGLWVVLLSTLVAVCGSFEFGSCVGYSAPAEYGIMDDLGISYSEYSFFGSILTIGAMIGAITSGQIADFIGRKGAMGMSSMICIAGWFTVYLSFGSFSLYSGRFLLGYGIGVLSYVVPVFIAEITPKNLRGALAtANQLFIVTGLFIAYVIGAIVTWRILALTGIVPCMVLLVGLFFIPESPRWLAKVGNEKEFKLSLQKLRGADADISEEVAEIQEYIVTHELLPKVTIMDLLGKQNIRSVVVGVGLMVFQQFGGINGIVFYAGQIFVSAGELLDCLRFFLFELCFQNKIMLTIRSLIDRLGRRPLLIVSAYGMLLGCLLTGTSFLLKAHQLAtNLVPILAVTGILVYIGFYSVGLGAIPWVIMSEIFPLHIKGTAGSLVTLVNWCGSWAVSYTFNFLMNWSSHGTFFGYAFVCAAAVVFIVMLVPETKGRTLEEIQASMN

>VvSFP15

MSFRDENEDGRDLRKPFLHTGSWYRMGSRQSSMMGSSQVIRDSSVSVVACVLIVALGPIQFGFTSGYSSPTQSAITKDLGLTVSEYSLFGSLSNVGAMVGAIASGQISEYIGRKGSLMIAAIPNIIGWLTISFAKDYSFLYMGRLLEGFGVGIISYTVPVYIAEISPQNLRGGLGSVNQLSVTIGILLAYLLGLFLNWRLLAVLGILPCTILIPGLFFIPESPRWLAKMGMTEDFEASLQVLRGFDTDITFEVNEIKRAVASTSRRTTIRFAELKQRRYWYPLMVGIGLLILQQLSGINGVLFYSTTIFESAGVSSSNLAtCLVGVIQVIAtGITTWLLDKAGRRLLLIISSSVMTISLLVVAVSFFLKDAVSKDSSLYSIMSILAVVGVVAMVVGFSLGMGPIPWVIMSEILPINIKGLAGSVAtLSNWFFSFVVTMTANLLLTWSSGGLSVCLSLSHTHTQYAPCNYMVE

>VvSFP16

MVLLSTFVAVCGSFEFGSCVGYSAPTQSAIREDLDLSLAEYSMFGSILTIGAMLGAITSGLVTDSLGRKGAMRMSASFCITGWLAVYFSMGALLLDMGRFFTGYGIGIFSYVVPIFIAEIAPKSIRGGLTTLNQLMIVCGSSVAFLLGTVTTWRTLALTGLVPCLVLLIGLFFVPESPRWLAKVGREKEFEVALRRLRGKDADVSKEAAEIQVYIENLQSFPKAKMLDLFQTKYIRSLIIGVGLMVFQQFGGINGIGFYVSETFVSAGLSSSKIGTIAYACIQVPITIVGAILMDKSGRRPLLMVSASGTFLGCFLTGASFFLKSNAMLLDWVPVLAIGGVLLYIASFSIGMGAVPWVIMSEIFPINVKGAAGSLVVLVNWLGAWVVSYTFNFLMSWSPTGTFSIYAGFSAMTILFVAKIVPETKGKTLEEIQACIDS

>VvSFP17

MEYHESTLQQDMTEPLMQQDEKGNISFEEDDDLTPRNTSQNGSLGVVWLSTTVAVWGSFQFGCCVHYSSPTQTAIRKDLNLSLAEYSVFASILAIGAMIGGITSGHISNFIGRKGTMRVAAIFCIIGWLAIGFAEGVLLLDIGRMCTGYGIGVFSYVVPVFIAEIAPKDLRGGFTSSNELMIQVGGSITYLLGTVLTWRMLALVGLIPSLMLILGMFFVPESPRWLVMVGQQREFEASLQRLRGKDADISFEASEIQEYTEKLQQMPKIRLLDLFQKRYLHSVIIGVGLMLFKQFGGISAIGSYASAtLELAGFSSGKFGTIVIGLCQIPVTIIAVALMDRCGRRPLLLVSSVGTFLGTFLIGLAFYLKDHELVLKLIPMMVLAGVLIYFWSFASGIGSASWVIMSEIFPLNVKGAAGSLAIWANWFGSWTVSYTFNYLISWSSSGAFFLYSAVSAAAILFVAKLVPETRRRTLEEIQAHMLFSNSHS

>VvSFP18

MEYHESTRQQDMTEPLIRQDEKGSIISEEDDDLKPENPSQKGSPGVEWLSTAIAVWGSFQFGCCVHYTSPTQTAIRKDLNLSLAEYSVFASVLAIGAMIGGLTSGHISDLIGRKGTMRVAAAFCIVGWLAIGFTEGVLLLDLGRMCTGYGIGIFSYVVPVFIAEIAPKDLRGGFTSLNELMIQVGGSITYLLGTVLTWRMLALVGLIPSLMLILGMFFVPESPRWLVMVGQQREFEASLQRLRGKDADISFEASEIQEYTEKLQQMPQIRILDLFQKRYLHSVIIGVGLMLFKQFGGMSAIGSYASAtLELAGFSSGKFGTIVIGLCQIPVTTIAVALMDRCGRRPLLLVSSVGTFLGTFLIGLAFYLKDHELVLKLIPMMVLAGVLIYLWSLASGIGSASWVIMSEIFPLNVKGAAGSLAIWANWFGSWAVSYTFNYLISWSSSGTFFLYSAVSAAAILFVAKLVPETRRRTLEEIQAHMLFSNSHS

>VvSFP19

MGDMNIKQDVEKGEDSTQEEIRKPLMQGQKNLPDAGGSGSEDQTDQSSKEHLWMVYLSTFVAVCGSFEFGSCAGYSSPTQTAIREDLDLSLGEYSVFGSILTFGAMIGAITSGPIADFIGRKGAMRVSSAFCAAGWLAIYFAEGALALDIGRLAtGYGMGVFSYVVPVFIAEIAPKNLRGALTTLNQLMICTGVSVAFIIGTVLTWRVLALTGLVPCAVVLFGLFLIPESPRWLAKTGREKEFEAALQRLRGKDADISLEAAEIQDYIETLQQLPKAKIMDLFQRRYLPSVIIGVGLMFFQQFGGINGICFYVSNIFESAGFSSSVGTITYAILQVIVTAMGAALIDRAGRKPLLLVSASGLVLSCVLAGLSFYFKSHELALKAAPALAVTGILLYIGSFSVGMGAVPWVVMSEIFPINIKGVAGSLAtLMNWFGAWAISYTFNYLMSWSSYGTFIIYGVINALAIVFVVKVVPETKGRTLEQIQATINA

>VvSFP20

MSFREEEDGRDLRKPFLHTGSWYRMGSRQSSIMGSSAQIIRDNSVSVLLCVLIVALGPIQFGFTCGYSSPTQSEIISDLGLSLSEFSIFGSLSNVGAMVGAIASGQIAEYIGRKGSLMIASIPNIIGWLAISFAQDSSFLYMGRLLEGFGVGVISYTVPVYIAEISPQNMRGGLGSVNQLSVTLGILLAYVLGLFVNWRVLAVLGILPCTILIPGLFFIPESPRWLAKMGMTEDFEASLQVLRGFDTDISVEVTEIKRSVASTGKRTTIQFSDLKRKRYWFPLMVGIGLLMLQQLSGINGVLFYSSNIFEAAGISSSDIAtVGLGVIQVIAtGVTTWLVDKAGRRLLLIVSSSGMTLSLLLVSVAFYLKDVISEDSRFYSILGILSLVGLVALVITFSLGVGAIPWVIMSEILPVSIKGLAGSIAtLANWLTSWAVTMTANLLLSWSKGGTFAIYTLMTAFTIVFVTLWVPETKGRTLEEIQRSFR

>VvSFP21

MMQRKKSSILNKIGQPNDWSKCLQAKTGREKELEVALQRLRGENTDISQELAEIKDYTEICQRLSEDRILDLFQWKYAHSLVVGVGLMLLQQLAGSVAIPSYADSIFESADFSSTFGTTAtAIIQIPVVVIGVLLADRSGRRPLLIVSAAGMCLSCLIIGISFLLQVFSKYKFKHFYWKELTPIMAYLAWYSLGFRGLPWVIISEIYPVNIKGSAGSLVTFILWSSSTIVVYVFNFIFEWNSAGTFFIFSVFSAAtVLFTIKLVPETKGRTLEEIQASMTHFLQH

>VvSFP22

LEAALQRLRRKNTDISQESAEIKVAFYILMLMNESRILDLFQLKYAHSLIVGIGLILLQQLVGSSAISSYACSIFESAVHSGRAIAIIQIPAVVLGRLLADRSGRRPLLMVSAGGMCLRFLIVGLSFLLQVSSKSKFKQFYLIYNQAYLSFYSLSLRGLPWLIISEIYPINIKGSAGSLVTFVVWFSSTVTMLVFMFIFVYKHKYFGTFFLFLIFSGATILFTAKLVPETKGRTLEEIQASMTQFPQH

>OsTMT1

MAGAVLVAIAASIGNLLQGWDNATIAGAVLYIKKEFNLQSEPLIEGLIVAMSLIGATIITTFSGAVADSFGRRPMLIASAVLYFVSGLVMLWAPNVYVLLLARLIDGFGIGLAVTLVPLYISETAPTDIRGLLNTLPQFSGSGGMFLSYCMVFGMSLMPQPDWRIMLGVLSIPSLIYFALTIFYLPESPRWLVSKGRMAEAKRVLQGLRGREDVSGEMALLVEGLGVGKDTKIEEYIIGPDDELADEGLAPDPEKIKLYGPEEGLSWVARPVHGQSALGSALGLISRHGSMVSQGKPLVDPVVTLFGSVHEKMPEIMGSMRSTLFPNFGSMFSVAEQQQAKGDWDAESQREGEDYGSDHGGDDIEDSLQSPLISRQATSVEGKEIAAPHGSIMGAVGRSSSLMQGGEAVSSMGIGGGWQLAWKWTEREGADGEKEGGFQRIYLHEEGVTGDRRGSILSLPGGDVPPGGEFVQAAALVSQPALYSKELMEQRLAGPAMVHPSQAVAKGPKWADLFEPGVKHALFVGIGIQILQQFAGINGVLYYTPQILEQAGVGVLLANIGLSSSSASILISGLTTLLMLPSIGIAMRLMDMSGRRFLLLATIPILIVALAILILVNILDVGTMVHASLSTVSVILYFCFFVMGFGPIPNILCAEIFPTTVRGICIAICALTFWIGDIIVTYTLPVMLNAIGLAGVFGIYAVVCILAFLFVFMKVPETKGMPLEVITEFFSVGAKQAKED

>OsINT1

MTIDLSMPGSSGLLDDVGGKKHMNFFSNRYVLALTGAAGIGGFLFGYDTGVISGALLYIRDDFPAVRDNYFLQETIVSMALVGAIIGAAGGGWINDTYGRRKSTLVADMLFALGSLVMCAAGGPYILILGRLLVGLGVGIASVTAPVYIAEAAPSEIRGGLVSTNVLMITGGQFFSYLINLGFTEVPGTWRWMLGVAAVPAILQFVLMLFLPESPRWLFWKDEKAKAISVLEKIYDSDRLEEEVELLASSSMHEFQSDGTGSYLDIFKSKELRLAFFAGAGLQAFQQFTGINTVMYYSPTIVQMAGFTSNKLALLLSLIVAGMNAAGTIVGIYLIDRCGRRRLALTSLAGVVVSLAILAMAFILQSSSDICSNALNGACQGALGWFAVAGLALYIAFFSPGMGPVPWAVNSEIYPEAYRGMCGGMSATVNWVSNLIVAQTFLSIVGLVGTGLTFLIIAGIAVLAFIFVALYVPETKGLSFEQVELLWKERAWGNQGNRQSLLGAAP

>OsINT2

MEGGVHEFDGSTFRECFSLSWRNPYVLRLAFSAGIGGLLFGYDTGVISGALLYIRDDFPSVDKNTWLQEMIVSMAVAGAIIGAAIGGWANDRYGRRTSILVADALFFAGAAVMASATGPAQLVVGRVFVGLGVGTASMTSPLYISEASPARIRGALVSTNGLLITGGQFLSYLINLAFTKAPGTWRWMLGVAAIPAVVQFFLMLFLPESPRWLYRKGREEEAEAILRKIYSAEEVEREKEELKESVEAEARERSSSEKTSLVALLMTTATVRRGLVAGVGLQVFQQLVGINTVMYYSPTIVQLAGFASNQTALALSLVTAGLNAAGSLVSIYFIDRTGRRKLLVISLAGVILSLALLSAVFHEATSHSPPVGAAETAHFHGGALTCPDYSSRSSSSFWDCTRCLKAAAASAGCGFCAAGGGDKLRAGACLAAAAAASNATARDACRGEGREWYTRGCPSRYGWLAMAGLALYIAAFSPGMGTVPWIVNSEVYPLRHRGVCGGAAATANWVSNLAVAQSFLSLTDAIGAAWTFLIFGGLSVAALAFVLVCVPETKGLPIEEVEKMLEGRELRLRFWAKRRHHHGGDGDGGGEKTGGV

>OsINT4

MEGGATLADKAEFKECLRLTWSQPYILQLVFSAGIGGLLFGYDTGVISGALLYIRDDFTAVEKSTVLRETIVSMAVAGAIVGAGFGGWMNDKFGRKPSILIADSLFLAGALIMALAPTPFVIIIGRIFVGLGVGMASMTAPLYISEASPARIRGALVSTNGLLITGGQFMAYLINLAFTKVKGTWRWMLGIAGLPAFIQFILMCMLPESPRWLYRQDRKEEAEAILRKIYPAAEVEEEIDSMRRSIEHEKQLEGSIGEQSLVGKLTKALSSKVVRRGLMAGVIAQVAQQFVGINTVMYYSPTIVQLAGFASNNTAMALSLITSGLNAIGSIVSMFFVDRAGRRRLMIISLVGIVLWLAVLGGTFLGAAHHAPPVSDLETRVFANQTCPEYSPSARWNCMNCLKAQSTCGFCAHGGNKLLPGACLAAGEASRRTCHAGNREFYTEGCPNNFGWLALVALGAYIVSYSPGMGTVPWIVNSEIYPLRFRGVCGGIAAVANWVSNLIVTQTFLSLTKALGTSATFFLFCAVSFFALVVVFFTVPETKGLQFEEVEKMLGEKDYKPWKRYRPDVSSKGRDIGLSVP

>OsITR1

MTIDLSMPGSSGLLDDVGGKKHMNFFSNRYVLALTGAAGIGGFLFGYDTGVISGALLYIRDDFPAVRDNYFLQETIVSMALVGAIIGAAGGGWINDTYGRRKSTLVADMLFALGSLVMCAAGGPYILILGRLLVGLGVGIASVTAPVYIAEAAPSEIRGGLVSTNVLMITGGQFFSYLINLGFTEVPGTWRWMLGVAAVPAILQFVLMLFLPESPRWLFWKDEKAKAISVLEKIYDSDRLEEEVELLASSSMHEFQSDGTGSYLDIFKSKELRLAFFAGAGLQAFQQFTGINTVMYYSPTIVQMAGFTSNKLALLLSLIVAGMNAAGTIVGIYLIDRCGRRRLALTSLAGVVVSLAILAMAFILQSSSDICSNALNGACQGALGWFAVAGLALYIAFFSPGMGPVPWAVNSEIYPEAYRGMCGGMSATVNWVSNLIVAQTFLSIVGLVGTGLTFLIIAGIAVLAFIFVALYVPETKGLSFEQVELLWKERAWGNQGNRQSLLGAAP

>OsITR2

MEGGVHEFDGSTFRECFSLSWRNPYVLRLAFSAGIGGLLFGYDTGVISGALLYIRDDFPSVDKNTWLQEMIVSMAVAGAIIGAAIGGWANDRYGRRTSILVADALFFAGAAVMASATGPAQLVVGRVFVGLGVGTASMTSPLYISEASPARIRGALVSTNGLLITGGQFLSYLINLAFTKAPGTWRWMLGVAAIPAVVQFFLMLFLPESPRWLYRKGREEEAEAILRKIYSAEEVEREKEELKESVEAEARERSSSEKTSLVALLMTTATVRRGLVAGVGLQVFQQLVGINTVMYYSPTIVQLAGFASNQTALALSLVTAGLNAAGSLVSIYFIDRTGRRKLLVISLAGVILSLALLSAVFHEATSHSPPVGAAETAHFHGGALTCPDYSSRSSSSFWDCTRCLKAAAASAGCGFCAAGGGDKLRAGACLAAAAAASNATARDACRGEGREWYTRGCPSRYGWLAMAGLALYIAAFSPGMGTVPWIVNSEVYPLRHRGVCGGAAATANWVSNLAVAQSFLSLTDAIGAAWTFLIFGGLSVAALAFVLVCVPETKGLPIEEVEKMLEGRELRLRFWAKRRHHHGGDGDGGGEKTGGV

>OsITR3

MEGGATLADKAEFKECLRLTWSQPYILQLVFSAGIGGLLFGYDTGVISGALLYIRDDFTAVEKSTVLRETIVSMAVAGAIVGAGFGGWMNDKFGRKPSILIADSLFLAGALIMALAPTPFVIIIGRIFVGLGVGMASMTAPLYISEASPARIRGALVSTNGLLITGGQFMAYLINLAFTKVKGTWRWMLGIAGLPAFIQFILMCMLPESPRWLYRQDRKEEAEAILRKIYPAAEVEEEIDSMRRSIEHEKQLEGSIGEQSLVGKLTKALSSKVVRRGLMAGVIAQVAQQFVGINTVMYYSPTIVQLAGFASNNTAMALSLITSGLNAIGSIVSMFFVDRAGRRRLMIISLVGIVLWLAVLGGTFLGAAHHAPPVSDLETRVFANQTCPEYSPSARWNCMNCLKAQSTCGFCAHGGNKLLPGACLAAGEASRRTCHAGNREFYTEGCPNNFGWLALVALGAYIVSYSPGMGTVPWIVNSEIYPLRFRGVCGGIAAVANWVSNLIVTQTFLSLTKALGTSATFFLFCAVSFFALVVVFFTVPETKGLQFEEVEKMLGEKDYKPWKRYRPDVSSKGRDIGLSVP

>AtTMT1

MKGATLVALAATIGNFLQGWDNATIAGAMVYINKDLNLPTSVQGLVVAMSLIGATVITTCSGPISDWLGRRPMLILSSVMYFVCGLIMLWSPNVYVLCFARLLNGFGAGLAVTLVPVYISETAPPEIRGQLNTLPQFLGSGGMFLSYCMVFTMSLSDSPSWRAMLGVLSIPSLLYLFLTVFYLPESPRWLVSKGRMDEAKRVLQQLCGREDVTDEMALLVEGLDIGGEKTMEDLLVTLEDHEGDDTLETVDEDGQMRLYGTHENQSYLARPVPEQNSSLGLRSRHGSLANQSMILKDPLVNLFGSLHEKMPEAGGNTRSGIFPHFGSMFSTTADAPHGKPAHWEKDIESHYNKDNDDYATDDGAGDDDDSDNDLRSPLMSRQTTSMDKDMIPHPTSGSTLSMRRHSTLMQGNGESSMGIGGGWHMGYRYENDEYKRYYLKEDGAESRRGSIISIPGGPDGGGSYIHASALVSRSVLGPKSVHGSAMVPPEKIAASGPLWSALLEPGVKRALVVGVGIQILQQFSGINGVLYYTPQILERAGVDILLSSLGLSSISASFLISGLTTLLMLPAIVVAMRLMDVSGRRSLLLWTIPVLIVSLVVLVISELIHISKVVNAALSTGCVVLYFCFFVMGYGPIPNILCSEIFPTRVRGLCIAICAMVFWIGDIIVTYSLPVLLSSIGLVGVFSIYAAVCVISWIFVYMKVPETKGMPLEVITDYFAFGAQAQASAPSKDI

>AtTMT2

MSGAVLVAIAAAVGNLLQGWDNATIAGAVLYIKKEFNLESNPSVEGLIVAMSLIGATLITTCSGGVADWLGRRPMLILSSILYFVGSLVMLWSPNVYVLLLGRLLDGFGVGLVVTLVPIYISETAPPEIRGLLNTLPQFTGSGGMFLSYCMVFGMSLMPSPSWRLMLGVLFIPSLVFFFLTVFFLPESPRWLVSKGRMLEAKRVLQRLRGREDVSGEMALLVEGLGIGGETTIEEYIIGPADEVTDDHDIAVDKDQIKLYGAEEGLSWVARPVKGGSTMSVLSRHGSTMSRRQGSLIDPLVTLFGSVHEKMPDTGSMRSALFPHFGSMFSVGGNQPRHEDWDEENLVGEGEDYPSDHGDDSEDDLHSPLISRQTTSMEKDMPHTAHGTLSTFRHGSQVQGAQGEGAGSMGIGGGWQVAWKWTEREDESGQKEGGFKRIYLHQEGFPGSRRGSIVSLPGGDGTGEADFVQASALVSQPALYSKDLLKEHTIGPAMVHPSETTKGSIWHDLHDPGVKRALVVGVGLQILQQFSGINGVLYYTPQILEQAGVGILLSNMGISSSSASLLISALTTFVMLPAIAVAMRLMDLSGRRTLLLTTIPILIASLLVLVISNLVHMNSIVHAVLSTVSVVLYFCFFVMGFGPAPNILCSEIFPTRVRGICIAICALTFWICDIIVTYSLPVLLKSIGLAGVFGMYAIVCCISWVFVFIKVPETKGMPLEVITEFFSVGARQAEAAKNE

>AtTMT3

MRSVVLVALAAAIGNMLQGWDNATIAGAVIYIKKEFHLEKEPKIEGLIVAMSLIGATLITTFSGPVSDKVGRRSMLILSSVLYFLSSIVMFWSPNVYVLLFARLLDGFGIGLAVTLVPIYISETAPSEIRGLLNTFPQFCGSGGMFLSYCLVFGMSLQESPSWRLMLGVLSIPSIAYFVLAAFFLPESPRWLVSKGRMDEARQVLQRLRGREDVSGELALLVEGLGVGKDTSIEEYVIGPDNEENEGGNELPRKDQIKLYGPEDGQSWMAKPVKGQSSLALASRQGSMLPRGGSLMDPLVTLFGSIHENLPSENMNASSRSMLFPNMGSILGMMGRQESQWDPERNNEDSSDQDENLNSPLLSPQTTEPDDYHQRTVGTMHRRQSSLFMANVGETATATSIGGGWQLAWKYNDKVGADGKRVNGGLQRMYIHEETANNNTNNIPFSRRGSLLSFHPEGDGHDQVNGYVQAAALVSQASMMPGGKGETAMLPKEVKDGPGWRELKEPGVKRALMVGVGLQILQQFAGINGVMYYTPQILEETGVSSLLTNLGISAESASLLISALTTLLMLPCILVSMRLMDVTGRRSLMLSTIPILILSLVTLVIGSLVNLGGSINALISTASVTVYLSCFVMGFGAIPNILCSEIFPTSVRGLCITICALTFWICDIIVTYTLPVMLKSIGIAGVFGIYAIVCAVAWVFVYLKVPETKGMPLEVISEFFSVGAKQQDAAASFLSDG

>OsTMT2

MSGAALVAIAASIGNLLQGWDNATIAGAVLYIKKEFKLESEPTVEGLIVAMSLIGATIITTFSGPVSDWIGRRPMLILSSILYFLSSLIMLWSPNVYVLLLARLIDGFGIGLAVTLVPLYISETAPSEIRGLLNTLPQFSGSGGMFLSYCMVFGMSLLPSPDWRIMLGVLAIPSLFFFGLTIFYLPESPRWLVSKGRMAEAKKVLQKLRGREDVSGEMALLVEGLEVGADTSIEEYIIGPAIEPADEHVVDGDKDQITLYGPEEGQSWIARPSKGPSILGSVLSLTSRHGSMVNQSVPLMDPIVTLFGSVHENMPHAGGSMRSTLFPNFGSMFSVTDQHPKVDQWDEENLHRDDEEYASDGAGGDYEDNVHSPLLSRQTTSAEGKDIAHHAHRGSALSMRRRSLLEEGGEAVSSTGIGGGWQLAWKWSEREGEDGKKEGGFKRIYLHQEEVPGSRRGSVISLPGGGDAPEGSEFIHAAALVSQPALYSKDIIEQRMSGPAMIHPSEAAAKGSSWKDLFEPGVRRALLVGVGIQILQQFAGINGVLYYTPQILEQAGVAVLLSNLGLSSASASILISSLTTLLMLPSIGLAMRLMDISGRRFLLLGTIPVLIASLVVLVVSNVIDLGTVAHAALSTISVIIYFCCFVMGFGPIPNILCAEIFPTRVRGICIAICALTFWIGDIIVTYSLPVMLNAIGLAGVFGIYAVVCSIAFVFVFLKVPETKGMPLEVITEFFAVGAKQMQATKA

>OsTMT3

IAGAIMYIKNEFNLQNDPMMEGLILAMSLIGATIITALSGMITNSIGKRPLLSVAAILYSISALIMFQASNEYMLLLARLIYGFGSGLVVTYAPLYISETAPTNMRGLLNTLPQFNGSLGMLLSYIMVFLMSLTLNPNWRIMLGSLSIPSFVFLLLCIFYLPESPVFLVSKGKIEEAKNVMKRLRGTNEVSSEIAFLIQGLTVDQDNYIEDYMIGHNNDEFDDQSISNTETTKLYGHEEGVTWFARPFKGKNVVESDHSPIPNLLDPIVTLFDSIHGNILNTPEFTSSGNMSNDIEQPKTDLESQEDLDTDYEDDLGHPLLFHQGSYMEGIDDACVNGGWHIAWKFVQRENEFGQTQDDFQQIFLQGDILQAGRVSHATALVSTPSFHHSIGPAMVHPSKFNLSTEGQSWSDLLQPGVKQGLIVGVTIQILQQLAGISGILYYTPQILEQAGAGILLKWFNVSSSSSSILTSALTTFTMLPSIGIAMKCMDRYGRRSLLLYTIPMLIVSLIILIVVNVMNLEAIFGAILSTFGVIIYVCCFVMGFGPIPNVLCSELFPPSCRNRCMSICTLTFWIVSIIVTYAFPVMLSSIGLIGVCGIYAVVCIVSFIFVLIKVPETKGMPLAVIANSLAVGARLSVKRNENI

>OsTMT4

MMKSTVFSAVAVSIGYTLLGWDFTTVLEANIHMKKEFGLNNGPSIDGIILAVSVFGSIAITVFSGSLLDWLGRRAALIYSSLLLISGGLLMVWSPNIYILLLARLIVGSGSGLVFTCVPIYISETSPPNMRGSLGTMPQFMFFVGIVFSYCLIFWMTLIPSPNWRIMIGAIFAPSLVYFALLVFYLPESPRWLVSDGKISEARISLQWLRGKDDVSDRSAGEIALIADGMNMITETAVGGHAVGAVRSQSFLGTSTNQMSRHSTFYWHLSDPLVDLLGSIHESMSELGAGRNSYFPVFNSFNIVEQEQTSEQRGNDSLQQSREAYSAEEGNNGDNLQASLLSQVASAETNDINTSFTSEGSSSYLRRHGTSTSGLAQDLISSLHDHDIEEDDEEIHIAALSSQPALGAGLHPFRQQMVRLSETADIKPKWRVLLQPGVRHALCYGMLIQALQQSAGISGLLRYTPQILEQVGVISLFSDIGLDSHSASILISALNASLMLPCITAAMILMDVCGRRVLLLVSIPFLTLSVGAISLSNIVKMGSLPHEILFQLSLTICFCSYVIGLGPIPNILCSEMFPTRARATCASFCSLAFWFGRLLSIYCFPVMLSTIGLSGACAIYAFVCCLVLVFVYLRVPETKGLPLELIAEIFKFSRQECL

>OsTMT5

MRGAVVAAAAAAVGNMLQGWDNATIAGALLYMRRDLPALQAHPALQGLVVATSLIGATIVTTFSGPLSDSRGRRPMLIASALLYSLAGLLMLWSPNVPILLLARLVDGFAIGLAVTLVPVYISETAPPDTRGLLNTLPQLTGSTGMFLSYCMVFLITLAPIPNWRLMLGVLLLPALLYLLLTIFFLPESPRWLVSKGRMKEARTVLQMLRGRQDVSAEMALLVEGLTTGRDTAIEEYVVGPTDEAKVTLYGGMSSGLAPGSMFGSAVTLASRQGSMLDHLKDPVVALLDSLHDMNPPAGGTTDVPNLGSMIGVHDRPPIDWDEENSGDDDGDIAAPLLTMEGEAATSTVGIGGGWQLAWKWTEGVAADGTRQSTVKRMYLHEEQAEGVHAAALVSQSALCTKKEAEAEVEGGWREVLEPGGVRHALVCGVAIQILQQFSGISGVLLYTPQILEQAGVGVLLSRLGLRDDSASILISGVTTLLMLPSIGVAMRLMDVSGRRSLLLWTIPLLVASLAVLVAASVAPMAAAAHAAVCTGSVVVYLCCFVMGFGPIPNILCAEIFPTRVRGLCIAICSLAFWLADIAVTYTLPVMLASLGLAGLFAIYAAVCCVALVFVALRVPETKGLPLEVIIDFFNVGAKGTLPNLHDDDDHY

>OsTMT6

MRGAVLVAVAAAIGNYLQGWDNATIAGAVLYIKREFALETQPAVEGLVVAMSLIGATIITTFSGPVSDLVGRRPMLIASSLLYFAGGLIMLWSPNVYVLLLARLVDGFGVGLAVTLVPVYISETSPPEIRGRLNTLPQFTGSGGMFMSYCMIFAMTLSPSPNWRIMLGVLFVPSLLYLFVTVFYLPESPRWLVSKGRMKEARVVLEMLRGREDVSGEMALLVEGLGTGGDTEIEDYVVGPSEGDAGENEQARDTVTLYGPEQGLSWVAQPVAGGRGSMLGSSLGLQASRHGSMYEQMKDPVVALLGSVHERLPESGGGATGSMRGSTLFPNLGSMLSVNDRPGGSSWDEENVQPGDDDLDEEEEEYLSDDGKDDDDGGGLQAPLLSRQSTDVETKNEPASGQVAMQRHSSIGGGGGVETASTMGIGGGWQLAWKWTENVGPDGVKRGAVKRMYLHEESEAAPGGDSGAAGDAQSTAYVHAAALVSRSMLYTKDVLIGQSPTEPAFANPPEAVAAAASTGPAWRELLEPGVRHALFCGVTIQILQQFSGINGVLYYTPQILDQAGVSVLLASLGLSGDSTSILISGLTTLLMLPSIGVAMRLMDASGRRALLLWTLPVLVASLAVLVVANVVPMAATAHAALSTGSVIVYFCCFVMGFGPIPNILCAEIFPTRVRGLCIAICSLTFWLGDIAVTYSLPVMLSSVGLAGVFSFYAAVCCVALVFVALKVPETKGLPLEVIIEFFNVGAKAGTLPDEEFH

>AtpGlcT-like1

MLGLQRETSSMYKRTSSRDYSPMIDVEDSSGLLENDVDNEMETTNPSWKCSLPHVLVATISSFLFGYHLGVVNEPLESISSDLGFSGDTLAEGLVVSVCLGGAFLGSLFSGGVADGFGRRRAFQICALPMILGAFVSGVSNSLAVMLLGRFLVGTGMGLGPPVAALYVTEVSPAFVRGTYGSFIQIATCLGLMAALFIGIPVHNITGWWRVCFWLSTIPAALLALGMFLCAESPQWLFKQGKIAEAEAEFERLLGGSHVKTAMAELYKLDLDKTDEPDVVSLSELLYGRHSRVVFIGSTLFALQQLSGINAVFYFSSTVFKSAGVPSDLGNIFVGVSNLLGSVIAMVLMDKVGRKLLLLWSFIGMVCSAMALQVGATSSYLPHFSALCLSVGGTLVFVLTFALGAGPVPGLLLPEIFPSRIRAKAMAFCMSVHWVINFFVGLLFLRLLEKLGPRLLYSMFSTFCLMAVMFVKRNVIETKGKTLQEIEISLLAKP

>AtpGlcT-like2

MQSSTYAVKGNAAFAFQRRTFSSDRSTTSTGIRFAGYKSLATTGPLYCSGSEAMGATLARADNGIQSVMSFSSVKARSVRAQASSDGDEEEAIPLRSEGKSSGTVLPFVGVACLGAILFGYHLGVVNGALEYLAKDLGIAENTVLQGWIVSSLLAGATVGSFTGGALADKFGRTRTFQLDAIPLAIGAFLCATAQSVQTMIVGRLLAGIGIGISSAIVPLYISEISPTEIRGALGSVNQLFICIGILAALIAGLPLAANPLWWRTMFGVAVIPSVLLAIGMAFSPESPRWLVQQGKVSEAEKAIKTLYGKERVVELVRDLSASGQGSSEPEAGWFDLFSSRYWKVVSVGAALFLFQQLAGINAVVYYSTSVFRSAGIQSDVAASALVGASNVFGTAVASSLMDKMGRKSLLLTSFGGMALSMLLLSLSFTWKALAAYSGTLAVVGTVLYVLSFSLGAGPVPALLLPEIFASRIRAKAVALSLGMHWISNFVIGLYFLSVVTKFGISSVYLGFAGVCVLAVLYIAGNVVETKGRSLEEIELALTSGA

>OspGlcT-like1

MRWKLKSSAYKRVPSRDAAMDLDVETPAKMADGGAPSWRMSLPHVCVATLTSFLFGYHSGVVNEPLESISTDLGFAGNTLAEGLVVSICLGGAFVGCLFSGSIADGIGRRRAFQLSALPMIIGAAVSALTNSLEGMLLGRFLVGTGMGLGPPVASLYITEVSPPSVRGTYGSFVQIATCLGIVVSLLIGTPVKDIDRWWRVCFWVAAVPATLQALGMEFCAESPQWLYKCGRTTEAEIQFEKLLGPLHVKSAMAELSRSERGDDGENVKYSELFYGRNFNVVFIGTTLFALQQLSGINSVFYFSSTVFRSVGVPPNLANICMGIANLSGSIVAMLLMDKLGRKVLLSGSFLGMAFAMGLQAVGANRHHLGSASVYLSVGGMLLFVLTFSLGAGPVPGLLLPEIFPNKIRAKAMALCMSVHWVVNFFVSLLFLRLLEQLGPQVLYTMFSSACVVAAIFVRRHVVETKGKTLQEIEVSLLQTQ

>OspGlcT-like2

MGTSATAACSRIPPPLLPLSFVFFDPSGRSLISDVYAVALIGGSLFFLQQFAGINGVLYFSSLTFHDVGITSGILASLYVGITNFAGAIVASILMDKQGRKKLLTGSYLGMALAMFLIVYAISFPLDEGVSHGLSITGTLLYIFTFAIGAGPVTGIIIPELSGARTRSKVMGFSFTVHWICNFLVGLYFLELVKKLGVGAVYAGFGGVSFLSALFAYNFIVETKGRSLEEIEMSLSPAPGKRE

>AtVGT1

MGFDPENQSISSVGQVVGDSSSGGITAEKEPLLKENHSPENYSVLAAIPPFLFPALGALLFGYEIGATSCAIMSLKSPTLSGISWYDLSSVDVGIITSGSLYGALIGSIVAFSVADIIGRRKELILAAFLYLVGAIVTVVAPVFSILIIGRVTYGMGIGLTMHAAPMYIAETAPSQIRGRMISLKEFSTVLGMVGGYGIGSLWITVISGWRYMYATILPFPVIMGTGMCWLPASPRWLLLRALQGQGNGENLQQAAIRSLCRLRGSVIADSAAEQVNEILAELSLVGEDKEATFGELFRGKCLKALTIAGGLVLFQQITGQPSVLYYAPSILQTAGFSAAADATRISILLGLLKLVMTGVSVIVIDRVGRRPLLLCGVSGMVISLFLLGSYYMFYKNVPAVAVAALLLYVGCYQLSFGPIGWLMISEIFPLKLRGRGISLAVLVNFGANALVTFAFSPLKELLGAGILFCAFGVICVVSLFFIYYIVPETKGLTLEEIEAKCL

>AtVGT2

MALDPEQQQPISSVSREFGKSSGEISPEREPLIKENHVPENYSVVAAILPFLFPALGGLLYGYEIGATSCATISLQSPSLSGISWYNLSSVDVGLVTSGSLYGALFGSIVAFTIADVIGRRKELILAALLYLVGALVTALAPTYSVLIIGRVIYGVSVGLAMHAAPMYIAETAPSPIRGQLVSLKEFFIVLGMVGGYGIGSLTVNVHSGWRYMYATSVPLAVIMGIGMWWLPASPRWLLLRVIQGKGNVENQREAAIKSLCCLRGPAFVDSAAEQVNEILAELTFVGEDKEVTFGELFQGKCLKALIIGGGLVLFQQITGQPSVLYYAPSILQTAGFSAAGDATRVSILLGLLKLIMTGVAVVVIDRLGRRPLLLGGVGGMVVSLFLLGSYYLFFSASPVVAVVALLLYVGCYQLSFGPIGWLMISEIFPLKLRGRGLSLAVLVNFGANALVTFAFSPLKELLGAGILFCGFGVICVLSLVFIFFIVPETKGLTLEEIEAKCL

>AtVGT3

MAFAVSVQSHFAIRALKRDHFKNPSPRTFCSCFKSRPDSSYLSLKERTCFVSKPGLVTTRYRHIFQVGAETGGEFADSGEVADSLASDAPESFSWSSVILPFIFPALGGLLFGYDIGATSGATLSLQSPALSGTTWFNFSPVQLGLVVSGSLYGALLGSISVYGVADFLGRRRELIIAAVLYLLGSLITGCAPDLNILLVGRLLYGFGIGLAMHGAPLYIAETCPSQIRGTLISLKELFIVLGILLGFSVGSFQIDVVGGWRYMYGFGTPVALLMGLGMWSLPASPRWLLLRAVQGKGQLQEYKEKAMLALSKLRGRPPGDKISEKLVDDAYLSVKTAYEDEKSGGNFLEVFQGPNLKALTIGGGLVLFQQITGQPSVLYYAGSILQTAGFSAAADATRVSVIIGVFKLLMTWVAVAKVDDLGRRPLLIGGVSGIALSLFLLSAYYKFLGGFPLVAVGALLLYVGCYQISFGPISWLMVSEIFPLRTRGRGISLAVLTNFGSNAIVTFAFSPLKEFLGAENLFLLFGGIALVSLLFVILVVPETKGLSLEEIESKILK

>OsVGT1

MAAKVGLFPSSSLTTSPHAIAAAPTNAPRPRGARGLASPGRSAAAMDAFHVSSQAAEPLLRTTGAAAAAAAAATATSNPRLRVRNPDPNPPRPNYSRRRFFYPGARCGHMRRAARRPPAVAPEMARPGRSSRGRPSSFRKIAPLSISLSAELSGTTWFSLSSIQLGLVASGSLYGALGGSLLAYRVADFLGRRIELVTAAALYISGALVTGFAPDFVLLIIGRLLYGIGIGLTGYLVGSLEIDVVGGWRYMFGFGAPLAVIMAIGMWNLPPSPRWLLLRAVQGKASVEDNKKKAIQALRSLRGRFRSDRVLADEIDDTLLSIKAAYAEQESEGNIWKMFEGASLKALIIGGGLVLFQQITGQPSVLYYATSILQTAGFAAASDAAKVSILIGLFKLLMTGVAVFKVDDLGRRPLLIGGIGGIAVSLFLLAAYYKILNSFPFVAVGALLLYVGSYQVSFGPISWLMVSEIFPLRTRGRGISLAVLTNFGSNALVTFAFSPLQEFLGPANIFLLFGAISLLSLVFVILKVPETKGLTLEEIESKLLK

>OsVGT2

MADDPLSNSTTNNKRAEGIQLQHGDCESESTAPLLLAPHESYRLSAAILPFLFPALGGLLYGYDIGATSGATISLKSSTFSGTTWYNLSSLQTGLVVSGSLYGALIGSILAFNIADFLGRRRELILSSVSYLIGALLTAAAPNFPIMVVGRFFYGIGIGLAMHAAPMYIAETAPSQIRGMLISLKEFFIVLGMLLGYIAGSLFVEVVSGWRYMYATSTPLCLIMGIGMCWLPASPRWLLLCAIQGKRNIMESKENATRCLCRLRGQASPDLVSEQVDLILDELSYVDQERQAGFSEIFQGKCLKAMIIGCGLVFFQQVTGQPSVLYYAATILQSAGFSGASDATRVSVLLGLLKLIMTGVAVLVVDRLGRRPLLIGGVSGIAVSLFLLSSYYTLLKDAPYVAVIALLLYVGCYQLSFGPIGWLMISEVFPLRLRGRGLSIAVLVNFASNALVTFAFSPLEDLIGTGILFSAFGVIAVASLVFIFFIVPETKGLTLEEIEASL

>AtSWEET1

MNIAHTIFGVFGNATALFLFLAPSITFKRIIKNKSTEQFSGIPYPMTLLNCLLSAWYGLPFVSKDNTLVSTINGTGAVIETVYVLIFLFYAPKKEKIKIFGIFSCVLAVFATVALVSLFALQGNGRKLFCGLAATVFSIIMYASPLSIMRLVVKTKSVEFMPFFLSLFVFLCGTSWFVYGLIGRDPFVAIPNGFGCALGTLQLILYFIYCGNKGEKSADAQKDEKSVEMKDDEKKQNVVNGKQDLQV

>AtSWEET2

MDVFAFNASLSMCKDVAGIAGNIFAFGLFVSPMPTFRRIMRNKSTEQFSGLPYIYALLNCLICLWYGTPFISHSNAMLMTVNSVGATFQLCYIILFIMHTDKKNKMKMLGLLFVVFAVVGVIVAGSLQIPDQLTRWYFVGFLSCGSLVSMFASPLFVINLVIRTKSVEFMPFYLSLSTFLMSASFLLYGLFNSDAFVYTPNGIGTILGIVQLALYCYYHRNSIEEETKEPLIVSYV

>AtSWEET3

MGDKLRLSIGILGNGASLLLYTAPIVTFSRVFKKKSTEEFSCFPYVMTLFNCLIYTWYGLPIVSHLWENLPLVTINGVGILLESIFIFIYFYYASPKEKIKVGVTFVPVIVGFGLTTAISALVFDDHRHRKSFVGSVGLVASISMYGSPLVVMKKVIETRSVEYMPFYLSFFSFLASSLWLAYGLLSHDLFLASPNMVATPLGILQLILYFKYKNKKDLAPTTMVITKRNDHDDKNKATLEFVVDVDRNSDTNEKNSNNASSI

>AtSWEET4

MVNATVARNIAGICGNVISLFLFLSPIPTFITIYKKKKVEEYKADPYLATVLNCALWVFYGLPMVQPDSLLVITINGTGLAIELVYLAIFFFFSPTSRKVKVGLWLIGEMVFVGIVATCTLLLFHTHNQRSSFVGIFCVIFVSLMYIAPLTIMSKVIKTKSVKYMPFSLSLANFLNGVVWVIYALIKFDLFILIGNGLGTVSGAVQLILYACYYKTTPKDDEDEEDEENLSKVNSQLQLSGNSGQAKRVSA

>AtSWEET5

MTDPHTARTIVGIVGNVISFGLFCAPIPTMVKIWKMKSVSEFKPDPYVATVLNCMMWTFYGLPFVQPDSLLVITINGTGLFMELVYVTIFFVFATSPVRRKITIAMVIEVIFMAVVIFCTMYFLHTTKQRSMLIGILCIVFNVIMYAAPLTVMKLVIKTKSVKYMPFFLSLANFMNGVVWVIYACLKFDPYILIPNGLGSLSGIIQLIIYITYYKTTNWNDDDEDKEKRYSNAGIELGQA

>AtSWEET6

MVHEQLNLIRKIVGILGNFISLCLFLSPTPTFIHIVKKKSVEKYSPLPYLATLLNCLVRALYGLPMVHPDSTLLVTISGIGITIEIVFLTIFFVFCGRQQHRLVISAVLTVQVVFVATLAVLVLTLEHTTDQRTISVGIVSCVFNAMMYASPLSVMKMVIKTKSLEFMPFLLSVVGFLNAGVWTIYGFVPFDPFLAIPNGIGCVFGLVQLILYGTYYKSTKGIMEERKNRLGYVGEVGLSNAIAQTEPENIPYLNKRVSGV

>AtSWEET7

MVFAHLNLLRKIVGIIGNFIALCLFLSPTPTFVRIVKKKSVEEYSPIPYLATLINCLVWVLYGLPTVHPDSTLVITINGTGILIEIVFLTIFFVYCGRQKQRLIISAVIAAETAFIAILAVLVLTLQHTTEKRTMSVGIVCCVFNVMMYASPLSVMKMVIKTKSVEFMPFWLSVAGFLNAGVWTIYALMPFDPFMAIPNGIGCLFGLAQLILYGAYYKSTKRIMAERENQPGYVGLSSAIARTGSEKTANTNQEPNNV

>AtSWEET8

MVDAKQVRFIIGVIGNVISFGLFAAPAKTFWRIFKKKSVEEFSYVPYVATVMNCMLWVFYGLPVVHKDSILVSTINGVGLVIELFYVGVYLMYCGHKKNHRRNILGFLALEVILVVAIILITLFALKGDFVKQTFVGVICDVFNIAMYGAPSLAIIKVVKTKSVEYMPFLLSLVCFVNAGIWTTYSLIFKIDYYVLASNGIGTFLALSQLIVYFMYYKSTPKEKTVKPSEVEISATERV

>AtSWEET9

MFLKVHEIAFLFGLLGNIVSFGVFLSPVPTFYGIYKKKSSKGFQSIPYICALASATLLLYYGIMKTHAYLIISINTFGCFIEISYLFLYILYAPREAKISTLKLIVICNIGGLGLLILLVNLLVPKQHRVSTVGWVCAAYSLAVFASPLSVMRKVIKTKSVEYMPFLLSLSLTLNAVMWFFYGLLIKDKFIAMPNILGFLFGVAQMILYMMYQGSTKTDLPTENQLANKTDVNEVPIVAVELPDVGSDNVEGSVRPMK

>AtSWEET10

MAISQAVLATVFGILGNIISFFVCLAPIPTFVRIYKRKSSEGYQSIPYVISLFSAMLWMYYAMIKKDAMMLITINSFAFVVQIVYISLFFFYAPKKEKTLTVKFVLFVDVLGFGAIFVLTYFIIHANKRVQVLGYICMVFALSVFVAPLGIIRKVIKTKSAEFMPFGLSFFLTLSAVMWFFYGLLLKDMNIALPNVLGFIFGVLQMILFLIYKKPGTKVLEPPGIKLQDISEHVVDVVRLSTMVCNSQMRTLVPQDSADMEATIDIDEKIKGDIEKNKDEKEVFLISKN

>AtSWEET11

MSLFNTENTWAFVFGLLGNLISFAVFLSPVPTFYRIWKKKTTEGFQSIPYVVALFSATLWLYYATQKKDVFLLVTINAFGCFIETIYISMFLAYAPKPARMLTVKMLLLMNFGGFCAILLLCQFLVKGATRAKIIGGICVGFSVCVFAAPLSIIRTVIKTRSVEYMPFSLSLTLTISAVIWLLYGLALKDIYVAFPNVLGFALGALQMILYVVYKYCKTSPHLGEKEVEAAKLPEVSLDMLKLGTVSSPEPISVVRQANKCTCGNDRRAEIEDGQTPKHGKQSSSAAAT

>AtSWEET12

MALFDTHNTWAFVFGLLGNLISFAVFLSPVPTFYRICKKKTTEGFQSIPYVVALFSAMLWLYYATQKKDVFLLVTINSFGCFIETIYISIFVAFASKKARMLTVKLLLLMNFGGFCLILLLCQFLAKGTTRAKIIGGICVGFSVCVFAAPLSIIRTVIKTKSVEYMPFSLSLTLTISAVIWLLYGLALKDIYVAFPNVIGFVLGALQMILYVVYKYCKTPSDLVEKELEAAKLPEVSIDMVKLGTLTSPEPVAITVVRSVNTCNCNDRNAEIENGQGVRNSAATT

>AtSWEET13

MALTNNLWAFVFGILGNIISFVVFLAPVPTFVRICKKKSTEGFQSLPYVSALFSAMLWIYYAMQKDGTAFLLITINAFGCVIETIYIVLFVSYANKKTRISTLKVLGLLNFLGFAAIVLVCELLTKGSTREKVLGGICVGFSVSVFAAPLSIMRVVVRTRSVEFMPFSLSLFLTISAVTWLFYGLAIKDFYVALPNVLGAFLGAVQMILYIIFKYYKTPVAQKTDKSKDVSDHSIDIAKLTTVIPGAVLDSAVHQPPALHNVPETKIQLTEVKSQNMTDPKDQINKDVQKQSQV

>AtSWEET14

MVLTHNVLAVTFGVLGNIISFIVFLAPVPTFVRICKKKSIEGFESLPYVSALFSAMLWIYYALQKDGAGFLLITINAVGCFIETIYIILFITYANKKARISTLKVLGLLNFLGFAAIILVCELLTKGSNREKVLGGICVGFSVCVFAAPLSIMRVVIRTKSVEFMPFSLSLFLTISAITWLFYGLAIKDFYVALPNILGAFLGAVQMILYVIFKYYKTPLVVDETEKPKTVSDHSINMVKLSSTPASGDLTVQPQTNPDVSHPIKTHGGDLEDQMDKKMPN

>AtSWEET15

MGVMINHHFLAFIFGILGNVISFLVFLAPVPTFYRIYKRKSTESFQSLPYQVSLFSCMLWLYYALIKKDAFLLITINSFGCVVETLYIAMFFAYATREKRISAMKLFIAMNVAFFSLILMVTHFVVKTPPLQVSVLGWICVAISVSVFAAPLMIVARVIKTKSVEYMPFTLSFFLTISAVMWFAYGLFLNDICIAIPNVVGFVLGLLQMVLYLVYRNSNEKPEKINSSEQQLKSIVVMSPLGVSEVHPVVTESVDPLSEAVHHEDLSKVTKVEEPSIENGKCYVEATRPETV

>AtSWEET16

MADLSFYVGVIGNVISVLVFLSPVETFWRIVQRRSTEEYECFPYICTLMSSSLWTYYGIVTPGEYLVSTVNGFGALAESIYVLIFLFFVPKSRFLKTVVVVLALNVCFPVIAIAGTRTLFGDANSRSSSMGFICATLNIIMYGSPLSAIKTVVTTRSVQFMPFWLSFFLFLNGAIWGVYALLLHDMFLLVPNGMGFFLGIMQLLIYAYYRNAEPIVEDEEGLIPNQPLLA

>AtSWEET17

MAEASFYIGVIGNVISVLVFLSPVETFWKIVKRRSTEEYKSLPYICTLLGSSLWTYYGIVTPGEYLVSTVNGFGALVETIYVSLFLFYAPRHLKLKTVDVEAMLNVFFPIAAIVATRSAFEDEKMRSQSIGFISAGLNIIMYGSPLSAMKTVVTTKSVKYMPFWLSFFLFLNGAIWAVYALLQHDVFLLVPNGVGFVFGTMQLILYGIYRNAKPVGLSNGLSEIAQDEEEGLTSRVEPLLS

>OsSWEET1a

MEHIARFFFGVSGNVIALFLFLSPVVTFWRIIKKRSTEDFSGVPYNMTLLNCLLSAWYGLPFVSPNNILVTTINGTGSVIEAIYVVIFLIFAERKARLKMMGLLGLVTSIFTMVVLVSLLALHGQGRKLFCGLAATIFSICMYASPLSIMRLVIKTKSVEFMPFLLSLSVFLCGTSWFIYGLLGRDPFIAIPNGCGSFLGLMQLILYAIYRNHKGATPAAAAGKGDAADEVEDAKKAAAAVEMADAKTNKVVADDADADADGKSADDKVASQV

>OsSWEET1b

MEDLAKFLFGVSGNVIALFLFLSPVPTFWRIIRRKSTEDFSGVPYNMTLINCLLSAWYGLPFVSPNNILVSTINGAGAVIETAYVVVFLVFASTHKTRLRTLGLAAAVASVFAAVALVSLLALHGQHRKLLCGVAATVCSICMYASPLSIMRLVIKTKSVEYMPFLMSLAVFLCGTSWFIYGLLGRDPFVTIPNGCGSFLGAVQLVLYAIYRNNKGAGGGSGGKQAGDDDVEMAEGRNNKVADGGAADDDSTAGGKAGTEV

>OsSWEET2a

MMNALGLSVAATSTGSPFHDVCCYGAGIAGNIFALVLFISPLPTFKRIVRNGSTEQFSAMPYIYSLLNCLICLWYGLPFVSYGVVLVATVNSIGALFQLAYTATFIAFADAKNRVKVSSLLVMVFGVFALIVYVSLALFDHQTRQLFVGYLSVASLIFMFASPLSIINLVIRTKSVEYMPFYLSLSMFLMSVSFFAYGVLLHDFFIYIPNGIGTVLGVIQLVLYGYFRKGSREDSLPLLVTHT

>OsSWEET2b

MDSLYDISCFAAGLAGNIFALALFLSPVTTFKRILKAKSTERFDGLPYLFSLLNCLICLWYGLPWVADGRLLVATVNGIGAVFQLAYICLFIFYADSRKTRMKIIGLLVLVVCGFALVSHASVFFFDQPLRQQFVGAVSMASLISMFASPLAVMGVVIRSESVEFMPFYLSLSTFLMSASFALYGLLLRDFFIYFPNGLGLILGAMQLALYAYYSRKWRGQDSSAPLLLA

>OsSWEET3a

MFPDIRFIVGIIGSVACMLLYSAPILTFKRVIKKASVEEFSCIPYILALFSCLTYSWYGFPVVSYGWENMTVCSISSLGVLFEGTFISIYVWFAPRGKKKQVMLMASLILAVFCMTVFFSSFSIHNHHIRKVFVGSVGLVSSISMYGSPLVAMKQVIRTKSVEFMPFYLSLFTLFTSLTWMAYGVIGRDPFIATPNCIGSIMGILQLVVYCIYSKCKEAPKVLHDIEQANVVKIPTSHVDTKGHNP

>OsSWEET3b

MVSNTIRVAVGILGNAASMLLYAAPILTFRRVIKKGSVEEFSCVPYILALFNCLLYTWYGLPVVSSGWENSTVSSINGLGILLEIAFISIYTWFAPRERKKFVLRMVLPVLAFFALTAIFSSFLFHTHGLRKVFVGSIGLVASISMYSSPMVAAKQVITTKSVEFMPFYLSLFSFLSSALWMIYGLLGKDLFIASPNFIGCPMGILQLVLYCIYRKSHKEAEKLHDIDQENGLKVVTTHEKITGREPEAQRD

>OsSWEET4

MVSPDTIRTAIGVVGNGTALVLFLSPVPTFIRIWKKGSVEQYSAVPYVATLLNCMMWVLYGLPAVHPHSMLVITINGTGMAIELTYIALFLAFSLGAVRRRVLLLLAAEVAFVAAVAALVLNLAHTHERRSMIVGILCVLFGTGMYAAPLSVMKMVIQTKSVEYMPLFLSLASLVNGICWTAYALIRFDLYITIPNGLGVMFAVAQLILYAIYYKSTQQIIEARKRKEADHVAMTDVVVDSAKNNPSSGAAAAAANGRY

>OsSWEET5

MVMNPDAVRNVVGIIGNLISFGLFLSPLPTFVTIVKKKDVEEFVPDPYLATFLNCALWVFYGLPFIHPNSILVVTINGTGLLIEIAYLAIYFAYAPKPKRCRMLGVLTVELVFLAAVAAGVLLGAHTYDKRSLIVGTLCVFFGTLMYAAPLTIMKQVIATKSVEYMPFTLSLVSFINGICWTIYAFIRFDILITIPNGMGTLLGAAQLILYFCYYDGSTAKNKGALELPKDGDSSAV

>OsSWEET6a

MISPDAARNVVGIIGNVISFGLFLAPVPTFWRICKRKDVEEFKADPYLATLLNCMLWVFYGIPVVHPNSILVVTINGIGLLVEGTYLLIFFLYSPNKKRLRMCAVLGVELVFMLAVILGVLLGAHTHEKRSMIVGILCVFFGSIMYFSPLTIMGKVIKTKSVEYMPFFLSLVCFLNGVCWTAYALIRFDIYVTIPNGLGALFGAIQLILYACYYRTTPKKTKAAKDVEMPSVVVSGTGAAAAAGGGNTGGGSVSVTVER

>OsSWEET6b

MISPDAARNVVGIIGNVISFGLFLSPVPTFWRICKRKDVEQFKADPYLATLLNCMLWVFYGIPIVHPNSILVVTINGIGLIVEGTYLFIFFLYSPNKKRLRMLAVLGVELVFMLAVILGVLLSAHTHKKRSMIVGILCVFFGSIMYFSPLTIMGKVIKTKSVEYMPFFLSLVCFLNGVCWTAYALIRFDIYVTIPNGLGAIFGAIQLILYACYYRTTPKKTKAAKDVEMPSVISGPGAAATASGGSVVSVTVER

>OsSWEET7a

MVSPDMIRNVVGIVGNVISFGLFLSPVPTFWQIIKNKNKNKKKMEVVLAAEALFMAAVALGVLLGVHTHQRRSLIVGILCVIFDTIMYSSPLTVMSQVVKTKSVEYMPLLLSVVSFLNGLYWTSYTLIRFDIFITIPNGLGVLFAAVQLILYVIYYRTTPKKQNKNLELPTVTPVAKDTSVGPISKDNDLNGSTASHVTIDITIQP

>OsSWEET7b

MVSPDLIRNMVGIVGNIISFGLFLSPVPTFYRIIKNKDVQDFKADPYLATLLNCMLWVFYGLPIVHPNSILVVTINGIGLVIEAVYLTIFFLFSDKKNKKKMGVVLATEALFMAAVVLGVLLGAHTHQRRSLIVGILCVIFGTIMYSSPLTIMSQVVKTKSVEYMPLLLSVEAESYERKVPEILPRQAVEGKGRGAARVLLNCDILLLNCDILSHFCFHSISDQSEISLPENELQALLLSIMFNLFEITSRSLREESNHNSQKHRSVSLATQQMTVANANEVQSLTSTTPALAKEHPLQVQPTKNFESNSTEFVIDIEGPYDAEDITGHTTDKTKFILVNYSNSSEEHKSQDPTQDESDNIPNKSTNVSNIYHAF

>OsSWEET7c

MVSPDLIRNVVGIVGNVISFGLFLSPVPIFWRIIKNKNVQNFKADPILVVTINGISLVIEAVYLTIFFLFSDKKNKKKMGVVLATEALFMAAVAVGVLLGAHTHQRRSLIVGILCVIFGTIMYSSPLTIMVVKTKSVEYMPLLLSVVSFLNGLCWTLYALIRFDIFITIPNGLGVLFAIMQLILYAIYYRTTPKKQDKNLELPTVAPIAKDTSIVAPVSNDDDVNGSTASHATINITIEP

>OsSWEET7d

MVSPDLIRNVVGIVGNAISFGLFLSPVLTFWRIIKEKDMKYFKADPYLATLLNCMLWVFYGLPIVHPNSILVVTINGIGLVIEAVYLTIFFLFSNKKN

>OsSWEET11

MAGGFLSMANPAVTLSGVAGNIISFLVFLAPVATFLQVYKKKSTGGYSSVPYVVALFSSVLWIFYALVKTNSRPLLTINAFGCGVEAAYIVLYLVYAPRRARLRTLAFFLLLDVAAFALIVVTTLYLVPKPHQVKFLGSVCLAFSMAVFVAPLSIIFKVIKTKSVEFMPIGLSVCLTLSAVAWFCYGLFTKDPYVMYPNVGGFFFSCVQMGLYFWYRKPRNTAVLPTTSDSMSPISAAAAATQRVIELPAGTHAFTILSVSPIPILGVHKVEVVAAEQAADGVAAAAAADKELLQNKPEVIEITAAV

>OsSWEET12

MVQALVFAVGIVGNILSFLVILAPVPTFYRVYKKKSTESFQSVPYAVALLSAMLWLYYALLTSDLLLLSINSIGCLVESLYLTVYLLYAPRQAMAFTLKLVCAMNLALFAAVVAALQLLVKATDRRVTLAGGIGASFALAVFVAPLTIIRQVIRTKSVEFMPFWLSFFLTLSAVVWFFYGLLMKDFFVATPNVLGLLFGLAQMVLYVVYKNPKKNSAVSEAAAAQQVEVKDQQQLQMQLQASPAVAPLDVDADADADLEAAAPATPQRPADDDAIDHRSVVVDIPPPPQPPPALPAVEVA

>OsSWEET13

MAGLSLQHPWAFAFGLLGNLISFTTYLAPIPTFYRIYKSKSTEGFQSVPYVVALFSAMLWIFYALIKSNEALLITINAAGCVIETIYIVMYLAYAPKKAKVFTTKILLLLNVGVFGVILLLTLLLSHGEQRVVSLGWVCVAFSVSVFVAPLSIIKRVIQSRSVEYMPFSLSLTLTLSAVVWFLYGLLIKDKYVALPNILGFTFGVVQMGLYVFYMNATPVAGEGKEGKGKLAAAEELPVVVNVGKLAAATPDRSTGAVHVHPVPRSCAAEAAAAEPEVLVDIPPPPPPRAVEVAAV

>OsSWEET14

MAGMSLQHPWAFAFGLLGNIISFMTYLAPLPTFYRIYKSKSTQGFQSVPYVVALFSAMLWIYYALLKSDECLLITINSAGCVIETIYIAVYLVYAPKKAKMFTAKLLLLVNVGVFGLILLLTLLLSAGDRRIVVLGWVCVGFSVSVFVAPLSIIRLVVRTKSVEFMPFSLSFSLTISAVVWFLYGLLIKDKYVALPNVLGFSFGVIQMGLYAMYRNSTPKAVLTKEVEAATATGDDDHSAAGVKEHVVNIAKLSAAVDVVKTREVHPVDVESPPAEAPPEEDDKAAAATAAAVAGAGEKKVAA

>OsSWEET15

MAFMSMERSTWAFTFGILGNLISLMVFLSPLPTFYRVYRKKSTEGFQSTPYVVTLFSCMLWMYYAFVKSGAELLVTINGVGCVIETVYLAMYLAYAPKSARMLTAKMLLGLNIGLFGVIALVTLLLSRGELRVHVLGWICVAVSLSVFAAPLSIIRLVIRTKSVEFMPFSLSFFLVLSAVIWFLYGLLKKDVFVALPNVLGFVFGVAQMALYMAYRSKKPLVASSSSAVVAAGLEIKLPEHVKEVQAVAKGAVAAAPEGRISCGAEVHPIDDVMPSEVVEVKVDDEETNRTDEMAGDGDHAMVRTEQIIKPDMAIVVEV

>OsSWEET16

MADPSFFVGIVGNVISILVFASPIATFRRIVRSKSTEEFRWLPYVTTLLSTSLWTFYGLHKPGGLLIVTVNGSGAALEAIYVTLYLAYAPRETKAKMVKVVLAVNVGALAAVVAVALVALHGGVRLFVVGVLCAALTIGMYAAPMAAMRTVVKTRSVEYMPFSLSFFLFLNGGVWSVYSLLVKDYFIGIPNAIGFALGTAQLALYMAYRRTKKPAGKGGDDDEDDEEAQGVARLMGHQVEMAQQRRDQQLRKGLSLSLPKPAAPLHGGLDRIIKSFSTTPIELHSILHQHHGGHHHHHRFDTVPDDDDEAVAAGGTTPATTAGPGDRH

>OsSWEET2bL

MWPSFFLPPLSLLSLSLPFFPFCRHAGWQQERAAAGGEAGAAAVGGRAARSSGGALRRRGSCARRESKRRRKRTPTLDTAQPGAALGGESERRRQVTTFKRILKAKSTERFDGLPYLFSLLNCLICLWYGLPWVANGRLLVTTVNGTGAVFQLAYICLFIFYADSKKTSVILPILHLILS

>OsSWEET16L

MLGVADRSTVKREEFNRGDQAKMVKVVLAVNVGALAAVVAVALVALHGGVRLFVVGVLCAALTIGMYAAPMAAMRTVVKTRSVEYMPFSLSFFLFLNGGVWSVYSLLVKDYFIGIPNAIGFALGTAQLALYMAYRRTKKPAGKGGDDDEDDEEAQGVARLMGHQVEMAQQRRDQQLRKGLSLSLPKPAAPLHGGLDRIIKSFSTTPIELHSILHQHHGGHHHHHRFDTVPDDDDEAVAAGGTTPATTAGPGDRH

>VvSWEET1

MDAHHALHFTFGIFGNATALFLFLAPLITFKRIIKSKSTEQFSGIPYVMTLLNCLLSAWYGLPFVSKNNILVSTINGTGAAIEIIYVLIFIAYSIKKERAKILGLFIFVLSVFGVVVFVSLFALHGHSRKLFCGLAATIFSIIMYASPLSIMRMVIKTKSVEYMPFFLSLFVFLCGTSWFVFGLLGKDPFVAVPNGFGCGLGAMQLILYAIYCKKGKSKNLAAADKPVDMELGKPQQEKQSRAQNGNV

>VvSWEET2a

MSRSLLLPVNTICKDAAGVAGNIFAFGLFVSPIPTFRRIARNRSTESFSGLPYIYALLNCLVTLWYGTPLVSYNNIMVTTVNSMGAAFQLVYIILFITYTDKRKKVRMFGLLMVDIVLFLVIVVGSLEISDFTIRRMVVGFLSCAALISMFASPLFVINLVIQTRSVEFMPFYLSLSTFLMSASFLAYGILNNDPFVYVPNGAGTVLGIVQLGLYSYYKRTSAEESREPLIVSYG

>VvSWEET2b

MSSVYSVCCDAAGIAGNLSAFVLFVSPIPTFRRIIRNGSTEQFSGLPYIYALLNCLICLWYGMPLVSPGIILVATVNSVGAIFQLIYIGIFITFAEKAKKMKMSGLLTAIFGIYAIIVFASMKLFDPHARQLFVGYLSVASLISMFASPLFIINLVIRTRSVEYMPFYLSLSTFLMSLSFFTYGMFKHDPFIYVPNGIGTILGVVQLVLYAYYSRTSTEDLGLRESFIESYA

>VvSWEET3

MGDRLHLAIGVMGNAASLLLYTAPILTFARVMRKKSTEEFSCIPYIIALLNCLLYTWYGLPVVSYRWENFPVVTINGLGILLFSFILIYFWFTSPRGKIKVVGTVVPVVTVFCITAIISSFVLHDHHHRKMFVGSVGLVASVAMYGSPLVVVRQVILTKSVEFMPFYLSFFSFLTSFLWMAYGLLGHDLLLASPNLVGSPLGILQLVLYCKYRKRGIMEEPNKWDLEGNDEKSKQLQPVINNDSNGKI

>VvSWEET4

MTGADTARTVIGIIGNVISFALFASPSPTFWRIWKKRSVEEFSPDPYLATVMNCMFWIFYGLPVVHPNSTLVVTINSIGLAVELIYLTIYFVFAPNKGRLKVIGVLCLELAFMAAVVVVTLTKLHTHASRSNLVGIFCVVFGVLMYASPLTVMKKVITTKSVEYMPFYLSLTNFLNGVIWLTYALIQFDLYITIGNGLGAVSGAIQLILYACYYKSTPKDKEGKEKGKSSEVELASPKRLNKPTPQATAANTAA

>VvSWEET5a

MVNPDTIRTIVGIIGNVISFGLFASPIPTFIQIVKKKTVGEFKPDPYLATVLNCMMWVLYGLPFVRPDSLLVITINGGGLVIELIYVTIFFVYADSLKRKKIALWLLFEVIFMAIIAAITMLLFHGTKNRSLFVGLLCVVFNVIMYASPLTVMRQVIRTKSVKYMPFTLSLANFANGIVWSIYALIKFDPYILIPNGLGSLSGAVQLILYATYYKSTPKDEEDKKPPEVQLSGM

>VvSWEEET5b

MVSKDTARTIVGIIGNIISFGLFASPIPTFKKIYHEKTVGGFKPDPYLATVLNCSLWVLYGLPFVHPDSVLVITINGIGLVMEIIYVSIFFTYSDWAKRKKIVMALLCIVIFVAAVAGITMGAFHTHHDRSMFVGILCVVFNVVMYASPLTVMRRVIRTRSVKYMPFFLSLANLMNGIVWLIYALIKIDAYIVIPNALGTISGLVQMVLYAAFYKSTPREEEEVKKTQEVQLSGI

>VvSWEET7

MSSTEVARTAVGILGNIIALFLFLSPVPTFISIWKKGSVEQYSPVPYLATFINCMVWVLYGLPMVHPHSTLVVTINGTGFVIELVYLILFIVFSNRGNRLRVIMIALVEIIFVAIVALLTLTMVHTTDRRSMIVGTICILFNIMMYASPLSVMKMVIRTKSVEYMPFFLSLAAFGNGIAWTTYALIRFDLFITVPNGLGTLFAAAQLTLYAMFYKSTKRQLAERKQGKVEMDLAQVVVTAEPMDKAQNGGGGGVHEVRRT

>VvSWEET9

MAVVTVKQLAFIFGLLGNLVSFMVYLSPVPTFFKIYKRKTSEGYQALPYSVGLLCASLFLYYALLQSGKFLILSINTIGSTIQATYLVLFIIYSPRAGKVATLKMILILNVASLGLVLLLTTLFSKGKTRIQVVGWISAGVNIGTFVAPLSIIKRVIETRSVEYMPFNLSFFLTICATMWFFYGIFVRDFFIAIPNVVGFVFGIAQMFLYIIYKYMMKSDETTLEQLEETTERPLYVPTANHEPSGQELKAVTITSPRQVDYFTEHHPMFMERDEYLS

>VvSWEET10

MALFPIHHPLVFIFGILGNLISFMVYLAPLPTFYQIYKRKSTEGFQSVPYVVALFSAMLWIYYAFLNTDASLLITINSVGCVIETSYIVMFLVYAPKKARITTVKLVFLMNICGFGSILLLTLLLAEGANRVRILGWVCLVFSLSVFLAPLCIMRQVIRTKSVEYMPFLLSFFLTLSAVMWFFYGLMLKDFYIAGPNILGFVFGIVQMVLYLIYRNRKKVLENEKLPELSEQIIDVVKLSTMVCSEVNLTNQQHSNEGHGTTGLEVIVAL

>VvSWEET11

MAMLTVPHMAFAFGILGNIVSFLVYLSPLPTFYRIYKRKSTEGFQSIPYSVALFSAMLLLYYAFLKTDNQIMLITINSVGTCIEATYLLVYMIYAPRTAKIYTAKLLLLFNTGVYGAIVLSTFFLSKGHRRAKIVGWVCAAFSLCVFAAPLSIMRLVIRTKSVEYMPFPLSFFLTICAVMWFFYGLLIRDFYIAFPNILGFAFGIAQMILYTIYKNAKKGVLAEFKLQELPNGLVFPTLKKAENTDTNPNDQPEDTAMTEGGARDKAVEPSGELKHNSSSLVVRFCLRALRLSFHHVSFSRIIAYTNQRNTVNTMRVYLLYIAMYENKSILVFITLFSQIL

>VvSWEET12

MAMFTVGHHPWVFASGILGNLMSFLVYLAPIPTFTRVIKKKSTEGFQSVPYVIALFSAMLWMYYGLVNTNASFLLSVNGFGCFIEIIYISIYLIFAPRRARILTLRLLLLINLGAFCLILIVTNFMVKRPHRVKAVGWVCLIFAVSVFAAPLSIMASILYRLVIRTKSVEFMPLPLSICLTLSAVGWFFYGILQMDLYIAMPNTLGFVFGLIQMILYAMYRNSTPVTKEPKLPEQVIDIVKLNTNSTPEVHPVSTLQPNCVENEGGNGQNARKETEHAEESMGGSNRV

>VvSWEEET15

MAMAMANHHTLGLIFGILGNIISFLVYFAPAPTFYRIYKRKSAEGFHSLPYIVALFSAMLWLYYALLKKDAFLLITINSFGCAIESFYILLYFFYAPMQAKKQTLKVVISLNVGVFSILVVLIQFLLKGSNRINVFGWICASFSVAVFAAPLSIVAKVIRTKSVEFMPFSLSFFLTLSAIMWFAYGLLKNDPCVAIPNILGVILGLVQMVLYGFYRNAGKEKMEKKLPEHIIDMVMLSTLGTSDIHPIGAQQNGIKKSGSEDVKDDEETGNREKSTENSGELQPNGSTV

>VvSWEET17a

MESLSFFAGVIGNIISVLVFLAPIGTFWRIVKHRSTQDFESLPYVCTLLNSSLWTYYGIIKPGEILVATVNGFGVVVEAAYVTLFLIYAPAKMRAKTVALVSLLDVGFLAAAILVTRLALQGDTRIDALGFICSGLNIVMYGSPLAAMKTVVTTKSVEFMPFFLSFFLFLNGGIWTIYAVLVRDYFLAVPNGTGLVLGTAQLVLYAIYRNSKPSNKFSIEDGSQEEHLIASSS

>VvSWEET17b

MEGLSFFVGVIGNIISVTVVLSPIKTFLRIVKHRSTEDFESFPYVIALLGTSLWCYYGVIKPGGFILATTNGLGIIIELVYVTLFIIYAPLRVRAKTAIYLGILNVAVPAIVILITLFTMHGDLRIDVLGFVCAGLSIVMYGSPLVVVKRVLTTKSVEYMPFLLSFFFFLNGGIWTVYAILVKDFFLGVPNGIGFLLGTAQMVLYAMYWKSKSSQNISEECQMGLDFFLEQLRWCSMPYTGNLSHPKIFQSWRMDGNTSISYPKTVLKTRCLTFMEERKEKNWNIISVLYMLSPVPTFSRIVKHRSTEEFESLPYVSSLATSSLWVFYGLMKSGGLLIATVNGFGIIIELVYVILFLIFAPTRMRAKTAILVVTLNVGFPAGVVLITLIVMDGDLRLDVLGIVCAVLNILMYGSPFTAMKKVVMTKSVEYMPFLLSFFLLLNGAIWTFYAILVKDFFVGVPNGIGFILGAAQIVLYAMYWKSKTSQNLSDKLKGRSMDSATSQRLSISLAATLSLILIHLTLVNSFTEIPTAYEVLEDYNFPVGLLPEEVVTGYKLNHRTGEFSAYLNDSCSVYEGGYRLKYEPTIKGYISNGKISSLEGVSVMFFHKWRKIVEILRRDNHIHFSAGVARDRFHIKDFEESPQCVCSMNLLDLSH

>VvSWEET17c

MASLSFIIGIIGNVISILVFASPIGTFRRVVKKKSTENYKGIPYITTLLSTSLWSFYGILKPGGLLVLTVNGAGAIMQFIYVTLFLIYAPRDVKIKSMKVAAVLDVGFLGAVIALTLLAFHGSSRLICVGIFCAGLTIVMYASPLSAMRMVIKTKSVEFMPFFLSFFLFLNGGVWSVYAVLVTDFFIGVPNAVGFVLGSAQLILYAVYRNKSRPSATSEERVEEEGSAHTVKRAVEMQVSKDDGKASPKNHSLSKGRSLPMPFISRQYSLQKIMRTLSWSPCELQDRQQDKDIEKGDI

>VvSUT1/VvSUC11

MAVPGGHRQRGRPRALIGEPVRPRVPLRRLLRVASVACGIQFGWALQLSLLTPYVQELGIPHAWSSIIWLCGPLSGLLVQPLVGHLSDRCNSRFGRRRPFIVAGATSIVVAVLIIGFSADIGGLLGDGADRRPRAVATFVVGFWLLDVANNVTQGPCRALLADLTEKDHRRTRVANAYFSLFIAVGNVLGFATGSYSGWFRIFWFTSTSSCNADCANLKSAFLLDIIFIAITTYISITAAQELPLSSSSRSTHISEEMAESTHAQEAFLWELFGTLRYLSGSIWIILFVTALTWIGWFPFLLFDTDWMGREIYGGKPNEGQNYNTGVRMGALGLMLNSVVLGITSVLMEKLCRKWGAGFVWGLSNILMSLCFLLMLILSAVVKHMDFLGHDLPPSGVVIAALIVFSILGIPLAITYSVPYALISTRIESLGLGQGLSMGVLNLAIVIPQVIVSLGSGPWDQLFGGGNSPSLAVAAVAAFASGLVAILAIPRSSADKSRVHT

>VvSUT2

MEATSVKAGMQPASEDAHHHQSTPLSKIILVASIAAGVQFGWALQLSLLTPYVQLLGVPHTWASFIWLCGPISGMLIQPTVGYYSDHCNSQWGRRRPFIIVGTILVTLAVILIGFAADIGKSAGDPPDKVPKVRAVVVFVLGFWVLDVANNMMQGPCRALLADMSGHNHKKTRTANSYYSFFMAVGNVLGYAAGSYTDLYKVFPFTKTKACDVYCANLKTCFIFAIILLLVLTTAAMTLVKERPLVLTQQYNADQDEEDEEEVSMPFFGQILSALGNLSRSMWMLIVVTSLNWLAWFGFLLFDTDWMGKEVYGGTVKGKESKLYDRGVHAGSLGLMLNSLVLGLMSLAIEPAARLMGGVKRVWGIGNFILAICLGLTVAVTKMAQSSRHEAAAEGRSLMPPANVKIFALTIFALLGIPQAITYSIPFALASIYSNASGAGQGLSLGVLNMAIVLPQILVSAVSGLLDDLFGGGNLPVFVAGAIAAAASGVFALTILPSPPAQPSRL

>VvSUC3

MPETMDAPSIRVPYKNLKQAEVELVAADEPRHGADLNSRVPNGTSDPSSSPSSITHPPKHGGLRTLILSCMIAAGVQFGWALQLSLLTPYIQTLGIEHAFSSFIWLCGPITGLVVQPCVGIWSDKCSSKYGRRRPFILAGSLMISVAVTIIGFSADIGYLLGDTNMDCRKFKGTRTWAAIIFVLGFWMLDLANNTVQGPARALLADLSGPDQRNSANAIFCSWMAVGNILGFSAGASGHWHRWFPFLLNKACCEACGNLKAAFLIAVVFLTLCTLVTLYFAEEVPLMAYQPHHLSDSAPLLDNPQQIGFDNSKSKLDMSAVDNATGNNPESSYEINKNAKHLTPIVQEQNESFSDGPGAVLVNLLTSLRHLPPAMHSVLLVMALSWLSWFPFFLFDTDWMGREVYHGDPKGDESAVKAYDAGVREGAFGLLLNSVVLGISSFLIEPMCQRMGARLVWAMSNFIVFACMAGTAIISLVSVNKYITEGIQHAIGENRAIKIASLVVFALLGFPLSITYSVPFSITAELTADTGGGQGLAIGVLNLAIVIPQMIVSLGAGPWDALFGGGNIPAFVLAALFALAAGVIAILKLPNLSSSSYKSSGFHFG

>VvSUC27

MELAKPSSVFAIQDHQSSSPPTPIWKTVVVASIAAGIQFGWALQLSLLTPYVQLLGIPHKWAAFIWLCGPISGMIVQPVVGYHSDRCTSRFGRRRPFIAAGAVLVAIAVFLIGYAADIGRVSGDPLHNTIKTRAVAVFVVGFWILDVANNMLQGPCRALLADLSGTSARRTRTANALYSFFMAVGNVLGYAAGSFSKLHKMFPFARTQACDLYCANLKSCFFLSIALLLILTIIAFATVHETPLNRADIAVVEAGQPFYSQMMNAFRQLRRPMWVLLLVTCLNWIGWFPFLLFDTDWMGREVYGGTVGEGPRGRLYDLGVRAGSLGLMLNSVVLGLMSLGVEFFGRGVGGVKRLWGGVNFLLALCLALTVLVSKLAASWRHSLGGELHPPPIGIKAGALSLFAVMGVPLAITYSIPFALASIFCHSSGAGQGLSLGVLNLAIVVPQMMVSVASGPWDARFGGGNLPAFVVGAFAAALSGVLALTMLPAPPPDVPNTKDERTQPSS

>VvSUCy

MEATSVKAGMQPASEDAHHHQSTPLSKIILVASIAAGVQFGWALQLSLLTPYVQLLGVPHTWASFIWLCGPISGMLIQPTVGYYSDHCNSQWGRRRPFIIVGTILVTLAVILIGFAADIGKSAGDPPDKVPKVRAVVVFVLGFWVLDVANNMMQGPCRALLADMSGHNHKKTRTANSYYSFFMAVGNVLGYAAGSYTDLYKVFPFTKTKACDVYCANLKTCFIFAIILLLVLTTAAMTLVKERPLVLTQQYNADQDEEDEEEVSMPFFGQILSALGNLSRSMWMLIVVTSLNWLAWFGFLLFDTDWMGKEVYGGTVKGKESKLYDRGVHAGSLGLMLNSLVLGLMSLAIEPAARLMGGVKRVWGIGNFILAICLGLTVAVTKMAQSSRHEAAAEGRSLMPPANVKIFALTIFALLGIPQAITYSIPFALASIYSNASGAGQGLSLGVLNMAIVLPQILVSAVSGLLDDLFGGGNLPVFVAGAIAAAASGVFALTILPSPPAQPSRL

>VvHT1

MPAVGGFDKGTGKAYPGNLTPYVTVTCVVAAMGGLIFGYDIGISGGVTSMAPFLQKFFPSVYRKEALDKSTNQYCKFDSETLTLFTSSLYLAALLSSLVAATVTRKFGRKLSMLFGGLLFCAGAIINGAAKAVWMLIVGRILLGFGIGFANQSVPLYLSEMAPYKYRGALNIGFQLSITIGILVANILNYFFAKIKGGWGWRLSLGGAVVPALIITVGSLVLPDTPNSMIERGQHEGAKTKLRRIRGVDDVEEEFNDLVVASEASKLVEHPWRNLFQRKYRPHLTMAILIPFFQQLTGINVIMFYAPVLFKTIGFADDASLMSAVITGGVNVLATIVSIYGVDKWGRRFLFLEGGTQMLICQIIVATCIGVKFGVDGEPGALPKWYAIVVVLFICVYVSGFAWSWGPLGWLVPSEIFPLEIRSAAQSVNVSVNMFFTFIIAQIFLNMLCHMKFGLFLFFAFFVVVMSFFIYFFLPETKGIPIEEMAEVWKSHWFWSRYVNDGSYSGVELVKENYPVKNV

>VvHT2

MAVGGFAVDDNSRAFSGKVTASVVITCIVAASGGLIFGYDIGISGGVTTMQPFLKKFFPVVLRKAADAKTNIYCVYDSHVLTAFTSSLYIAGLAASLVASRLTRAVGRRNTMIIGGLTFLIGAALNGGAENVAMLILGRILLGFGVGFTNQATPIYLSEMAPPKWRGAFGTSFQFFIGIGVVVANCLNYGTAKISWGWRLSLGLAIVPSVIMTVGALLISDTPSSLVERGKVAQARDSLRKARGKDIDIEPELAELVKTSEAVKAANEEPFVTIFERQYRPHLVMAFAIPFFQQLTGINIIAFYAPVLFQSVGFGSDSALIASIILGCVNLLSIIVSTFIVDRYGRRILFLEGGTQMIIGQVAVACVLAVTTGVSGTKDIPRGYAVLVLVLMCIYAAGFGWSWGPLSWLIPSEIFPMKIRTTGQAISVAVNFATTFVLAQTFLTMLCHFKYGTFLFYAGWLIAMTLFVILFVPETKGIPLESMYQVWERHWFWRRFVSLP

>VvHT3

MEVGDGSFAPVGVSKQRADQYKGRLTTYVVVACLVAAVGGAIFGYDIGVSGGVTSMDTFLEKFFHTVYLKKRRAEEDHYCKYNDQGLAAFTSSLYLAGLVASIVASPITRKYGRRASIVCGGISFLIGAALNAAAVNLAMLLSGRIMLGIGIGFGDQAVPLYLSEMAPAHLRGALNMMFQLATTTGIFTANMINYGTAKLPSWGWRLSLGLAALPAILMTVGGLFLPETPNSLIERGSREKGRRVLERIRGTNEVDAEFEDIVDASELANSIKHPFRNILERRNRPQLVMAICMPAFQILNGINSILFYAPVLFQTMGFGNATLYSSALTGAVLVLSTVVSIGLVDRLGRRVLLISGGIQMVLCQVTVAIILGVKFGSNDELSKGYSVLVVIVICLFVIAFGWSWGPLGWTVPSEIFPLETRSAGQSITVAVNLLFTFIIAQCFLSMLCSFKHGIFLFFAGWIVIMTLFVYFFLPETKGVPIEEMIFVWKKHWFWKRMVPGTPDVDDIDGLGSHSMESGGKTKLGS

>VvHT4

MAVGIAVTSHGGHYNGRITLFVVLSCMMAGMGGVIFGYDIGISGGVTSMDSFLKKFFPEVYKRMKEDTKISNYCKFDSQLLTSFTSSLYIAGLVASFVASWITKKFGRKPTILAGGAAFLIGSALGGAAFNVYMVILGRILLGVGVGFANQAVPLYLSEMAPPRYRGAINNGFQFSIGVGALSANLINFGTEKIKGGWGWRVSLALAAVPASILTLGALFLPETPNSLIQRSKDYGKAELMLQRVRGTNDVQAELDDLVKASSLAKTINDPFKKILQRKYRPQLVMAIAIPFFQQVTGINVIAFYAPVLFRAIGLGVSASLLSAVVTGVVGMASTFISMLIVDKLGRRVLFLVGGIQMLVSQIMVGGILAAELGDHGGVSKVYAFLVLLLICVYVAGFGWSWGPLGWLVPSEIFPLEIRSAGQSITVAVSFIFTFIVAQTFLSMLCHFKSGIFFFFGGWVVLMTAFVYYLLPETKSIPIEQMDRVWKEHWFWKRIVVEELSNPKMETA

>VvHT5

MPAGGFAAPSAGGDFEAKITPIVIISCIMAATGGLMFGYDVGVSGGVTSMDPFLKKFFPVVYRKQHEELESNYCKYDNQGLQLFTSSLYLAGLTSTFFASYTTRSFGRKATMLIAGIFFIVGVVLNTAAQDLAMLIIGRILLGCGVGFANQAVPLFLSEIAPTRIRGGLNILFQLNVTIGILFANLVNYGTAKIKGGWGWRVSLGLAGIPAVLLTVGSLLVVDTPNSLIERGRLEEGKAVLRKIRGTDKIEPEYQELLEASRVAKLVKHPFRNLMQRRNRPQLIIAVALQIFQQFTGINAIMFYAPVLFDTLGFGSDASLYSAVITGAVNVLSTLVSVYSVDKVGRRLLLLEAGVQMFFSQVVIAIILGIKVKDHSNNLHTGYAVLVVVLVCTFVAGFAWSWGPLGWLIPSETFPLETRSAGQSVTVCVNLLFTFVIAQSFLSMLCHLKYGIFLFFSGWVFIMSFFVLFLLPETKNIPIEEMTERVWKKHWLWKRFMDDHVEGFPVFGYNDEETVVNGSDKKRDGYGNGFDPSSQL

>VvHT9

MAPLVGIKAGGFMGPSGGDHVEYPGKLTWSVLISCVLGAMGGLIFGYDIGISGGVTSMPTFLEKFFPSVYKKEELDKSTNQYCKFDSQILTLFTSSLYLAALVSSLVASYATRRFGRRVSMLVGGLIFMAGAILNAFAVNILMLIFGRILLGFGVGFATQSVPIYVSEMAPYKHRGALNNVFQLSITIGILVANVVNYFTAKIEGGWGWRVSLGGAAIPAIFISAVAWILPNTPNSMIEKGELQQAREMLCRIRGVSDREIEAEYIDLVAASEASKRVQHPWRNLRLSEYRPQLVMSILIPALQQLTGINVVMFYAPVLFQSLGFGNNASLFSAVITGLVNMLATFVAVFGTDKWGRRKLFIEGGIQMLIFQVAVAVLIALKFGVSGNVTELPEWYSIIVVMCICIYVSAFAWSWGPLGWLVPSEIFPLEIRSAAQSITVSVNMFFTFGVAEVFLSMLCGLKYGLFIFFSVFVAIMTVFIYVFLPETKGIPIEEMRVVWKRHWYWKRFMPDYDDQQGKQNKKEKP

>VvHT11 MAASIWLGPRDDGDNHPSKLTRFDYITCVFASMGGLMFGYDIGISGGVTSMADFLKKFFPTIFQRDPVERSGNQYCKFNSHTLTLFTSSLYLAALASSLIASCATRRFGRKISMLIGGLVFLAGAVFNVLAMQVWMLIVGRLLLGLGVGFAIQSVPIYVSEMAPYKHRGALNNLFQLSITLGILIANVVNYFTVKIHGGWGWRVSLGGAAVPAIFLSAVAWIIPNTPNSMIEKGELRQAREMLRRIRGVSDDRIEAEFRNLVAASEASKEVLNPWRNLLQRKYRPQLVMSILIPAFQQLTGINVVMFYAPVLFQSLGFGSNASLFSAVVSGLVNVGATLVAVYGADKWGRRKLFLEGGIQMLVFQVALAVLIALKFGVTGTASHLPHWYSTVVVVCICGYVAAFAWSWGPLGWLVPSEIFPLEIRSAAQSIAVSVNMLFTFLVAEVFLSMLCGLKSGFFIFFAALVTIMTVFVYMFVPETKNIPIENMTEVWKRHWYWKRFMPAQDNVLEFYRSVVNALGLPMDVGNCPGKGNPK

>VvHT12

MTTTEGNSMKFESRITFYVVLCWILAAFGGLMFGYDIGISGGVTGMDGFLIKFFPIVYKRKLRAKEDNYCKYDDQYLQLFTSSLYLAALISSFPASKVCTKFGRKPTILVASVFFLLGSGLSAGAHQMWMLILGRISLGCGVGFGNEAVPLFLSEIAPVEYRGAVNILFQLFITIGILIANLVNYGTSKVHPWGWRLSLGLAAIPATGLFIGSLIIPETPTSLVERNHEEKGRKTLKKIRGVDNVDPEFEQIKVACEIARRVKHPYRSLMKLSSMPPLIIGIMMQVFQQFTGINAIMFYAPILFQTVGFKNDASLLSAIITGLVNVFCTVVSIYAVDKVGRRLLLLQACVQMFVSQTAIGGILLAKLNATNSLPKGQAWVVVVLVCVYVSSFAWSWGPLGWLIPSETFPLETRTAGFAFAVSSNMLFTFVIAQSFLSMMCHMRAGIFLFFAGWIVIMGIFVLFLLPETKGVPIDEMKERVWKKHPIWKKFMSDDADDRAKKTIEMS

>VvHT13

MAGGAFEDTEAAKRAHLYEYKITGYFIFSCIVAASGGALFGYDLGVSGGVTSMDDFLKRFFPKVYRRKQEHLKETDYCKYESQILTLFTSSLYFAGLVSTFAASHVTRKKGRKASILVGSISFFLGAVLNAAAVNIAMLIIGRILLGVGIGFGNQAVPLYLSEMSPAKIRGAVNQLFQLSTCLGILVANFINYETDKLHPWGWRLSLGLATVPATVMFLGGLALPETPNSLVEQGKFEEARKVLEKVRGTSKIEAEFADLVDASKAAQAIKHPFRNLLKRRNRPQLIIGALGIPAFQQLTGMNSILFYAPVIFQSLGFGSNASLYSSLITSGALVLASLISMAFVDRWGRRKFFLEAGCEMICYMVAVAITLALEFGQGKTLPKGTSYFLVIIISLFVLAYGRSWGPLGWLVPSELFPLETRSAGQSMVVCVNLFFTALIAQCFLVSLCHLRYGIFLVFAGLIIIMSCFIYFLLPETKQVPIEEVCYLWSKHPIWKKIVGDEPRTEGKSAEN

>VvHT14 MTKIEKVGSFESKITVYVVVCWVLAACGGLMFGYDIGISGGVTAMDDFLIKFFPAVYQRKLRAKEDNYCKYDNQYLQLFTSSLYLAALVSSFAASKMCSKLGRKPTIFVASAFFLCGSLLSAAAQRIWMIILARVLLGVGVGFGNEAVPLFLSEIAPVQHRGAVNILFQLFITIGILFANLVNYGASKIHPWGWRLSLGLASLPAAFLFVGSVVIIETPASLVERNQESQGRSTLKKIRGVEDVDAEFEQIKMACEAAREVKDPFKRLMKRSSMPPLIIGVMMQVFQQFTGINAIMFYAPVLFQTVGFKNDASLLSSVITGLVNVFSTLVSIYGVDRVGRRKLLLQACVQMFISQTAIGAILLVHLKGSNSLDEGLAGLVVVLVCLFVMSFAWSWGPLGWLIPSETFPLEIRTSGFACAVSSNMLFTFIIAQAFLSMMCHMRAFIFFFFAAWIVAMGLFVLFLLPETKNVPIDAMVERVWKQHPVWKRFMDDYDGKEDVKNVGMII

>VvHT20 MTKIEKVGSFESKITVYVVVCWVLAACGGLMFGYDIGISGGVTAMDDFLIKFFPAVYQRKLRAKEDNYCKYDNQYLQLFTSSLYLAALVSSFAASKMCSKLGRKPTIFVASAFFLCGSLLSAAAQRIWMIILARVLLGVGVGFGNEAVPLFLSEIAPVQHRGAVNILFQLFITIGILFANLVNYGASKIHPWGWRLSLGLASLPAAFLFVGSVTAIGAILLVHLKGSNSLDEGLAGLVVVLVCLFVMSFAWSWGPLGWLIPSETFPLEIRTSGFACAVSSNMLFTFIIAQAFLSMMCHMRAFIFFFFAAWIVVMGLFVLFLLPETKNVPIDAMVERVWKQHPVWKRFMDDYDGK

>VvHT21 MGVAGGVTAMDDFLIKFFPAVYQRKLRAKEDNYCKYDNQYLQLFTSSLYLAALVSSFAASKMCSKLGRKPTIFVASAFFLCGSLLSAAAQRIWMIILARVLLGVGVGFGNEAVPLFLSEIAPVQHRGAVNILFQLFITIGILFANLVNYGASKIHPWGWRLSLGLASLPAAFLFVGSVVIIETPASLVERNQESQGLSTLKKIRGVEDVDAEFEQIKMACEAAREVKDPFKTLMKRSSMPPLIIGVMMQVFQQFTGINAIMFYAPVLFQTVGFKNDASLLSSVITGLVNVFSTLVSIYGVDRVGRRKLLLQACVQMFISQTAIGAILLVHLKGSNSLDEGLAGLVVVLVCLFVMSFAWSWGPLGWLIPSETFPLEIRTSGFACAVSSNMLFTFIIAQAFLSMMCHMRAFIFFFFAAWIVVMGLFVLFLLPETKNVPIDAMVERVWKQHPVWKRFMDDYDGKEGVKNVGMII

>VvHT15

MGGLIFGYDIGISGGVTSMPTFLEKFFPSVYKKEELDKSTNQYCKFDSQILTLFTSSLYLAALVSSLVASYATRRFGRRLSMLVGGLIFMVGAILNAFAVNILMLIFGRILLGFGVGFATQAVPIYVSEMAPYKHRGALNNVFQLSITIGILVANVVNYFTAKIEGGWGWRVSLGGAAIPAVFISVVAWILPNTPNSMIEKGELQQAREMLCRIRGVSDREIEAEYIDLVAASEASRRVQHPWRNLRLREYRPQLVMSILIPALQQLTGINVVMFYAPVLFQSLGFGNNASLFSAVITGLVNMLATFVAVFGTDKWGRRKLFIEGGIQMLIFQVAVAVLIALKFGVSGNVTELPEWYSIIVVMCICIYVSAFAWSWGPLGWLVPSEIFPLEIRSAAQSITVSVNMFFTFGVAEVFLSMLCGLKYGLFIFFSVFVAIMTVFIYVFLPETKGIPIEEMRVVWKRHWYWKRFMPDHDDQQVNGNSV

>VvHT16 MTKIEKVGSFESKITVYVVVCWVLAACGGLMFGYDIGISGGVTAMDDFLIKFFPAVYQRKLRAKEDNYCKYDNQYLQLFTSSLYLAALVSSFAASKMCSKLGRKPTIFVASAFFLCGSLLSAAAQRIWMIILARVLLGVGVGFGNEAVPLFLSEIAPVQHRGAVNILFQLFITIGILFANLVNYGASKIHPWGWRLSLGLASLPAAFLFVGSVVIIETPASLVERNQESQGLSTLKKIRGVEDVDAEFEQIKMACEAAREVKDPFKRLMKRSSMPPLIIGVMMQVFQQFTGINAIMFYAPVLFQTVGFKNDASLLSSVITGLVNVFSTLVSIYGVDRVGRRKLLLQACVQMFISQTAIGAILLVHLKGSNSLDEGLAGLVVVLVCLFVMSFAWSWGPLGWLIPSETFPLEIRTSGFACAVSSNMLFTFIIAQAFLSMMCHMRAFIFFFFAAWIVAMGLFVLFLLPETKNVPIDAMVERVWKQHPVWKRFMDDYDALYQSECK

>VvHT17 MTKIEKVGSFESKITVYVVVCWVLAACGGLMFGYDIGISGGVTAMDDFLIKFFPAVYQRKLRAKEDNYCKYDNQYLQLFTSSLYLAALISSFAASKMCSKLGRKPTIFVASAFFLCGSLLSAAAQRIWMIILARVLLGVGVGFGNEAVPLFLSEIAPVQHRGAVNILFQLFITIGILFANLVNYGASKIHPWGWRLSLGLASLPAAFLFVGSVVIIETPASLVERNQESQGLSTLKKIRGVEDVDAEFEHIKMACEAAREVKDPFKTLMKRSSMPPLIIGVMMQVFQQFTGINAIMFYAPVLFQTVGFKNDASLLSSVITGLVNVFSTLVSIYGVDRVGRRKLLLQACVQMFISQTAIGAILLVHLKGSNSLDEGLAGLVVVLVCLFVMSFAWSWGPLGWLIPSETFPLEIRTSGFACAVSSNMLFTFIIAQAFLSMMCHMRAFIFFFFAAWIVAMGLFVLFLLPETKNVPIDAMVERVWKQHPVWKRFMDDYDGK

>VvHT18 MATGKEKSSVVHGEAQKVVTEFDAPKKNGRNKYAIACTILASMTSILLGYDIGVMSGAAIYIKKDLKISDVEVEILVGILNVYCLFGSAAAGRTSDWIGRRYTIVLASVIFFLGALLMGFATNYVFLMVGRFVAGIGVGYALMIAPVYAAEVSPASSRGFITSFPEVFINAGILFGYISNYAFSKLPTNLGWRFMLGIGAIPSVFLALVVIAMPESPRWLVMQGQLGLAKRVLDKTSDSKEESQLRLADIKAAAGIPEECTDDVVAVPKRSHGEGVWRELLIFPTPSVRRILIAAVGIHFFQQASGIDAVVLYSPRIFEKAGIKDDEHILLATVAVGFVKTCFILVATFLLDRVGRRPLLLTSVAGMIFSLAALGMGLTVIDHSDTKLIWAVALSLCTVLSYVAFFSIGMGPITWVYSSEIFPLRLRAQGCSIGVGVNRVTSGVLSMTFISLYKAITIGGAFFLYSGVALVGWIFFYTWLPETQGRTLEDMEILFTNSSWNKKKSSTNDTSGNSNDHINGQIQLGTNG

>VvpGlcT4

MWAATLVHQPLYVVPKILSPQSNPKLFAYPLIKSKPKCRRFGFRSRSKKLEVSAAKEQLPELHAQKPGAKEVATEEEDGDEGFDLGWLPAFPHVLIASMSNFLFGYHIGVMNGPIVSVARELGFEGNSILEGLVVSIFIGGAFIGSLSSGLLVDKFGCRRTLQIDTIPLILGALISAQAHSLDEILWGRFLVGLGIGVNTVLVPIYISEVAPTKYRGSLGTLCQIGTCLGIIVSLFLGIPSEDDPHWWRTMLYIATIPGFIISLGMQFAVESPRWLCKAGRLNEAKTIIRSLWGVSEVDRAIEEFQAVIKNDGSDLDSNWLELLEEPHSRVAFIGGTLFFLQQFAGINGVLYFSSLTFQDVGITSGALASLFVGVTNFAGALCALYLMDRQGRQRLLIGSYLGMAVSMFLIVYAIISPVDEQLGHNLSILGTLMYIFSFAIGAGPVTGLIIPELSSTQTRGKIMGFSFSVHWVCNFVVGLYFLELVEKLGVAPVYASFGGVSLLSAIFAYYFIVETKGRSLEEIEMSLNRNFPSRDR

>VvpGlcT3

MWGRQGEASVTYKRVSSRDNTKVDMEESSALFQNGMGQEITNPSWKLSLPHIIVATISSFLFGYHLGVVNEPLETIALDLGFSGNTLAEGLVVSTCLGGAFIGSLFSGWIADGIGRRRAFQLCALPMIIGASVSATTKSLEGMLIGRFLVGTGMGVGPPVASLYVTEVSPAFVRGTYGSFIQLATCLGLMGALFIGIPVKAIIGWWRICFWIATVPAGILAFAMMFCAESPHWLYKKGRIAEAEAEFEKLLGGSHVKFAMADLHKSDRGDETDAVKLSELLYGRHFRVVFIGSTLFALQQLSGINAVFYFSSTVFKSAGVPSDLANVFVGIANLSGSITAMILMDKLGRKALLVWSFFGMAVAMSVQVAGASSFISGSGAVFLSVSGMLLFVLTFALGAGPVPGLLLPEIFPNRIRAKAMAVCMSVHWVINFFVGLLFLPLLEQLGPQLLYSMFCTFCLMAVVFVKRNVVETKGRSLQEIEIALLPQE

>VvpGlcT2

MRERQYKRTASKDYLTGLDREESIVRFHNVAGKESGNPSWSLSLPHILVATVCSFLFGYHLGVVNETLEIISLDLGFNGSTLAEGLVVSTCLGGAFVGSLFSGWIADGIGRRRAFQLCALPMIIGASMSATTRSLEGMLLGRFLVGTGMGIGPPVVSLYVAEVSPTFVRGTFGSFTQIATCIGLIGALLIGIPAKEIDGWWRVCFWVSAFPAAILAFLMEFSAESPHWLLKKGRAAEAEAEFEKLLGGLHVKSAIAELLKLERGEEVDAVKLSDLFFGHYFRVVFIGSSLFALQQLSGINAVFYFSSTVFKGAGVPPDLANMCVGIANLSGSIIAMILMDKLGRRVLLLVSFSGMAASMGLQVTAASSFASESGALYLSVGGMLLCVLTFSLGAGPVPGLLLAEIFPSRIRAKAMAVCLAVHWVINFFVGLLFLRLLEQIGPQILYTIFATFCLIAVAFVKKNVVETKGKSLQEXXXXXXXXXXXXXXXXXXXXXXXXXXXXXXXXXXNHSKKLRLLFLHLSREVFSCYSLYYLAL

>VvpGlT

MQTSTYAAKGSIGLELQNRRVFPRFGEFRKQSSCVKNLRVTNNTTSSGLRIGSVVMGAEFGRPRTRIEAVFRPRSVKARASGGDIEDVDVTAPQGKSSGTVLPFVGVACLGAILFGYHLGVVNGALEYLSKDLGIAENAVLQGWVVSTLLAGATLGSFTGGALADKFGRTRTFQLDAIPLAVGAFLCATAQSVQTMIIGRLLAGIGIGISSALVPLYISEISPTEIRGALGSVNQLFICIGILAALVAGLPLARNPLWWRTMFGVAVVPSILLALGMAFSPESPRWLFQQGKISEAEKSIKTLNGKERVAEVMNDLREGLQGSSEQEAGWFDLFSGRYWKVVSVGAALFLFQQLAGINAVVYYSTSVFRSAGIASDVAASALVGASNVFGTAIASSLMDRQGRKSLLITSFAGMAASMMLLSFSFTWSALAPYSGTLAVLGTVLYVLSFSLGAGPVPALLLPEIFASRIRAKAVALSLGMHWISNFVIGLYFLSVVNKFGISTVYLGFSAVCLLAVLYIAGNVVETKGRSLEEIERALNPAT

>VvTMT1/VvHT6

MNGAVLVAIAAAIGNFLQGWDNATIAGAIVYIKKELDLESTVEGLVVAMSLIGATLVTTCSGAISDWIGRRPMLIVSSMLYFISGLIMLWSPNVYVLLIARLLDGFGIGLAVTLVPIYISETAPADIRGSLNTLPQFTGSGGMFLSYCMVFGMSLLSSPSWRLMLGILSIPSLLYFALTVFYLPESPRWLVSKGRMVEAKKVLQRLRGREDVSAEMALLVEGLGIGGETSIEEYIIGPTGELTEDQDPDAVKDQIKLYGPEAGLSWVAKPVPGGQSTLSLVSRQGSLATQTLPLMDPLVTLFGSVHEKLPETGSMRSMLFPNFGSMFSTADPQIKTEQWDEESLQREGEDYASDGGGDSDHDLQSPLISRQTSSMEKDMVPPPSHSSIMSMRRHSSLMQGTAGEAAGGMGIGGGWQLAWKWSEREGEDGKKEGGFKRIYLHEEGVPGSRRGSLVSLPGGDVPAEGDYIQAAALVSQPALYSKELMDQDPVGPAMVHPAETASRGPMWAALLEPGVKHALFVGAGIQILQQFSGINGVLYYTPQILEEAGVEVLLESLGLGTESASFLISAFTTLLMLPCIVVAMKLMDIVGRRRLLLTTIPVLIVTLLVLVIGDLVTTTTVIHAAISTACVIIYFCCFVTAYGPIPNILCSEIFPTRVRGLCIAICALVYWIGDIIVTYTLPVMLTSIGLTGIFGIYAVVCVISWVFVFLKVPETKGMPLEVIAEFFAVGARQVTAAKND

>VvTMT2

MSGAVLVAIAAAVGNLLQGWDNATIAGAVLYIKKEFNLQGEPTVEGLIVAMSLIGATFITTISGAVSDWLGRRPMLIISSLFYFVSGLVMLWSPNVYVLLLARLLDGFGVGLSVTIVPVYISETAPSEIRGLLNTLPQFTGSVGMFLSYCMVFGMSLMNSPSWRLMLGVLFIPSLVYLALTVFLLPESPRWLVSKGRMLEAKHVLQRLRGREDVSGEMALLVEGLGVGSKASIEEYIIGPDDLTDDQDPAAMNDRIRLYGPQEGLSWIAKPVTGQSSLGLVSRCGSMENKPVPLMDPLVTLFGSVHEKLPETGSMRSVIFPNFSSMFSISGNQPKNEESDEESLARDGEDYPSDAAGGDSDDNLQSPLISRQNTSLEKDLMPAPTQSSNLSMRHSSLMRADGGEQVSSSMGIGGGWQLAWKWSEKEGQDGKKEGGFKRIYLHQDSIPRSQRGSLVSVPGGEVPVDGEMTCAAALVSQPALYSKELMDQNPVGPAMVHPSETAIKGPSWRDLFKPGVKHALVVGVGIQILQQFSGINGVLYYTPQILEQAGVGVILSNIGISSASTSLLISAITTLLMLPCIAVAMRLMDISGRRSLLLSTIPVLIIALSILVLGSLVNMGDVVHAAISTASVIIYFCCFVMGFGPVPNILCAEIFPTRVRGLCIAICALSFWIGDIIVTYTLPLMLTSVGLAGVFGMYAVVCLISWVFVFLKVPETKGMPLEVISEFFAVGASAGQKKN

>VvTMT3

MNGAVLVAITAAIGNLLQGWDNATIAGAVLYIKREFHLQTEPTIEGLIVAMSLIGATAITTFSGPVADWLGRRPMLIISSVLYFLSGLVMLWSPNVYVLLLARLLDGFGIGLAVTLVPVYISETAPSEIRGLLNTLPQFTGSGGMFLSYCMVFWMSLMDSPKWRLMLGVLSIPSLLYFALTVFYLPESPRWLVSKGRMAEAKQVLQRLRGREDVAGEMALLVEGLGVGGDTSIEEYMIGPADELADNQEQSTEKDQIKLYGPEQGLSWVARPVTGQSTLGLVSRHGSMANQSVPLMDPLVTLFGSVHEKFPETGSMRSMLFPNMGSMFSVAEYQDKNEQWDEESLQRDGEDYGSDGGGESDDNLRSPLLSRQTSSTEKDMVPPAANGSILNMRRHSSLMQGAAGEAGSSMGIGGGWQLAWKWSEKRGKDGNKERELQRIYLHPEDAPGSRRGSVASLPVADAPEEGGFVQASALVSQSMLYSKGGKDKHPIGPAMVQPAESVAVGPSWQDLFEPGIKRALFVGVGIQILQQFSGINGVLYYTPQILEQAGVGVLLSNMGIGSESASLLISGLTTLLMLPSIGFAMRLMDVSGRRWLLLTTLPILLLSLIILVLGNIIPMGSLVHAIISTVSVVVYFCCFVMAFGPIPNILCSEIFPTRVRGLCIAVCALTFWICDIIVTYSLPVMLSSVGLAGVFGIYAIVCILSWIFVFLKVPETKGMPLEVISEFFAVGAKQAATDAKNN
