# Supplementary material for: Identification and expression analysis of pineapple sugar transporters reveal their role in the development and environmental response
Source: Front Plant Sci. 2022 Oct 24;13:964897. doi: 10.3389/fpls.2022.964897 (PMC9638087; doi:10.3389/fpls.2022.964897)
Supplement: Additional File S2 — List of primers used for expression analysis. [file Table_2.docx]

| Name | Primer Seq 5-->3 | |
| --- | --- | --- |
| *AcSUT1* | TCGGATTCTGGCTATTGGAC | AAGGAAAGGGAACCACTCGT |
| *AcSUT2* | TTGGACCGAACGCTGCTAAT | GCTGCCGTTTGAAGGAAAGG |
| *AcITR1* | GCTGCACCTTCGGAAATTAG | AGCGAGGTGATTCAGGAAGA |
| *AcpGLCT-L1* | TCCTCATCGAACCACTGTGC | CCACACGCTTATGATTGCCG |
| *AcZIFL2a* | GATGACTGCGATGTCCCTTT | GGTAAGGACGAATCCGATGA |
| *ACZIFL2b* | GGGCCGTCAGTGATAGAAAA | AAAGCGACGAAAAGGGGTAT |
| *AcEF1a* | TCTTCTCAGGGAAGGTCTCTAC | CTCTGCACACTCTTCACATACA |
